# The Effect of Hospital‐Physician Vertical Integration on Utilization‐Driven Changes in Healthcare Spending for an All‐Payer Population With Multiple Chronic Conditions

**DOI:** 10.1111/1475-6773.70144

**Published:** 2026-07-06

**Authors:** Alexandra Harris, Neil Jordan, Cynthia Barnard, Jeffrey A. Linder, Brady Post

**Affiliations:** ^1^ Health Sciences Integrated PhD Program, Northwestern University Feinberg School of Medicine Chicago Illinois USA; ^2^ Medicare Payment Advisory Commission Washington, DC USA; ^3^ Department of Psychiatry & Behavioral Sciences and Institute for Public Health and Medicine Northwestern University Feinberg School of Medicine Chicago Illinois USA; ^4^ Center of Innovation for Complex Chronic Healthcare, Hines VA Hospital Hines Illinois USA; ^5^ Department of Medicine and Medical Social Sciences Northwestern University Feinberg School of Medicine Chicago Illinois USA; ^6^ Department of Medicine Northwestern University Feinberg School of Medicine Chicago Illinois USA; ^7^ Department of Health Sciences Northeastern University Bouvé College of Health Sciences Boston Massachusetts USA

**Keywords:** health services utilization, healthcare spending, multiple chronic conditions, provider consolidation, vertical integration

## Abstract

**Objective:**

To estimate the effect of hospital‐physician vertical integration on utilization‐driven annual healthcare spending for an all‐payer cohort of patients with multiple chronic conditions.

**Study Setting and Design:**

We used a quasi‐experimental difference‐in‐differences with staggered adoption approach to estimate the effect of hospital‐physician vertical integration on total annual utilization‐driven spending per patient (primary outcome). We also examined changes in annual inpatient, outpatient, professional, and pharmaceutical utilization‐driven spending (secondary outcomes).

**Data Sources and Analytic Sample:**

Using 2016–2021 Virginia all‐payer administrative claims data, we conducted a retrospective analysis of 77,248 patients aged 45–99, with Medicare, Medicaid, and/or commercial insurance, and at least two qualifying chronic conditions at the beginning of the study period. The treatment group included patients of physicians who began the study period independent and became integrated in 2018, 2019, 2020, or 2021, respectively. The control group included patients of physicians who remained independent during the entire study period.

**Principal Findings:**

While we found no significant difference in utilization‐driven spending after integration overall ($1063, CI: $‐364 to $2414), meaningful heterogeneity emerged across the staggered integration cohorts. Notably, when excluding the 2021 integration cohort (which exhibited significantly lower total annual utilization‐driven spending), we found evidence of substantially higher annual total ($1750, CI: $207 to $3739) and inpatient ($898, CI: $65 to $2293) spending after integration among the earlier integration cohorts, driven by inpatient utilization. These findings held when limiting our analyses to patients of primary care physicians.

**Conclusions:**

This study provides evidence that the timing of hospital‐physician vertical integration (pre‐pandemic versus pandemic‐era), particularly among primary care physicians, drove heterogeneous treatment effects in utilization‐driven spending for the growing population of adults living with multiple chronic conditions.

## Call‐Out Box

- What is known on this topic?
- Hospital‐physician vertical integration has expanded rapidly, raising concerns about its impact on healthcare prices, spending, and market power.
- Prior research shows integration is often associated with higher healthcare spending, driven by higher prices and shifting care to more expensive health care settings.
- The impact of integration on utilization‐driven spending among patients with multiple chronic conditions—who account for the highest share of costs—remains poorly understood.
- What this study found?
- The timing of integration (pre‐pandemic vs. pandemic‐era), especially among patients of integrated primary care physicians, had heterogeneous effects on utilization‐driven spending among patients of physicians.
- Hospital‐physician vertical integration, advertised by providers as a strategy to improve care coordination and efficiency, did not contain healthcare spending for adults with multiple chronic conditions.

## 1 Introduction

The integration of hospitals and physicians has emerged as a prominent trend among US health systems, with substantial interest from researchers and policymakers about whether integration can improve efficiency and contain rising costs. While proponents argue that vertical integration promotes value‐based care by facilitating care coordination and aligning financial incentives, policymakers and researchers question whether economic motivations to improve market power and increase revenue may be the driving force. The implications for healthcare spending among the costliest utilizers of health care—namely, patients with multiple chronic conditions (MCCs)—remain unclear.

Vertical integration can influence healthcare spending through competing operational and economic mechanisms [1]. From an efficiency standpoint, integration may reduce fragmentation and improve care coordination by leveraging shared electronic health records and standardized guidelines, potentially reducing unnecessary and preventable care [2, 3, 4]. However, economic theory indicates integration can also drive spending increases. Market power dynamics may allow integrated systems to negotiate higher reimbursement rates in commercial markets, while revenue‐maximization incentives may result in higher spending, either through higher negotiated prices, self‐referrals, or increased health services utilization [1, 5].

Research to date generally supports the theory that vertical integration results in higher healthcare spending. One significant driver of higher spending is the increased market power of integrated systems, which enables hospitals to negotiate higher payment rates from commercial insurers. In Medicare markets, studies have documented that vertically integrated physicians often bill for outpatient services at hospital outpatient department (HOPD) rates, rather than lower‐cost physician office rates, leading to higher prices for the same services [1, 5, 6, 7]. Additionally, concerns have been raised about the potential for integrated systems to promote higher utilization of their own facilities and services through self‐referrals, further contributing to increased spending [8]. However, there remain considerable gaps in our understanding of the ways in which vertical integration is changing healthcare delivery. Further research is needed to identify contexts where integration achieves its intended benefits, one of which may be for patients with MCCs, a population that accounts for a disproportionately high share of healthcare expenditures. This population is particularly complex due to challenges such as polypharmacy, interactions between chronic conditions, coordinated care management, and barriers to finding accessible, effective treatments. These patients often interact with multiple clinicians and care settings, making them especially sensitive to organizational change. While it may influence how and where patients access care, vertical integration's effect on utilization‐driven spending patterns for patients with MCCs remains poorly understood. Understanding how vertical integration influences utilization‐driven spending for this complex and costly patient panel is critical to informing policies that promote sustainable healthcare delivery models.

In this paper, we used advanced difference‐in‐differences (DiD) methods to estimate how hospital‐physician vertical integration changes utilization‐driven annual healthcare spending among a multi‐payer population with MCCs. By accomplishing this objective, we aimed to evaluate whether integration delivers on its potential of more efficient care for a population that drives the largest share of healthcare spending in the United States.

## 2 Methods

### 2.1 Study Design and Setting

The study cohort included 77,248 patients (attributed to 4664 unique physicians) aged 45–99, with Medicare, Medicaid, and/or commercial insurance, and at least two qualifying conditions at the beginning of the study period (2016). Qualifying conditions included the following conditions: acute myocardial infarction (AMI), alzheimer's disease and related disorders or senile dementia, atrial fibrillation, chronic kidney disease (CKD), chronic obstructive pulmonary disease (COPD) and asthma, depression, diabetes, heart failure (HF), and stroke and transient ischemic attack (TIA). Except for AMI, which only required one inpatient claim, patients must have had at least one inpatient claim or two outpatient claims with certain diagnosis codes to be considered to have a qualifying condition (detailed information and a list of diagnosis codes in Appendix A). This definition is closely aligned with the MCC admission measure cohort definition that has been used for quality reporting in the Medicare Shared Savings Program and, more recently, the Merit‐based Incentive Payment System [9, 10].

Aligned with other published work [11], we attributed each patient‐year to the qualifying physician with the plurality of the patient's evaluation and management (E&M) claims that year (denoted as the majority physician in a given patient‐year). Qualifying physicians were defined as specialties that may reasonably manage care for a patient with MCCs, including primary care, cardiology, neurology, endocrinology, nephrology, pulmonology, psychology, and psychiatry. In the event of a tie, we attributed the patient‐year to the first identified majority physician. Moreover, if a patient had different majority physicians across their respective patient‐years, we identified the majority physician that they were attributed to for at least three of the six study years, termed their “dominant” physician. This approach builds on annual attribution methods by prioritizing a sustained patient–physician relationship, which is necessary to minimize treatment misclassification in a staggered DiD framework where treatment is assigned at the physician level and adopted over time. Most patients had a dominant physician who was their attributed majority physician in at least four of six respective patient‐years. In the few instances where a patient was attributed to two majority physicians for three patient‐years each, we selected the first identified dominant physician. We dropped patients who did not have a dominant physician, since our modeling approach required stable attribution of patients to physicians throughout the study period. This excluded group was younger and more clinically complex, suggesting that our study cohort may be slightly biased toward older, but less complex MCC patients who had an established, multi‐year ambulatory relationship with a physician (see Appendix C for more details).

Finally, we restricted the cohort to patients with complete data (e.g., no missing data for model variables) for the entire study period (2016–2021) to satisfy the requirements of our analytic methodology described below. This ensured that each patient was observed in the year of MCC cohort definition and had adequate pre‐ and post‐treatment years for valid model estimation. Excluded patients—those with missing data or missing patient‐year observations—were generally older, had greater baseline morbidity, and a different mix of qualifying conditions (lower prevalence of diabetes but higher prevalence of most other conditions). While these differences may limit generalizability to all patients in our dataset, they are unlikely to bias comparisons between the treated and control groups within the study cohort.

### 2.2 Data Sources

We used 2016–2021 data from the Virginia All‐Payer Claims Database (APCD) and Medicare Data on Provider Practice and Specialty (MD‐PPAS). Claims data included inpatient, outpatient, professional, and pharmacy claims. Together, these data provided patient diagnoses and procedures, payer information, basic demographic characteristics, healthcare costs, and treating physicians. MD‐PPAS provided information about physicians, including medical specialties, employers, and integration. In addition, we used the publicly available U.S. Department of Agriculture Rural–Urban Commuting Area (RUCA) codes to define patient‐level rurality based on 5‐digit ZIP codes in each patient‐year [12]. Finally, we used 2016–2021 American Hospital Association (AHA) Annual Survey data to create a hospital‐level market concentration measure.

### 2.3 Variables

#### 2.3.1 Integration

Our independent variable was hospital‐physician vertical integration, which we defined by building upon an algorithm widely used in published literature [7, 13]. Integration was defined based on billing patterns from the MD‐PPAS, following a three‐step approach. First, physicians were classified as integrated if at least 75% of their outpatient claims were billed with a HOPD place of service (POS) code—a common practice among hospital‐owned physician groups to obtain higher Medicare reimbursement rates. Second, the definition was enhanced by flagging physicians whose legal entity names included keywords like “hospital,” “medical center,” or “system,” which likely indicated hospital employment. Third, the definition was further refined based on a manual review of over 6000 hospital‐TIN names (Appendix B). The control group comprised patients treated by and attributed to independent physicians who were never integrated during the study period (2016–2021). Our treatment group comprised patients attributed to dominant physicians who were independent at the beginning of the study period (at minimum, in 2016 and 2017) and became integrated later in the study period, beginning with year 2018. Since patients' dominant physicians may have integrated during different years of the study period, we defined four staggered adoption integration groups (2018, 2019, 2020, and 2021), based on each dominant physician's first year of integration. We excluded patients attributed to dominant physicians who were already integrated in 2016 or 2017, because they lacked 2 years of baseline pre‐integration data (see Appendix C for more details).

#### 2.3.2 Primary and Secondary Outcomes

The focus of this study was to measure changes in utilization‐driven standardized spending, which captures utilization effects and not price effects. We use the term spending throughout the manuscript for interpretability; however, because all claims are based on standardized rates (see additional information below), our spending measures capture utilization‐driven changes in resource use and are not influenced by price effects.

The primary outcome of interest was total annual mean spending per beneficiary, defined by summing allowed amounts for all available claim types (inpatient, outpatient, physician, and pharmacy) in a given calendar year. Notably, the Virginia APCD contains a standardized proxy allowed amount, which is used to calculate average annual spending for different services. The actual amount paid for each claim is not used in the analysis; rather, a proxy dollar value is assigned to each claim that is based upon Milliman's Global Relative Value Unit (RVU) methodology. This method calculates the value of each claim by multiplying the number of assigned RVUs by a conversion factor, which is derived from aggregated allowed amounts across all payers, claim types, and geographic regions. While these aggregated allowed amounts can be used to measure changes in spending, any observed spending changes are because of differences in health services utilization, not prices. The aggregated nature of this outcome obscures price variation, particularly across commercial insurers who negotiate prices with providers. Though this approach limits insight into price‐driven variation, it remains informative for understanding broader patterns in spending and health services utilization associated with integration.

To highlight possible changes across types of care, we stratified this outcome and estimated mean annual inpatient, outpatient, physician, and pharmacy spending per beneficiary as secondary outcomes. Outpatient spending included spending from outpatient facility claims and physician spending included spending from professional service claims. In line with published literature, we winsorized all spending outcomes at the 1st and 99th percentiles to reduce the influence of significant outliers [14, 15].

#### 2.3.3 Covariates

We accounted for patient‐, physician‐, and market‐level covariates that may influence the association between integration and utilization‐driven spending. Patient‐level factors included age, sex, race/ethnicity self‐reported by patients, payer (Medicare, Medicaid, or commercial), Medicare‐Medicaid dual eligibility status, number of MCCs, a binary indicator variable for each qualifying condition, and Charlson comorbidity score [16]. Race/ethnicity was defined within the APCD based on claims and included as a covariate due to well‐documented systematic differences in health care utilization and spending across subgroups that may confound the relationship between physician integration and our outcomes [17, 18]. We also included a binary indicator of whether a patient's physician was a primary care physician (PCP) or a specialist. Market‐level factors included rurality (based on the patient's 5‐digit ZIP code mapping to RUCA codes in a given year) and hospital market‐level Herfindahl–Hirschman Index (HHI) at the Health Service Area (HSA)‐level [19]. Following a method used in other published integration literature [20, 21, 22], HHIs were calculated at the HSA level, based on Medicare and Medicaid discharge data from the AHA Annual Survey. Each HSA‐year combination was used to compute the sum of squared hospital market shares based on total discharges and then scaled from 0 to 10,000. We categorized HSA‐level HHI values into market concentration tiers following recently updated Department of Justice and Federal Trade Commission thresholds: markets with HHI < 1000 were classified as unconcentrated, 1000–1799 as moderately concentrated, and ≥ 1800 as highly concentrated [19].

### 2.4 Statistical Analysis

First, we summarized the number of physicians who began independent in 2016 and became integrated during the study period. We then compared patients attributed to hospital‐integrated physicians and those attributed to independent physicians across several key variables representing patients' demographic characteristics, baseline spending, physician integration status, and comorbidities. Using STATA 18, we assessed standardized mean differences (SMDs) in all descriptive variables for the integrated and independent groups using *t*‐tests and non‐parametric tests, where appropriate. The level of statistical significance was set at alpha ≤ 0.05.

To account for selection bias and reduce covariate imbalance between integrated and non‐integrated groups, we employed a doubly robust DiD approach using stabilized inverse probability weightings (IPW), implemented via the CSDID command with option *dripw* in STATA [23, 24, 25]. Propensity scores were estimated using logistic regression, modeling the probability of integration based on the covariates described above. These scores were then used to construct inverse probability weights that reweight the comparison group to resemble the integrated group in terms of observed characteristics. We evaluated covariate balance after weighting using SMDs and variance ratios; values near zero and one, respectively, indicate adequate balance.

Following the Callaway and Sant'Anna doubly robust method (CSDiD) [23, 25], we then constructed patient‐level, staggered DiD linear regression models to estimate the association between hospital‐physician vertical integration and each spending outcome. Integration was defined at the physician level and models accounted for unobservable, time‐invariant characteristics, like year and physician fixed effects. Our regression analyses measured the change in outcomes after integration, relative to the same physician group before integration and groups that did not change integration status. In the event of a possible parallel trends violation (either through event study visual verification or conducting Wald tests) [26], we added lagged spending as a model covariate to improve the pre‐treatment trends.

As a robustness check, we additionally conducted alternative analyses at the physician‐year level using Arkhangelsky's synthetic DiD (SDiD) approach [27]. SDiD constructs synthetic control groups by optimally weighting untreated units to closely approximate treated units based on pre‐treatment outcomes, providing unbiased estimates even when treatment effects vary over time. To evaluate dynamic treatment effects, we utilized the event‐study extension of SDiD proposed by Ciccia [28], which decomposes the overall SDiD ATT into event‐study coefficients by comparing the lagged spending differences between integrated physicians and synthetic controls, relative to their pre‐integration averages. We operationalized this methodology using the ‘sdid_event’ package in STATA, enabling detailed analysis of the evolving treatment effects for each period after integration. The final reported ATT aggregates these period‐specific effects, weighting them according to the representation of each treatment cohort.

Because our data were at the patient‐year level, we aggregated covariates to create a physician‐year file. We adjusted for the same set of covariates in the SDiD models as in the CSDiD models. However, since the SDiD analyses were at the physician‐level, we added physicians' patient panel size as a covariate. For continuous variables, we calculated the mean value for each physician's patient panel each year. For categorical variables, we created dummy variables that represented the proportion of each value for each physician's patient panel each year. For example, our physician‐year file included a variable of the proportion of patients with Medicare insurance attributed to that physician in each year. Aligned with the CSDiD models, we accounted for unobservable, time‐invariant characteristics, including year and physician fixed effects. Using 250 bootstrap replications, we estimated standard errors and bootstrapped confidence intervals based on the empirical distribution of the bootstrapped estimates. These confidence intervals differ from traditional 95% intervals in that they do not assume symmetry or rely on estimated standard errors and are often preferred in settings with relatively few treated units and nonlinear weighting schemes. As a robustness check, we also conducted placebo tests for pre‐treatment periods and created event‐study plots.

This study was approved by Northwestern University's Institutional Review Board.

## 3 Results

### 3.1 Descriptive Results

After applying our cohort inclusion criteria, the study sample included 77,248 insured patients from 2016 to 2021, 78.3% of whom were attributed to a PCP (Table 1). Of these patients, 43,905 were female (56.8%) and 33,374 were male (43.2%), with a mean (SD) age of 72.6 (10.3) years and an average of 2.80 qualifying chronic conditions (Table 1). Medicare was the primary payer for 90% of the study cohort. The most common qualifying conditions in this population were diabetes (73.7%), CKD (63.6%), COPD/asthma (53.0%), and depression (40.1%). Across the baseline years, the mean (SD) total annual spending was $22,499 ($30,066).

**TABLE 1:** Sample baseline characteristics of MCC patients treated by integrated and independent physicians across study period.

Characteristic	Overall^a^ *N* = 77,248	Integrated^a^ *N* = 7412	Independent^a^ *N* = 69,836	Standardized mean difference	*p* ^b^
Number of physicians	4664	743	3930	—	—
Age	72.59 (10.28)	70.86 (10.95)	72.79 (10.21)	0.287	< 0.001
Female sex	43,904 (56.84%)	4197 (56.62%)	39,707 (56.86%)	0.005	0.700
Race	—	—	—	0.231	< 0.001
American Indian/Alaskan Native	50 (0.06%)	10 (0.13%)	40 (0.06%)	—	—
Asian American/Pacific Islander	2160 (2.80%)	470 (6.34%)	1690 (2.42%)	—	—
Black	12,509 (16.19%)	1462 (19.72%)	11,047 (15.82%)	—	—
Other	11,920 (15.43%)	1011 (13.64%)	10,909 (15.62%)	—	—
Unknown	880 (1.14%)	72 (0.97%)	808 (1.16%)	—	—
White	49,729 (64.38%)	4342 (59.19%)	45,342 (64.93%)	—	—
Medicare‐Medicaid dual eligibility	13,953 (18.06%)	1252 (16.89%)	12,701 (18.19%)	0.034	0.006
Payer type	—	—	—	0.259	< 0.001
Commercial	5528 (7.16%)	1037 (13.99%)	4491 (6.43%)	—	—
Medicaid	2189 (2.83%)	265 (3.58%)	1924 (2.76%)	—	—
Medicare	69,531 (90.01%)	6110 (82.43%)	63,421 (90.81%)	—	—
Charlson comorbidity score	3.06 (2.37)	2.87 (2.37)	3.08 (2.37)	0.169	< 0.001
Multiple chronic condition count	2.80 (1.03)	2.76 (1.01)	2.80 (1.03)	0.200	< 0.001
Multiple chronic condition	—	—	—	—	—
Acute myocardial infarction	6487 (8.40%)	450 (6.07%)	6037 (8.64%)	0.099	< 0.001
Alzheimer's disease and related disorders or senile dementia	12,788 (16.55%)	913 (12.32%)	11,875 (17.00%)	0.133	< 0.001
Atrial fibrillation	25,228 (32.66%)	1913 (25.81%)	23,315 (33.39%)	0.167	< 0.001
Chronic kidney disease	49,148 (63.62%)	4333 (58.46%)	44,815 (64.17%)	0.117	< 0.001
Chronic obstructive pulmonary disorder and asthma	40,976 (53.04%)	3489 (47.07%)	37,487 (53.68%)	0.132	< 0.001
Depression	31,004 (40.14%)	2507 (33.82%)	28,497 (40.81%)	0.145	< 0.001
Diabetes	56,891 (73.65%)	5422 (73.15%)	51,469 (73.70%)	0.012	0.308
Heart failure	28,810 (37.30%)	2134 (28.79%)	26,676 (38.20%)	0.200	< 0.001
Stroke and transient ischemic attack	19,570 (25.33%)	1418 (19.13%)	18,152 (25.99%)	0.165	< 0.001
Clinician specialty	—	—	—	−0.079	< 0.001
Specialist	16,774 (21.71%)	1397 (18.85%)	15,377 (22.02%)	—	—
Primary care	60,474 (78.29%)	6015 (81.15%)	54,459 (77.98%)	—	—
HHI score (0–10,000)	6999 (3222)	7795 (2692)	6941 (3250)	−0.286	< 0.001
Rurality	—	—	—	0.110	< 0.001
Rural	14,274 (18.48%)	1666 (22.48%)	12,608 (18.05%)	—	—
Urban	62,893 (81.42%)	5738 (77.42%)	57,155 (81.84%)	—	—
Unknown	81 (0.10%)	8 (0.11%)	73 (0.10%)	—	—
Total annual spending	$22,499 ($30,066)	$18,051 ($26,088)	$22,823 ($30,310)	0.169	< 0.001
Annual inpatient spending	$6083 ($17,367)	$4735 ($14,614)	$6182 ($17,546)	0.090	< 0.001
Annual outpatient spending	$5203 ($12,213)	$4423 ($11,287)	$5260 ($12,276)	0.071	< 0.001
Annual professional spending	$5456 ($6872)	$3911 ($5872)	$5569 ($6926)	0.258	< 0.001
Annual pharmaceutical spending	$5756 ($10,830)	$4983 ($9570)	$5812 ($10,914)	0.081	< 0.001

*Note:* These descriptive statistics are based on the pre‐treatment characteristics for the integrated group and corresponding years for the independent group.

Abbreviation: HHI, Herfindahl–Hirschman Index.

^a^ Mean (Standard Deviation [SD]); *n* (%).

^b^ Pearson's chi‐squared test, which tests the association between categorical variables by comparing observed and expected frequencies; one‐way ANOVA, which tests whether there is a difference in mean characteristics across the two groups.

We also calculated SMDs in demographic, clinical, and market characteristics between the integration and independent groups and found generally modest values with respect to SMDs (Table 1). To reduce imbalances, however, we applied IPW and observed slightly more similar groups (Appendix C). Across the four staggered integration cohorts, 743 independent physicians became integrated during the study period (Appendix C).

### 3.2 Callaway and Sant'anna DiD (CSDiD) Results

In the doubly robust CSDiD linear regression models, we found little to no evidence of differential spending as a result of integration (see Appendix D). However, in our event‐study plots, we observed evidence of differential pre‐trends across many of our outcomes, suggesting that one of the model assumptions (parallel trends) may have been violated and that factors other than vertical integration may explain observed differences. To assess the robustness of our findings, we additionally implemented the SDiD approach. SDiD is particularly useful in our setting because it reweights comparison units to match treated units on pre‐integration outcome trajectories, rather than relying exclusively on covariates. This feature is valuable in the presence of staggered integration and heterogeneous pre‐treatment dynamics, where residual differences in trends may persist despite doubly robust adjustment.

### 3.3 SDiD Event Study Results

**FIGURE 1:**
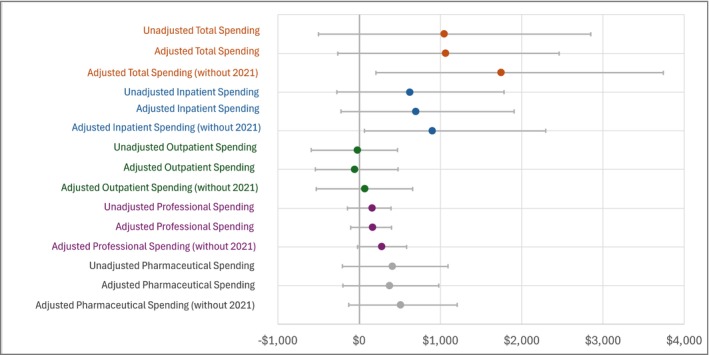
SDiD forest plot of overall ATT estimates and bootstrapped CIs for all spending outcomes. Dots represent average treatment effect on the treated (ATT) coefficients; bars represent bootstrapped confidence intervals. ATT estimates are considered statistically significant if the bars do not cross the grey vertical line at $0.

**FIGURE 2:**
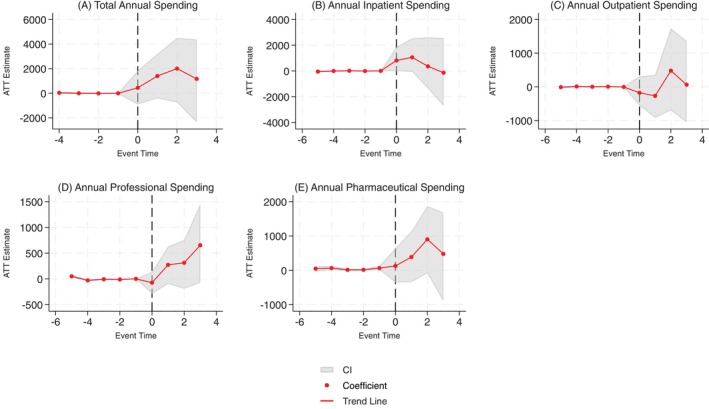
SDiD event study results for All spending outcomes. The gray shading represents the bootstrapped confidence intervals (CIs); red dots represent the average treatment effect on the treated (ATT) co efficient; the red line represents the trend over event time.

In the SDiD event study linear regression models adjusting for covariates, we did not observe statistically significant differences in total annual spending per beneficiary ($1064, Confidence Interval [CI]: $‐266 to $2459), annual inpatient spending ($696, CI: $‐221 to $1908), annual outpatient spending ($‐54, CI: $‐543 to $475), annual professional services spending ($168, CI: $‐107 to $396), or annual pharmaceutical spending ($378, CI: $‐203 to $977) after integration (Figure 1). The event‐study plots (Figure 2) show extremely consistent pre‐treatment trends. However, we did observe heterogeneity in spending changes when looking at group‐specific estimates. Across the 2018, 2019, and 2020 integration cohorts, we consistently estimated higher, albeit not always statistically significant, utilization‐driven spending across all outcomes; in contrast, for the 2021 integration cohort, we consistently estimated lower post‐integration utilization‐driven spending for each respective outcome (Appendix E).

Given that the 2021 integration cohort appeared to have the opposite trend compared to the other integration cohorts (which we believe may be driven by the recent COVID‐19 pandemic), we conducted robustness analyses by excluding the 2021 integration cohort from our models. This was especially important because the 2021 integration cohort was the largest in size, which disproportionately impacted the overall average treatment effect on the treated (ATT) estimates, as both estimation methods weight cohort‐specific estimates by the number of physicians in each cohort. As a result, the overall estimates were more reflective of the 2021 cohort's outcomes, potentially obscuring heterogeneous outcome differences across earlier cohorts.

**FIGURE 3:**
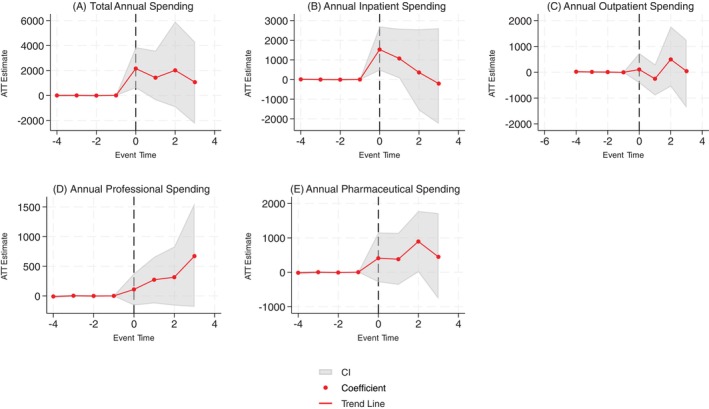
SDiD event study results for all spending outcomes (without 2021 integration cohort). The gray shading represents the bootstrapped confidence Intervals (CIs); red dots represent the average treatment effect on the treated (ATT) coefficients; the red line represents the trend over event time.

Consistent with the main SDiD event study results, our SDiD sensitivity analyses excluding the 2021 integration cohort exhibited evidence of stable pre‐trends across all spending outcomes; pre‐treatment event‐time coefficients (−5 to −1) were generally close to zero, with narrow confidence intervals crossing zero (Figures 1 and 3). In terms of our weighted overall ATT estimates, we found substantial differences in results compared to the main SDiD analyses. We found that integration resulted in $1750 higher total annual spending per beneficiary (CI: $207 to $3739) when excluding the 2021 cohort—an 8.6% increase from baseline annual spending. We found this difference to be primarily driven by a 15.5% increase in annual inpatient spending after integration ($898, CI: $65 to $2293). For both total annual spending and annual inpatient spending, we observed an immediate increase in spending during each integration cohort's first year of integration. However, this increase in spending quickly stabilized in the subsequent post‐treatment periods. Aligned with the main results, we did not find a statistically significant difference in annual outpatient, professional, or pharmaceutical spending after integration.

Finally, we conducted sensitivity analyses assessing whether utilization‐driven spending differed based on whether physicians were PCPs or specialists. We found no statistically significant difference in utilization‐driven spending when limiting to specialists; however, we found hospital‐PCP integration largely mirrored our other SDiD results—heterogeneous effects and higher total, inpatient, professional, and pharmaceutical spending among the pre‐pandemic integration cohorts. Comprehensive results, including model coefficients, standard errors, and CIs for the group‐average, overall ATT, and event‐time ATT models are included in Appendix E.

## 4 Discussion

This panel study examined changes in utilization‐driven annual spending for patients with MCCs after hospital‐physician vertical integration, using staggered adoption DiD methods. Our main results showed heterogeneity in group‐specific effects across the integration cohorts. In robustness analyses that excluded the 2021 integration cohort, our results showed integration to be associated with $1750 higher total annual spending and ~$900 higher annual inpatient spending per patient, driven by immediate increases in inpatient utilization in the first year of integration. These results held when stratifying by hospital‐integrated PCPs, suggesting that PCP integration may uniquely influence utilization‐driven spending.

Our findings suggest that hospital‐physician vertical integration may result in higher utilization‐driven spending for patients with MCCs, but not in the way we hypothesized. Aligned with some published literature [7, 14, 29, 30, 31], our a priori hypothesis was that we would observe an increase in outpatient spending and a decrease in professional spending post‐integration, as a byproduct of hospital‐integrated physicians exploiting Medicare site‐specific payment policies. However, we found no evidence of higher outpatient spending or lower professional spending in our analyses; instead, the increases in spending that we observed came from greater inpatient utilization in the pre‐pandemic years, particularly in the first year after integration.

One possible reason for this could be that we focused on a patient population new to this literature. Many of our MCC patients may require frequent, intensive hospital‐based care. The initial spike in utilization‐driven inpatient spending could be driven by factors such as catch‐up procedures, increased diagnostic testing, or stabilization of complex patients under integrated care. The spike in utilization‐driven inpatient spending does appear to taper off over time, perhaps as care becomes more efficient. For this population, integration effects may manifest more as improved access to inpatient services rather than increased outpatient care, which is often emphasized in studies of less complex patients. While consistent with prior research showing increased spending after hospital‐physician integration [7, 20], our study adds evidence that higher spending is driven by inpatient utilization rather than price.

Moreover, the heterogeneity of our cohort‐specific ATT estimates provides novel evidence that integration's impact on spending for chronically ill adults may have been influenced by external shocks, particularly the COVID‐19 pandemic. The 2021 integration cohort experienced a substantial reduction in total annual utilization‐driven spending, whereas earlier integration cohorts (2018–2020) showed either no difference, or in some cases, increases in utilization‐driven spending. Shifts in care utilization, financial constraints, and operational priorities—such as fewer elective procedures, expanded telehealth use, and temporary reimbursement changes—may have altered the financial impact of integration. It is also plausible that physicians becoming hospital‐integrated in 2021 may have done so for specific reasons, such as stable employment in a time of uncertainty, potentially leading to selection bias and different outcome trends. This pattern of heterogeneous effects is consistent with recent evidence from the integration literature. For example, a recent study examining health insurer acquisitions of physician practices found that price effects were not uniform across acquisitions, with increases concentrated in the single largest transaction, while most acquisitions showed no significant average effect [32]. Together, these findings suggest that average integration effects may mask substantial variation driven by the scale, timing, and context of specific integration events.

Our study had some limitations. First, due to existing state law, large, self‐insured employers often do but are not required to submit employees' data to the APCD; therefore, our findings may not generalize to patients working for large employers. Future research should explore whether these results hold in multi‐state and national contexts. Second, the Virginia APCD provides standardized proxy allowed amounts instead of actual paid amounts. While this improves comparability, it obscures actual differences in negotiated prices across commercial payers, as well as price variation between Medicare, Medicaid, and commercial payers. As a result, spending estimates primarily reflected changes in health services utilization rather than changes in healthcare prices. This likely led to more conservative estimates of true spending differences between integrated and independent physicians, particularly for commercially insured patients. Third, our methodology is conceptually similar to an intent‐to‐treat design since we assign patients to a dominant physician based on a sustained relationship. While this reduces post‐treatment selection bias from physician switching, subsequent care from non‐dominant physicians may introduce some measurement error and attenuate treatment effects toward the null. Fourth, although our claims‐based algorithm reliably identifies the onset of hospital ownership, it cannot distinguish whether integration occurs through acquisition of entire physician groups or through incremental employment of individual physicians. Consequently, we treat all integration events uniformly, even though these integration pathways may differ in their effects on utilization and spending—group acquisitions may be better able to rapidly internalize referral networks and alter practice‐level operations, whereas individually employed physicians may generate smaller or more gradual changes. To better understand the mechanisms underlying changes in utilization‐driven spending and their policy implications, future research should prioritize development and application of methods that distinguish group‐level from individual‐level integration and assess heterogeneity in these effects across health systems. Fifth, we are unable to estimate payer‐specific treatment effects within our physician‐year level SDiD analyses. Payer type varies within physician–years and may itself change in response to physician integration; stratifying or conditioning on payer mix post‐integration would therefore change the estimand and potentially introduce bias by conditioning on a post‐treatment variable. Although we conducted patient‐level CSDiD analyses stratified by payer type as a sensitivity analysis, these models exhibited evidence of violated parallel trends and were limited by small sample sizes within payer strata. For this reason, we chose to focus on overall spending effects at the physician level. Future work examining heterogeneity by payer would be valuable. Finally, although we made efforts to include an expansive and well‐supported group of patient, physician, and market characteristics in our modeling approaches, selection bias and unmeasured confounding may have persisted.

## 5 Conclusion

With hospital‐physician vertical integration becoming a dominant behavior in healthcare markets across the US, this study provides the first evidence that the timing of vertical integration may have heterogeneous effects on utilization‐driven healthcare spending among the growing population of adults living with MCCs—namely, that patients of physicians (especially PCPs) who became hospital‐integrated prior to the COVID‐19 pandemic had higher utilization‐driven spending. Future research should evaluate whether this heterogeneity is observed over a longer timeframe of post‐pandemic years, and whether utilization‐driven spending patterns among the pandemic‐integrated physicians rebounded as healthcare utilization normalized post‐pandemic.

## Funding

Dr. Harris was supported by the Agency for Healthcare Research and Quality under grant awards T32HS000084 and 1R36HS029643‐01A1. Dr. Post was supported by the Agency for Healthcare Research and Quality under grant award K01HS029278. This paper's contents are solely the responsibility of the authors and do not necessarily represent the official views of the AHRQ or the Medicare Payment Advisory Commission, where Dr. Harris is currently employed. All other authors received no financial support for the research, authorship, and/or publication of this article.

## Conflicts of Interest

The authors declare no conflicts of interest.

## Supporting information


**Appendix A.** Multiple chronic conditions cohort definition.
Table A1. Acute myocardial infarction cohort criteria.
Table A2. Alzheimer's and related disorders or senile dementia cohort criteria.
Table A3. Atrial fibrillation cohort criteria.
Table A4. Chronic kidney disease cohort criteria.
Table A5. Chronic obstructive pulmonary disorder (COPD) and asthma cohort criteria.
Table A6. Depression cohort criteria.
Table A7. Diabetes cohort criteria.
Table A8. Heart failure cohort criteria.
Table A9. Stroke and transient ischemic attack cohort criteria.
Appendix B. Determining hospital‐physician integration.
Appendix C. Study cohort flowchart and baseline characteristics.
Figure C1. Sample size and cohort flowchart.
Table C1. Sample baseline characteristics of MCC patients after IPW, treated by integrated and independent physicians across study period.
Table C2. Trends of newly integrated clinicians from 2016 to 2021, by integration cohort.
Appendix D. Callaway and Sant'Anna DiD (CSDiD) doubly robust spending results.
Figure D1. CSDiD forest plot of overall ATT estimates and 95% CIs for all spending outcomes.
Figure D2. CSDiD doubly robust event study plots.
Figure D3. CSDiD doubly robust event study results (without 2021 integration cohort).
Table D1. CSDiD overall and cohort‐specific total annual spending ATT estimates (unadjusted).
Table D2. CSDiD total annual spending event‐time ATT estimates (unadjusted).
Table D3. CSDiD overall and cohort‐specific total annual spending ATT estimates.
Table D4. CSDiD total annual spending event‐time ATT estimates.
Table D5. CSDiD overall and cohort‐specific total annual spending ATT estimates (excluding 2021 integration cohort).
Table D6. CSDiD total annual spending event‐time ATT estimates (excluding 2021 integration cohort).
Table D7. CSDiD overall and cohort‐specific annual inpatient spending ATT estimates (unadjusted).
Table D8. CSDiD annual inpatient spending event‐time ATT estimates (unadjusted).
Table D9. CSDiD overall and cohort‐specific annual inpatient spending ATT estimates.
Table D10. CSDiD overall annual inpatient spending event‐time ATT estimates.
Table D11. CSDiD overall and cohort‐specific annual inpatient spending ATT estimates (excluding 2021 integration cohort).
Table D12 CSDiD annual inpatient spending event‐time ATT estimates (excluding 2021 integration cohort).
Table D13. CSDiD overall and cohort‐specific annual outpatient spending ATT estimates (unadjusted).
Table D14. CSDiD annual outpatient spending event‐time ATT estimates (unadjusted).
Table D15. CSDiD overall and cohort‐specific annual outpatient spending ATT estimates.
Table D16. CSDiD annual outpatient spending event‐time ATT estimates.
Table D17. CSDiD overall and cohort‐specific annual outpatient spending ATT estimates (excluding 2021 integration cohort).
Table D18. CSDiD annual outpatient spending event‐time ATT estimates (excluding 2021 integration cohort).
Table D19. CSDiD overall and cohort‐specific annual professional spending ATT estimates (unadjusted).
Table D20. CSDiD annual professional spending event‐time ATT estimates (unadjusted).
Table D21. CSDiD overall and cohort‐specific annual professional services spending ATT estimates.
Table D22. CSDiD annual professional services spending event‐time ATT estimates.
Table D23. CSDiD overall and cohort‐specific annual professional services spending ATT estimates (excluding 2021 integration cohort).
Table D24. CSDiD annual professional services spending event‐time ATT estimates (excluding 2021 integration cohort).
Table D25. CSDiD overall and cohort‐specific annual pharmaceutical spending ATT estimates (unadjusted).
Table D26. CSDiD annual pharmaceutical spending event‐time ATT estimates (unadjusted).
Table D27. CSDiD overall and cohort‐specific annual pharmaceutical spending ATT estimates.
Table D28. CSDiD annual pharmaceutical spending event‐time ATT estimates.
Table D29. CSDiD overall and cohort‐specific annual pharmaceutical spending ATT estimates (excluding 2021 integration cohort).
Table D30. CSDiD annual pharmaceutical spending event‐time ATT estimates (excluding 2021 integration cohort).
Appendix E. Synthetic Difference‐in‐Differences (SDiD) spending results.
Table E1. SDiD overall and event time total annual spending ATT estimates (unadjusted).
Table E2. SDiD overall and event time total annual spending ATT estimates.
Table E3. SDiD overall and event time total annual spending ATT estimates (without 2021 integration cohort).
Table E4. SDiD overall and event time annual inpatient spending ATT estimates (unadjusted).
Table E5. SDiD overall and event time annual inpatient spending ATT estimates.
Table E6. SDiD overall and event time annual inpatient spending ATT estimates (without 2021 integration cohort).
Table E7. SDiD overall and event time annual outpatient spending ATT estimates (unadjusted).
Table E8. SDiD overall and event time annual outpatient spending ATT estimates.
Table E9. SDiD Overall and event time annual outpatient spending ATT estimates (without 2021 integration cohort).
Table E10. SDiD Overall and event time annual professional spending ATT estimates (unadjusted).
Table E11. SDiD Overall and event time annual professional spending ATT estimates.
Table E12. SDiD Overall and event time annual professional spending ATT estimates (without 2021 integration cohort).
Table E13. SDiD overall and event time annual pharmaceutical spending ATT estimates (unadjusted).
Table E14. SDiD overall and event time annual pharmaceutical spending ATT estimates.
Table E15. SDiD overall and event time annual pharmaceutical spending ATT estimates (without 2021 integration cohort).
Table E16. SDiD overall and event time total annual spending ATT estimates (Specialist Only).
Table E17. SDiD overall and event time total annual spending ATT estimates (without 2021 integration cohort, specialist only).
Table E18. SDiD overall and event time annual inpatient spending ATT estimates (specialist only).
Table E19. SDiD overall and event time annual inpatient spending ATT estimates (without 2021 integration cohort, specialist only).
Table E20. SDiD overall and event time annual outpatient spending ATT estimates (specialist only).
Table E21. SDiD overall and event time annual outpatient spending ATT estimates (without 2021 integration cohort, specialist only).
Table E22. SDiD overall and event time annual professional spending ATT Estimates (Specialist Only).
Table E23. SDiD overall and event time annual professional spending ATT estimates (without 2021 integration cohort, specialist only).
Table E24. SDiD overall and event time annual pharmaceutical spending ATT estimates (specialist only).
Table E25. SDiD overall and event time annual pharmaceutical spending ATT estimates (without 2021 integration cohort, specialist only).
Table E26. SDiD overall and event time total annual spending ATT estimates (PCP Only).
Table E27. SDiD overall and event time total annual spending ATT estimates (without 2021 integration cohort, PCP only).
Table E28. SDiD overall and event time annual inpatient spending ATT estimates (PCP only).
Table E29. SDiD overall and event time annual inpatient spending ATT estimates (without 2021 integration cohort, PCP only).
Table E30. SDiD overall and event time annual outpatient spending ATT estimates (PCP only).
Table E31. SDiD overall and event time annual outpatient spending ATT estimates (without 2021 integration cohort, PCP only).
Table E32. SDiD overall and event time annual professional spending ATT estimates (PCP only).
Table E33. SDiD overall and event time annual professional spending ATT estimates (without 2021 integration cohort, PCP only).
Table E34. SDiD overall and event time annual pharmaceutical spending ATT estimates (PCP only).
Table E35. SDiD overall and event time annual pharmaceutical spending ATT Estimates (without 2021 integration cohort, PCP only).

## Data Availability

Research data are not shared.
