# Supplementary material for: The Effect of Hospital‐Physician Vertical Integration on Utilization‐Driven Changes in Healthcare Spending for an All‐Payer Population With Multiple Chronic Conditions
Source: Health Serv Res. 2026 Jul 6;61(4):e70144. doi: 10.1111/1475-6773.70144 (PMC13334211; doi:10.1111/1475-6773.70144)
Supplement: Supplementary file 1 — Appendix A. Multiple chronic conditions cohort definition. Table A1. Acute myocardial infarction cohort criteria. Table A2. Alzheimer's and related disorders or senile dementia cohort criteria. Table A3. Atrial fibrillation cohort criteria. Table A4. Chronic kidney disease cohort criteria. Table A5. Chronic obstructive pulmonary disorder (COPD) and asthma cohort criteria. Table A6. Depression cohort criteria. Table A7. Diabetes cohort criteria. Table A8. Heart failure cohort criteria. Table A9. Stroke and transient ischemic attack cohort criteria. Appendix B. Determining hospital‐physician integration. Appendix C. Study cohort flowchart and baseline characteristics. Figure C1. Sample size and cohort flowchart. Table C1. Sample baseline characteristics of MCC patients after IPW, treated by integrated and independent physicians across study period. Table C2. Trends of newly integrated clinicians from 2016 to 2021, by integration cohort. Appendix D. Callaway and Sant'Anna DiD (CSDiD) doubly robust spending results. Figure D1. CSDiD forest plot of overall ATT estimates and 95% CIs for all spending outcomes. Figure D2. CSDiD doubly robust event study plots. Figure D3. CSDiD doubly robust event study results (without 2021 integration cohort). Table D1. CSDiD overall and cohort‐specific total annual spending ATT estimates (unadjusted). Table D2. CSDiD total annual spending event‐time ATT estimates (unadjusted). Table D3. CSDiD overall and cohort‐specific total annual spending ATT estimates. Table D4. CSDiD total annual spending event‐time ATT estimates. Table D5. CSDiD overall and cohort‐specific total annual spending ATT estimates (excluding 2021 integration cohort). Table D6. CSDiD total annual spending event‐time ATT estimates (excluding 2021 integration cohort). Table D7. CSDiD overall and cohort‐specific annual inpatient spending ATT estimates (unadjusted). Table D8. CSDiD annual inpatient spending event‐time ATT estimates (unadjusted). Table D9. CSDiD overall and coh [file HESR-61-e70144-s001.docx]

### Appendix A. Multiple Chronic Conditions Cohort Definition

#### Table A1. Acute Myocardial Infarction Cohort Criteria

| **ICD-10-CM Code** | **ICD-10-CM Code Description** | **Number/Type of Qualifying Claims** | **Diagnoses Used** |
| --- | --- | --- | --- |
| I21.01 | ST elevation (STEMI) myocardial infarction involving left main coronary artery | At least 1 inpatient (IP) claim | 1^st^ or 2^nd^ diagnosis (dx) on claim |
| I21.02 | ST elevation (STEMI) myocardial infarction involving left anterior descending coronary artery | At least 1 IP claim | 1^st^ or 2^nd^ dx on claim |
| I21.09 | ST elevation (STEMI) myocardial infarction involving other coronary artery of anterior wall | At least 1 IP claim | 1^st^ or 2^nd^ dx on claim |
| I21.11 | ST elevation (STEMI) myocardial infarction involving right coronary artery | At least 1 IP claim | 1^st^ or 2^nd^ dx on claim |
| I21.19 | ST elevation (STEMI) myocardial infarction involving other coronary artery of inferior wall | At least 1 IP claim | 1^st^ or 2^nd^ dx on claim |
| I21.21 | ST elevation (STEMI) myocardial infarction involving left circumflex coronary artery | At least 1 IP claim | 1^st^ or 2^nd^ dx on claim |
| I21.29 | ST elevation (STEMI) myocardial infarction involving other sites | At least 1 IP claim | 1^st^ or 2^nd^ dx on claim |
| I21.3 | ST elevation (STEMI) myocardial infarction involving other sites | At least 1 IP claim | 1^st^ or 2^nd^ dx on claim |
| I21.4 | Non-ST elevation (NSTEMI) myocardial infarction | At least 1 IP claim | 1^st^ or 2^nd^ dx on claim |
| I21.9 | Acute myocardial infarction, unspecified | At least 1 IP claim | 1^st^ or 2^nd^ dx on claim |
| I21.A9 | Other myocardial infarction type | At least 1 IP claim | 1^st^ or 2^nd^ dx on claim |
| I22.0 | Subsequent ST elevation (STEMI) myocardial infarction of anterior wall | At least 1 IP claim | 1^st^ or 2^nd^ dx on claim |
| I22.1 | Subsequent ST elevation (STEMI) myocardial infarction of inferior wall | At least 1 IP claim | 1^st^ or 2^nd^ dx on claim |
| I22.2 | Subsequent non-ST elevation (NSTEMI) myocardial infarction | At least 1 IP claim | 1^st^ or 2^nd^ dx on claim |
| I22.8 | Subsequent ST elevation (STEMI) myocardial infarction of other sites | At least 1 IP claim | 1^st^ or 2^nd^ dx on claim |
| I22.9 | Subsequent ST elevation (STEMI) myocardial infarction of unspecified site | At least 1 IP claim | 1^st^ or 2^nd^ dx on claim |

#### Table A2. Alzheimer’s and Related Disorders or Senile Dementia Cohort Criteria

| **ICD-10-CM Code** | **ICD-10-CM Code Description** | **Number/Type of Qualifying Claims** | **Diagnoses Used** |
| --- | --- | --- | --- |
| F01.50 | Vascular dementia without behavioral disturbance | At least 1 IP or 2 outpatient (OP) claims | Any dx on claim |
| F01.51 | Vascular dementia with behavioral disturbance | At least 1 IP or 2 OP claims | Any dx on claim |
| F02.80 | Dementia in other diseases classified elsewhere without behavioral disturbance | At least 1 IP or 2 OP claims | Any dx on claim |
| F02.81 | Dementia in other diseases classified elsewhere with behavioral disturbance | At least 1 IP or 2 OP claims | Any dx on claim |
| F03.90 | Unspecified dementia without behavioral disturbance | At least 1 IP or 2 OP claims | Any dx on claim |
| F03.91 | Unspecified dementia with behavioral disturbance | At least 1 IP or 2 OP claims | Any dx on claim |
| F04 | Amnestic disorder due to known physiological condition | At least 1 IP or 2 OP claims | Any dx on claim |
| F05 | Delirium due to known physiological condition | At least 1 IP or 2 OP claims | Any dx on claim |
| F06.1 | Catatonic disorder due to known physiological condition | At least 1 IP or 2 OP claims | Any dx on claim |
| F06.8 | Other specified mental disorders due to known physiological condition | At least 1 IP or 2 OP claims | Any dx on claim |
| G13.2 | Systemic atrophy primarily affecting the central nervous system in myxedema | At least 1 IP or 2 OP claims | Any dx on claim |
| G13.8 | Systemic atrophy primarily affecting central nervous system in other diseases classified elsewhere | At least 1 IP or 2 OP claims | Any dx on claim |
| G30.0 | Alzheimer's disease with early onset | At least 1 IP or 2 OP claims | Any dx on claim |
| G30.1 | Alzheimer's disease with late onset | At least 1 IP or 2 OP claims | Any dx on claim |
| G30.8 | Other Alzheimer's disease | At least 1 IP or 2 OP claims | Any dx on claim |
| G30.9 | Alzheimer's disease, unspecified | At least 1 IP or 2 OP claims | Any dx on claim |
| G31.1 | Senile degeneration of brain, not elsewhere classified | At least 1 IP or 2 OP claims | Any dx on claim |
| G31.2 | Degeneration of nervous system due to alcohol | At least 1 IP or 2 OP claims | Any dx on claim |
| G31.01 | Pick's disease | At least 1 IP or 2 OP claims | Any dx on claim |
| G31.09 | Other frontotemporal dementia | At least 1 IP or 2 OP claims | Any dx on claim |
| G91.4 | Hydrocephalus in diseases classified elsewhere | At least 1 IP or 2 OP claims | Any dx on claim |
| G94 | Other disorders of brain in diseases classified elsewhere | At least 1 IP or 2 OP claims | Any dx on claim |
| R41.81 | Age-related cognitive decline | At least 1 IP or 2 OP claims | Any dx on claim |
| R54 | Age-related physical debility | At least 1 IP or 2 OP claims | Any dx on claim |

#### Table A3. Atrial Fibrillation Cohort Criteria

| **ICD-10-CM Code** | **ICD-10-CM Code Description** | **Number/Type of Qualifying Claims** | **Diagnoses Used** |
| --- | --- | --- | --- |
| I48.0 | Paroxysmal atrial fibrillation | At least 1 IP or 2 OP claims | 1^st^ or 2^nd^ dx on claim |
| I48.1 | Persistent atrial fibrillation | At least 1 IP or 2 OP claims | 1^st^ or 2^nd^ dx on claim |
| I48.11 | Longstanding persistent atrial fibrillation | At least 1 IP or 2 OP claims | 1^st^ or 2^nd^ dx on claim |
| I48.19 | Other persistent atrial fibrillation | At least 1 IP or 2 OP claims | 1^st^ or 2^nd^ dx on claim |
| I48.2 | Chronic atrial fibrillation | At least 1 IP or 2 OP claims | 1^st^ or 2^nd^ dx on claim |
| I48.20 | Chronic atrial fibrillation, unspecified | At least 1 IP or 2 OP claims | 1^st^ or 2^nd^ dx on claim |
| I48.21 | Permanent atrial fibrillation | At least 1 IP or 2 OP claims | 1^st^ or 2^nd^ dx on claim |
| I48.91 | Unspecified atrial fibrillation | At least 1 IP or 2 OP claims | 1^st^ or 2^nd^ dx on claim |

#### Table A4. Chronic Kidney Disease Cohort Criteria

| **ICD-10-CM Code** | **ICD-10-CM Code Description** | **Number/Type of Qualifying Claims** | **Diagnoses Used** |
| --- | --- | --- | --- |
| A18.11 | Tuberculosis of kidney and ureter | At least 1 IP or 2 OP claims | Any dx on claim |
| A52.75 | Syphilis of kidney and ureter | At least 1 IP or 2 OP claims | Any dx on claim |
| B52.0 | Plasmodium malariae malaria with nephropathy | At least 1 IP or 2 OP claims | Any dx on claim |
| C64.1 | Malignant neoplasm of right kidney, except renal pelvis | At least 1 IP or 2 OP claims | Any dx on claim |
| C64.2 | Malignant neoplasm of left kidney, except renal pelvis | At least 1 IP or 2 OP claims | Any dx on claim |
| C64.9 | Malignant neoplasm of unspecified kidney, except renal pelvis | At least 1 IP or 2 OP claims | Any dx on claim |
| C68.9 | Malignant neoplasm of urinary organ, unspecified | At least 1 IP or 2 OP claims | Any dx on claim |
| D30.00 | Benign neoplasm of unspecified kidney | At least 1 IP or 2 OP claims | Any dx on claim |
| D30.01 | Benign neoplasm of right kidney | At least 1 IP or 2 OP claims | Any dx on claim |
| D30.02 | Benign neoplasm of left kidney | At least 1 IP or 2 OP claims | Any dx on claim |
| D41.00 | Neoplasm of uncertain behavior of unspecified kidney | At least 1 IP or 2 OP claims | Any dx on claim |
| D41.01 | Neoplasm of uncertain behavior of right kidney | At least 1 IP or 2 OP claims | Any dx on claim |
| D41.02 | Neoplasm of uncertain behavior of left kidney | At least 1 IP or 2 OP claims | Any dx on claim |
| D41.10 | Neoplasm of uncertain behavior of unspecified renal pelvis | At least 1 IP or 2 OP claims | Any dx on claim |
| D41.11 | Neoplasm of uncertain behavior of right renal pelvis | At least 1 IP or 2 OP claims | Any dx on claim |
| D41.12 | Neoplasm of uncertain behavior of left renal pelvis | At least 1 IP or 2 OP claims | Any dx on claim |
| D41.20 | Neoplasm of uncertain behavior of unspecified ureter | At least 1 IP or 2 OP claims | Any dx on claim |
| D41.21 | Neoplasm of uncertain behavior of right ureter | At least 1 IP or 2 OP claims | Any dx on claim |
| D41.22 | Neoplasm of uncertain behavior of left ureter | At least 1 IP or 2 OP claims | Any dx on claim |
| D59.3 | Hemolytic-uremic syndrome | At least 1 IP or 2 OP claims | Any dx on claim |
| E08.21 | Diabetes mellitus due to underlying condition with diabetic nephropathy | At least 1 IP or 2 OP claims | Any dx on claim |
| E08.22 | Diabetes mellitus due to underlying condition with diabetic chronic kidney disease | At least 1 IP or 2 OP claims | Any dx on claim |
| E08.29 | Diabetes mellitus due to underlying condition with other diabetic kidney complication | At least 1 IP or 2 OP claims | Any dx on claim |
| E09.21 | Drug or chemical induced diabetes mellitus with diabetic nephropathy | At least 1 IP or 2 OP claims | Any dx on claim |
| E09.22 | Drug or chemical induced diabetes mellitus with diabetic chronic kidney disease | At least 1 IP or 2 OP claims | Any dx on claim |
| E09.29 | Drug or chemical induced diabetes mellitus with other diabetic kidney complication | At least 1 IP or 2 OP claims | Any dx on claim |
| E10.21 | Type 1 diabetes mellitus with diabetic nephropathy | At least 1 IP or 2 OP claims | Any dx on claim |
| E10.22 | Type 1 diabetes mellitus with diabetic chronic kidney disease | At least 1 IP or 2 OP claims | Any dx on claim |
| E10.29 | Type 1 diabetes mellitus with other diabetic kidney complication | At least 1 IP or 2 OP claims | Any dx on claim |
| E11.21 | Type 2 diabetes mellitus with diabetic nephropathy | At least 1 IP or 2 OP claims | Any dx on claim |
| E11.22 | Type 2 diabetes mellitus with diabetic chronic kidney disease | At least 1 IP or 2 OP claims | Any dx on claim |
| E11.29 | Type 2 diabetes mellitus with other diabetic kidney complication | At least 1 IP or 2 OP claims | Any dx on claim |
| E13.21 | Other specified diabetes mellitus with diabetic nephropathy | At least 1 IP or 2 OP claims | Any dx on claim |
| E13.22 | Other specified diabetes mellitus with diabetic chronic kidney disease | At least 1 IP or 2 OP claims | Any dx on claim |
| E13.29 | Other specified diabetes mellitus with other diabetic kidney complication | At least 1 IP or 2 OP claims | Any dx on claim |
| E74.8 | Other specified disorders of carbohydrate metabolism | At least 1 IP or 2 OP claims | Any dx on claim |
| I12.0 | Hypertensive chronic kidney disease with stage 5 chronic kidney disease or end stage renal disease | At least 1 IP or 2 OP claims | Any dx on claim |
| I12.9 | Hypertensive chronic kidney disease with stage 1 through stage 4 chronic kidney disease, or unspecified chronic kidney disease | At least 1 IP or 2 OP claims | Any dx on claim |
| I13.0 | Hypertensive heart and chronic kidney disease with heart failure and stage 1 through stage 4 chronic kidney disease, or unspecified chronic kidney disease | At least 1 IP or 2 OP claims | Any dx on claim |
| I13.10 | Hypertensive heart and chronic kidney disease without heart failure, with stage 1 through stage 4 chronic kidney disease, or unspecified chronic kidney disease | At least 1 IP or 2 OP claims | Any dx on claim |
| I13.11 | Hypertensive heart and chronic kidney disease without heart failure, with stage 5 chronic kidney disease, or end stage renal disease | At least 1 IP or 2 OP claims | Any dx on claim |
| I13.2 | Hypertensive heart and chronic kidney disease with heart failure and with stage 5 chronic kidney disease, or end stage renal disease | At least 1 IP or 2 OP claims | Any dx on claim |
| I70.1 | Atherosclerosis of renal artery | At least 1 IP or 2 OP claims | Any dx on claim |
| I72.2 | Aneurysm of renal artery | At least 1 IP or 2 OP claims | Any dx on claim |
| K76.7 | Hepatorenal syndrome | At least 1 IP or 2 OP claims | Any dx on claim |
| M10.30 | Gout due to renal impairment, unspecified site | At least 1 IP or 2 OP claims | Any dx on claim |
| M10.311 | Gout due to renal impairment, right shoulder | At least 1 IP or 2 OP claims | Any dx on claim |
| M10.312 | Gout due to renal impairment, left shoulder | At least 1 IP or 2 OP claims | Any dx on claim |
| M10.319 | Gout due to renal impairment, unspecified shoulder | At least 1 IP or 2 OP claims | Any dx on claim |
| M10.321 | Gout due to renal impairment, right elbow | At least 1 IP or 2 OP claims | Any dx on claim |
| M10.322 | Gout due to renal impairment, left elbow | At least 1 IP or 2 OP claims | Any dx on claim |
| M10.329 | Gout due to renal impairment, unspecified elbow | At least 1 IP or 2 OP claims | Any dx on claim |
| M10.331 | Gout due to renal impairment, right wrist | At least 1 IP or 2 OP claims | Any dx on claim |
| M10.332 | Gout due to renal impairment, left wrist | At least 1 IP or 2 OP claims | Any dx on claim |
| M10.339 | Gout due to renal impairment, unspecified wrist | At least 1 IP or 2 OP claims | Any dx on claim |
| M10.341 | Gout due to renal impairment, right hand | At least 1 IP or 2 OP claims | Any dx on claim |
| M10.342 | Gout due to renal impairment, left hand | At least 1 IP or 2 OP claims | Any dx on claim |
| M10.349 | Gout due to renal impairment, unspecified hand | At least 1 IP or 2 OP claims | Any dx on claim |
| M10.351 | Gout due to renal impairment, right hip | At least 1 IP or 2 OP claims | Any dx on claim |
| M10.352 | Gout due to renal impairment, left hip | At least 1 IP or 2 OP claims | Any dx on claim |
| M10.359 | Gout due to renal impairment, unspecified hip | At least 1 IP or 2 OP claims | Any dx on claim |
| M10.361 | Gout due to renal impairment, right knee | At least 1 IP or 2 OP claims | Any dx on claim |
| M10.362 | Gout due to renal impairment, left knee | At least 1 IP or 2 OP claims | Any dx on claim |
| M10.369 | Gout due to renal impairment, unspecified knee | At least 1 IP or 2 OP claims | Any dx on claim |
| M10.371 | Gout due to renal impairment, right ankle and foot | At least 1 IP or 2 OP claims | Any dx on claim |
| M10.372 | Gout due to renal impairment, left ankle and foot | At least 1 IP or 2 OP claims | Any dx on claim |
| M10.379 | Gout due to renal impairment, unspecified ankle and foot | At least 1 IP or 2 OP claims | Any dx on claim |
| M10.38 | Gout due to renal impairment, vertebrae | At least 1 IP or 2 OP claims | Any dx on claim |
| M10.39 | Gout due to renal impairment, multiple sites | At least 1 IP or 2 OP claims | Any dx on claim |
| M32.14 | Glomerular disease in systemic lupus erythematosus | At least 1 IP or 2 OP claims | Any dx on claim |
| M32.15 | Tubulo-interstitial nephropathy in systemic lupus erythematosus | At least 1 IP or 2 OP claims | Any dx on claim |
| M35.04 | Sicca syndrome with tubulo-interstitial nephropathy | At least 1 IP or 2 OP claims | Any dx on claim |
| N00.A | Acute nephritic syndrome with C3 glomerulonephritis | At least 1 IP or 2 OP claims | Any dx on claim |
| N00.0 | Acute nephritic syndrome with minor glomerular abnormality | At least 1 IP or 2 OP claims | Any dx on claim |
| N00.1 | Acute nephritic syndrome with focal and segmental glomerular lesions | At least 1 IP or 2 OP claims | Any dx on claim |
| N00.2 | Acute nephritic syndrome with diffuse membranous glomerulonephritis | At least 1 IP or 2 OP claims | Any dx on claim |
| N00.3 | Acute nephritic syndrome with diffuse mesangial proliferative glomerulonephritis | At least 1 IP or 2 OP claims | Any dx on claim |
| N00.4 | Acute nephritic syndrome with diffuse endocapillary proliferative glomerulonephritis | At least 1 IP or 2 OP claims | Any dx on claim |
| N00.5 | Acute nephritic syndrome with diffuse mesangiocapillary glomerulonephritis | At least 1 IP or 2 OP claims | Any dx on claim |
| N00.6 | Acute nephritic syndrome with dense deposit disease | At least 1 IP or 2 OP claims | Any dx on claim |
| N00.7 | Acute nephritic syndrome with diffuse crescentic glomerulonephritis | At least 1 IP or 2 OP claims | Any dx on claim |
| N00.8 | Acute nephritic syndrome with other morphologic changes | At least 1 IP or 2 OP claims | Any dx on claim |
| N00.9 | Acute nephritic syndrome with unspecified morphologic changes | At least 1 IP or 2 OP claims | Any dx on claim |
| N01.A | Rapidly progressive nephritic syndrome with C3 glomerulonephritis | At least 1 IP or 2 OP claims | Any dx on claim |
| N01.0 | Rapidly progressive nephritic syndrome with minor glomerular abnormality | At least 1 IP or 2 OP claims | Any dx on claim |
| N01.1 | Rapidly progressive nephritic syndrome with focal and segmental glomerular lesions | At least 1 IP or 2 OP claims | Any dx on claim |
| N01.2 | Rapidly progressive nephritic syndrome with diffuse membranous glomerulonephritis | At least 1 IP or 2 OP claims | Any dx on claim |
| N01.3 | Rapidly progressive nephritic syndrome with diffuse mesangial proliferative glomerulonephritis | At least 1 IP or 2 OP claims | Any dx on claim |
| N01.4 | Rapidly progressive nephritic syndrome with diffuse endocapillary proliferative glomerulonephritis | At least 1 IP or 2 OP claims | Any dx on claim |
| N01.5 | Rapidly progressive nephritic syndrome with diffuse mesangiocapillary glomerulonephritis | At least 1 IP or 2 OP claims | Any dx on claim |
| N01.6 | Rapidly progressive nephritic syndrome with dense deposit disease | At least 1 IP or 2 OP claims | Any dx on claim |
| N01.7 | Rapidly progressive nephritic syndrome with diffuse crescentic glomerulonephritis | At least 1 IP or 2 OP claims | Any dx on claim |
| N01.8 | Rapidly progressive nephritic syndrome with other morphologic changes | At least 1 IP or 2 OP claims | Any dx on claim |
| N01.9 | Rapidly progressive nephritic syndrome with unspecified morphologic changes | At least 1 IP or 2 OP claims | Any dx on claim |
| N02.A | Recurrent and persistent hematuria with C3 glomerulonephritis | At least 1 IP or 2 OP claims | Any dx on claim |
| N02.0 | Recurrent and persistent hematuria with minor glomerular abnormality | At least 1 IP or 2 OP claims | Any dx on claim |
| N02.1 | Recurrent and persistent hematuria with focal and segmental glomerular lesions | At least 1 IP or 2 OP claims | Any dx on claim |
| N02.2 | Recurrent and persistent hematuria with diffuse membranous glomerulonephritis | At least 1 IP or 2 OP claims | Any dx on claim |
| N02.3 | Recurrent and persistent hematuria with diffuse mesangial proliferative glomerulonephritis | At least 1 IP or 2 OP claims | Any dx on claim |
| N02.4 | Recurrent and persistent hematuria with diffuse endocapillary proliferative glomerulonephritis | At least 1 IP or 2 OP claims | Any dx on claim |
| N02.5 | Recurrent and persistent hematuria with diffuse mesangiocapillary glomerulonephritis | At least 1 IP or 2 OP claims | Any dx on claim |
| N02.6 | Recurrent and persistent hematuria with dense deposit disease | At least 1 IP or 2 OP claims | Any dx on claim |
| N02.7 | Recurrent and persistent hematuria with diffuse crescentic glomerulonephritis | At least 1 IP or 2 OP claims | Any dx on claim |
| N02.8 | Recurrent and persistent hematuria with other morphologic changes | At least 1 IP or 2 OP claims | Any dx on claim |
| N02.9 | Recurrent and persistent hematuria with unspecified morphologic changes | At least 1 IP or 2 OP claims | Any dx on claim |
| N03.A | Chronic nephritic syndrome with C3 glomerulonephritis | At least 1 IP or 2 OP claims | Any dx on claim |
| N03.0 | Chronic nephritic syndrome with minor glomerular abnormality | At least 1 IP or 2 OP claims | Any dx on claim |
| N03.1 | Chronic nephritic syndrome with focal and segmental glomerular lesions | At least 1 IP or 2 OP claims | Any dx on claim |
| N03.2 | Chronic nephritic syndrome with diffuse membranous glomerulonephritis | At least 1 IP or 2 OP claims | Any dx on claim |
| N03.3 | Chronic nephritic syndrome with diffuse mesangial proliferative glomerulonephritis | At least 1 IP or 2 OP claims | Any dx on claim |
| N03.4 | Chronic nephritic syndrome with diffuse endocapillary proliferative glomerulonephritis | At least 1 IP or 2 OP claims | Any dx on claim |
| N03.5 | Chronic nephritic syndrome with diffuse mesangiocapillary glomerulonephritis | At least 1 IP or 2 OP claims | Any dx on claim |
| N03.6 | Chronic nephritic syndrome with dense deposit disease | At least 1 IP or 2 OP claims | Any dx on claim |
| N03.7 | Chronic nephritic syndrome with diffuse crescentic glomerulonephritis | At least 1 IP or 2 OP claims | Any dx on claim |
| N03.8 | Chronic nephritic syndrome with other morphologic changes | At least 1 IP or 2 OP claims | Any dx on claim |
| N03.9 | Chronic nephritic syndrome with unspecified morphologic changes | At least 1 IP or 2 OP claims | Any dx on claim |
| N04.A | Nephrotic syndrome with C3 glomerulonephritis | At least 1 IP or 2 OP claims | Any dx on claim |
| N04.0 | Nephrotic syndrome with minor glomerular abnormality | At least 1 IP or 2 OP claims | Any dx on claim |
| N04.1 | Nephrotic syndrome with focal and segmental glomerular lesions | At least 1 IP or 2 OP claims | Any dx on claim |
| N04.2 | Nephrotic syndrome with diffuse membranous glomerulonephritis | At least 1 IP or 2 OP claims | Any dx on claim |
| N04.3 | Nephrotic syndrome with diffuse mesangial proliferative glomerulonephritis | At least 1 IP or 2 OP claims | Any dx on claim |
| N04.4 | Nephrotic syndrome with diffuse endocapillary proliferative glomerulonephritis | At least 1 IP or 2 OP claims | Any dx on claim |
| N04.5 | Nephrotic syndrome with diffuse mesangiocapillary glomerulonephritis | At least 1 IP or 2 OP claims | Any dx on claim |
| N04.6 | Nephrotic syndrome with dense deposit disease | At least 1 IP or 2 OP claims | Any dx on claim |
| N04.7 | Nephrotic syndrome with diffuse crescentic glomerulonephritis | At least 1 IP or 2 OP claims | Any dx on claim |
| N04.8 | Nephrotic syndrome with other morphologic changes | At least 1 IP or 2 OP claims | Any dx on claim |
| N04.9 | Nephrotic syndrome with unspecified morphologic changes | At least 1 IP or 2 OP claims | Any dx on claim |
| N05.A | Unspecified nephritic syndrome with C3 glomerulonephritis | At least 1 IP or 2 OP claims | Any dx on claim |
| N05.0 | Unspecified nephritic syndrome with minor glomerular abnormality | At least 1 IP or 2 OP claims | Any dx on claim |
| N05.1 | Unspecified nephritic syndrome with focal and segmental glomerular lesions | At least 1 IP or 2 OP claims | Any dx on claim |
| N05.2 | Unspecified nephritic syndrome with diffuse membranous glomerulonephritis | At least 1 IP or 2 OP claims | Any dx on claim |
| N05.3 | Unspecified nephritic syndrome with diffuse mesangial proliferative glomerulonephritis | At least 1 IP or 2 OP claims | Any dx on claim |
| N05.4 | Unspecified nephritic syndrome with diffuse endocapillary proliferative glomerulonephritis | At least 1 IP or 2 OP claims | Any dx on claim |
| N05.5 | Unspecified nephritic syndrome with diffuse mesangiocapillary glomerulonephritis | At least 1 IP or 2 OP claims | Any dx on claim |
| N05.6 | Unspecified nephritic syndrome with dense deposit disease | At least 1 IP or 2 OP claims | Any dx on claim |
| N05.7 | Unspecified nephritic syndrome with diffuse crescentic glomerulonephritis | At least 1 IP or 2 OP claims | Any dx on claim |
| N05.8 | Unspecified nephritic syndrome with other morphologic changes | At least 1 IP or 2 OP claims | Any dx on claim |
| N05.9 | Unspecified nephritic syndrome with unspecified morphologic changes | At least 1 IP or 2 OP claims | Any dx on claim |
| N06.A | Isolated proteinuria with C3 glomerulonephritis | At least 1 IP or 2 OP claims | Any dx on claim |
| N06.0 | Isolated proteinuria with minor glomerular abnormality | At least 1 IP or 2 OP claims | Any dx on claim |
| N06.1 | Isolated proteinuria with focal and segmental glomerular lesions | At least 1 IP or 2 OP claims | Any dx on claim |
| N06.2 | Isolated proteinuria with diffuse membranous glomerulonephritis | At least 1 IP or 2 OP claims | Any dx on claim |
| N06.3 | Isolated proteinuria with diffuse mesangial proliferative glomerulonephritis | At least 1 IP or 2 OP claims | Any dx on claim |
| N06.4 | Isolated proteinuria with diffuse endocapillary proliferative glomerulonephritis | At least 1 IP or 2 OP claims | Any dx on claim |
| N06.5 | Isolated proteinuria with diffuse mesangiocapillary glomerulonephritis | At least 1 IP or 2 OP claims | Any dx on claim |
| N06.6 | Isolated proteinuria with dense deposit disease | At least 1 IP or 2 OP claims | Any dx on claim |
| N06.7 | Isolated proteinuria with diffuse crescentic glomerulonephritis | At least 1 IP or 2 OP claims | Any dx on claim |
| N06.8 | Isolated proteinuria with other morphologic lesion | At least 1 IP or 2 OP claims | Any dx on claim |
| N06.9 | Isolated proteinuria with unspecified morphologic lesion | At least 1 IP or 2 OP claims | Any dx on claim |
| N07.A | Hereditary nephropathy, not elsewhere classified with C3 glomerulonephritis | At least 1 IP or 2 OP claims | Any dx on claim |
| N07.0 | Hereditary nephropathy, not elsewhere classified with minor glomerular abnormality | At least 1 IP or 2 OP claims | Any dx on claim |
| N07.1 | Hereditary nephropathy, not elsewhere classified with focal and segmental glomerular lesions | At least 1 IP or 2 OP claims | Any dx on claim |
| N07.2 | Hereditary nephropathy, not elsewhere classified with diffuse membranous glomerulonephritis | At least 1 IP or 2 OP claims | Any dx on claim |
| N07.3 | Hereditary nephropathy, not elsewhere classified with diffuse mesangial proliferative glomerulonephritis | At least 1 IP or 2 OP claims | Any dx on claim |
| N07.4 | Hereditary nephropathy, not elsewhere classified with diffuse endocapillary proliferative glomerulonephritis | At least 1 IP or 2 OP claims | Any dx on claim |
| N07.5 | Hereditary nephropathy, not elsewhere classified with diffuse mesangiocapillary glomerulonephritis | At least 1 IP or 2 OP claims | Any dx on claim |
| N07.6 | Hereditary nephropathy, not elsewhere classified with dense deposit disease | At least 1 IP or 2 OP claims | Any dx on claim |
| N07.7 | Hereditary nephropathy, not elsewhere classified with diffuse crescentic glomerulonephritis | At least 1 IP or 2 OP claims | Any dx on claim |
| N07.8 | Hereditary nephropathy, not elsewhere classified with other morphologic lesions | At least 1 IP or 2 OP claims | Any dx on claim |
| N07.9 | Hereditary nephropathy, not elsewhere classified with unspecified morphologic lesions | At least 1 IP or 2 OP claims | Any dx on claim |
| N08 | Glomerular disorders in diseases classified elsewhere | At least 1 IP or 2 OP claims | Any dx on claim |
| N13.0 | Hydronephrosis with ureteropelvic junction obstruction | At least 1 IP or 2 OP claims | Any dx on claim |
| N13.1 | Hydronephrosis with ureteral stricture, not elsewhere classified | At least 1 IP or 2 OP claims | Any dx on claim |
| N13.2 | Hydronephrosis with renal and ureteral calculous obstruction | At least 1 IP or 2 OP claims | Any dx on claim |
| N13.30 | Unspecified hydronephrosis | At least 1 IP or 2 OP claims | Any dx on claim |
| N13.39 | Other hydronephrosis | At least 1 IP or 2 OP claims | Any dx on claim |
| N14.0 | Analgesic nephropathy | At least 1 IP or 2 OP claims | Any dx on claim |
| N14.1 | Nephropathy induced by other drugs, medicaments and biological substances | At least 1 IP or 2 OP claims | Any dx on claim |
| N14.2 | Nephropathy induced by unspecified drug, medicament or biological substance | At least 1 IP or 2 OP claims | Any dx on claim |
| N14.3 | Nephropathy induced by heavy metals | At least 1 IP or 2 OP claims | Any dx on claim |
| N14.4 | Toxic nephropathy, not elsewhere classified | At least 1 IP or 2 OP claims | Any dx on claim |
| N15.0 | Balkan nephropathy | At least 1 IP or 2 OP claims | Any dx on claim |
| N15.8 | Other specified renal tubulo-interstitial diseases | At least 1 IP or 2 OP claims | Any dx on claim |
| N15.9 | Renal tubulo-interstitial disease, unspecified | At least 1 IP or 2 OP claims | Any dx on claim |
| N16 | Renal tubulo-interstitial disorders in diseases classified elsewhere | At least 1 IP or 2 OP claims | Any dx on claim |
| N17.0 | Acute kidney failure with tubular necrosis | At least 1 IP or 2 OP claims | Any dx on claim |
| N17.1 | Acute kidney failure with acute cortical necrosis | At least 1 IP or 2 OP claims | Any dx on claim |
| N17.2 | Acute kidney failure with medullary necrosis | At least 1 IP or 2 OP claims | Any dx on claim |
| N17.8 | Other acute kidney failure | At least 1 IP or 2 OP claims | Any dx on claim |
| N17.9 | Acute kidney failure, unspecified | At least 1 IP or 2 OP claims | Any dx on claim |
| N18.1 | Chronic kidney disease, stage 1 | At least 1 IP or 2 OP claims | Any dx on claim |
| N18.2 | Chronic kidney disease, stage 2 (mild) | At least 1 IP or 2 OP claims | Any dx on claim |
| N18.3 | Chronic kidney disease, stage 3 (moderate) | At least 1 IP or 2 OP claims | Any dx on claim |
| N18.30 | Chronic kidney disease, stage 3 unspecified | At least 1 IP or 2 OP claims | Any dx on claim |
| N18.31 | Chronic kidney disease, stage 3a | At least 1 IP or 2 OP claims | Any dx on claim |
| N18.32 | Chronic kidney disease, stage 3b | At least 1 IP or 2 OP claims | Any dx on claim |
| N18.4 | Chronic kidney disease, stage 4 (severe) | At least 1 IP or 2 OP claims | Any dx on claim |
| N18.5 | Chronic kidney disease, stage 5 | At least 1 IP or 2 OP claims | Any dx on claim |
| N18.6 | End stage renal disease | At least 1 IP or 2 OP claims | Any dx on claim |
| N18.9 | Chronic kidney disease, unspecified | At least 1 IP or 2 OP claims | Any dx on claim |
| N19 | Unspecified kidney failure | At least 1 IP or 2 OP claims | Any dx on claim |
| N25.0 | Renal osteodystrophy | At least 1 IP or 2 OP claims | Any dx on claim |
| N25.1 | Nephrogenic diabetes insipidus | At least 1 IP or 2 OP claims | Any dx on claim |
| N25.81 | Secondary hyperparathyroidism of renal origin | At least 1 IP or 2 OP claims | Any dx on claim |
| N25.89 | Other disorders resulting from impaired renal tubular function | At least 1 IP or 2 OP claims | Any dx on claim |
| N25.9 | Disorder resulting from impaired renal tubular function, unspecified | At least 1 IP or 2 OP claims | Any dx on claim |
| N26.1 | Atrophy of kidney (terminal) | At least 1 IP or 2 OP claims | Any dx on claim |
| N26.9 | Renal sclerosis, unspecified | At least 1 IP or 2 OP claims | Any dx on claim |
| Q61.02 | Congenital multiple renal cysts | At least 1 IP or 2 OP claims | Any dx on claim |
| Q61.11 | Cystic dilatation of collecting ducts | At least 1 IP or 2 OP claims | Any dx on claim |
| Q61.19 | Other polycystic kidney, infantile type | At least 1 IP or 2 OP claims | Any dx on claim |
| Q61.2 | Polycystic kidney, adult type | At least 1 IP or 2 OP claims | Any dx on claim |
| Q61.3 | Polycystic kidney, unspecified | At least 1 IP or 2 OP claims | Any dx on claim |
| Q61.4 | Renal dysplasia | At least 1 IP or 2 OP claims | Any dx on claim |
| Q61.5 | Medullary cystic kidney | At least 1 IP or 2 OP claims | Any dx on claim |
| Q61.8 | Other cystic kidney diseases | At least 1 IP or 2 OP claims | Any dx on claim |
| Q62.0 | Congenital hydronephrosis | At least 1 IP or 2 OP claims | Any dx on claim |
| Q62.2 | Congenital megaureter | At least 1 IP or 2 OP claims | Any dx on claim |
| Q62.10 | Congenital occlusion of ureter, unspecified | At least 1 IP or 2 OP claims | Any dx on claim |
| Q62.11 | Congenital occlusion of ureteropelvic junction | At least 1 IP or 2 OP claims | Any dx on claim |
| Q62.12 | Congenital occlusion of ureterovesical orifice | At least 1 IP or 2 OP claims | Any dx on claim |
| Q62.31 | Congenital ureterocele, orthotopic | At least 1 IP or 2 OP claims | Any dx on claim |
| Q62.32 | Cecoureterocele | At least 1 IP or 2 OP claims | Any dx on claim |
| Q62.39 | Other obstructive defects of renal pelvis and ureter | At least 1 IP or 2 OP claims | Any dx on claim |
| R94.4 | Abnormal results of kidney function studies | At least 1 IP or 2 OP claims | Any dx on claim |

#### Table A5. Chronic Obstructive Pulmonary Disorder (COPD) and Asthma Cohort Criteria

| **ICD-10-CM Code** | **ICD-10-CM Code Description** | **Number/Type of Qualifying Claims** | **Diagnoses Used** |
| --- | --- | --- | --- |
| J40 | Bronchitis, not specified as acute or chronic | At least 1 IP or 2 OP claims | Any dx on claim |
| J41.0 | Simple chronic bronchitis | At least 1 IP or 2 OP claims | Any dx on claim |
| J41.1 | Mucopurulent chronic bronchitis | At least 1 IP or 2 OP claims | Any dx on claim |
| J41.8 | Mixed simple and mucopurulent chronic bronchitis | At least 1 IP or 2 OP claims | Any dx on claim |
| J42 | Unspecified chronic bronchitis | At least 1 IP or 2 OP claims | Any dx on claim |
| J43.0 | Unilateral pulmonary emphysema [MacLeod's syndrome] | At least 1 IP or 2 OP claims | Any dx on claim |
| J43.1 | Panlobular emphysema | At least 1 IP or 2 OP claims | Any dx on claim |
| J43.2 | Centrilobular emphysema | At least 1 IP or 2 OP claims | Any dx on claim |
| J43.8 | Other emphysema | At least 1 IP or 2 OP claims | Any dx on claim |
| J43.9 | Emphysema, unspecified | At least 1 IP or 2 OP claims | Any dx on claim |
| J44.0 | Chronic obstructive pulmonary disease with acute lower respiratory infection | At least 1 IP or 2 OP claims | Any dx on claim |
| J44.1 | Chronic obstructive pulmonary disease with (acute) exacerbation | At least 1 IP or 2 OP claims | Any dx on claim |
| J44.9 | Chronic obstructive pulmonary disease, unspecified | At least 1 IP or 2 OP claims | Any dx on claim |
| J47.0 | Bronchiectasis with acute lower respiratory infection | At least 1 IP or 2 OP claims | Any dx on claim |
| J47.1 | Bronchiectasis with (acute) exacerbation | At least 1 IP or 2 OP claims | Any dx on claim |
| J47.9 | Bronchiectasis, uncomplicated | At least 1 IP or 2 OP claims | Any dx on claim |
| J45.20 | Mild intermittent asthma, uncomplicated | At least 1 IP or 2 OP claims | Any dx on claim |
| J45.21 | Mild intermittent asthma with (acute) exacerbation | At least 1 IP or 2 OP claims | Any dx on claim |
| J45.22 | Mild intermittent asthma with status asthmaticus | At least 1 IP or 2 OP claims | Any dx on claim |
| J45.30 | Mild persistent asthma, uncomplicated | At least 1 IP or 2 OP claims | Any dx on claim |
| J45.31 | Mild persistent asthma with (acute) exacerbation | At least 1 IP or 2 OP claims | Any dx on claim |
| J45.32 | Mild persistent asthma with status asthmaticus | At least 1 IP or 2 OP claims | Any dx on claim |
| J45.40 | Moderate persistent asthma, uncomplicated | At least 1 IP or 2 OP claims | Any dx on claim |
| J45.41 | Moderate persistent asthma with (acute) exacerbation | At least 1 IP or 2 OP claims | Any dx on claim |
| J45.42 | Moderate persistent asthma with status asthmaticus | At least 1 IP or 2 OP claims | Any dx on claim |
| J45.50 | Severe persistent asthma, uncomplicated | At least 1 IP or 2 OP claims | Any dx on claim |
| J45.51 | Severe persistent asthma with (acute) exacerbation | At least 1 IP or 2 OP claims | Any dx on claim |
| J45.52 | Severe persistent asthma with status asthmaticus | At least 1 IP or 2 OP claims | Any dx on claim |
| J45.901 | Unspecified asthma with (acute) exacerbation | At least 1 IP or 2 OP claims | Any dx on claim |
| J45.902 | Unspecified asthma with status asthmaticus | At least 1 IP or 2 OP claims | Any dx on claim |
| J45.909 | Unspecified asthma, uncomplicated | At least 1 IP or 2 OP claims | Any dx on claim |
| J45.990 | Exercise induced bronchospasm | At least 1 IP or 2 OP claims | Any dx on claim |
| J45.991 | Cough variant asthma | At least 1 IP or 2 OP claims | Any dx on claim |
| J45.998 | Other asthma | At least 1 IP or 2 OP claims | Any dx on claim |

#### Table A6. Depression Cohort Criteria

| **ICD-10-CM Code** | **ICD-10-CM Code Description** | **Number/Type of Qualifying Claims** | **Diagnoses Used** |
| --- | --- | --- | --- |
| F31.30 | Bipolar disorder, current episode depressed, mild or moderate severity, unspecified | At least 1 IP or 2 OP claims | Any dx on claim |
| F31.31 | Bipolar disorder, current episode depressed, mild | At least 1 IP or 2 OP claims | Any dx on claim |
| F31.32 | Bipolar disorder, current episode depressed, moderate | At least 1 IP or 2 OP claims | Any dx on claim |
| F31.4 | Bipolar disorder, current episode depressed, severe, without psychotic features | At least 1 IP or 2 OP claims | Any dx on claim |
| F31.5 | Bipolar disorder, current episode depressed, severe, with psychotic features | At least 1 IP or 2 OP claims | Any dx on claim |
| F31.60 | Bipolar disorder, current episode mixed, unspecified | At least 1 IP or 2 OP claims | Any dx on claim |
| F31.61 | Bipolar disorder, current episode mixed, mild | At least 1 IP or 2 OP claims | Any dx on claim |
| F31.62 | Bipolar disorder, current episode mixed, moderate | At least 1 IP or 2 OP claims | Any dx on claim |
| F31.63 | Bipolar disorder, current episode mixed, severe, without psychotic features | At least 1 IP or 2 OP claims | Any dx on claim |
| F31.64 | Bipolar disorder, current episode mixed, severe, with psychotic features | At least 1 IP or 2 OP claims | Any dx on claim |
| F31.75 | Bipolar disorder, in partial remission, most recent episode depressed | At least 1 IP or 2 OP claims | Any dx on claim |
| F31.76 | Bipolar disorder, in full remission, most recent episode depressed | At least 1 IP or 2 OP claims | Any dx on claim |
| F31.77 | Bipolar disorder, in partial remission, most recent episode mixed | At least 1 IP or 2 OP claims | Any dx on claim |
| F31.78 | Bipolar disorder, in full remission, most recent episode mixed | At least 1 IP or 2 OP claims | Any dx on claim |
| F31.81 | Bipolar II disorder | At least 1 IP or 2 OP claims | Any dx on claim |
| F32.0 | Major depressive disorder, single episode, mild | At least 1 IP or 2 OP claims | Any dx on claim |
| F32.1 | Major depressive disorder, single episode, moderate | At least 1 IP or 2 OP claims | Any dx on claim |
| F32.2 | Major depressive disorder, single episode, severe without psychotic features | At least 1 IP or 2 OP claims | Any dx on claim |
| F32.3 | Major depressive disorder, single episode, severe with psychotic features | At least 1 IP or 2 OP claims | Any dx on claim |
| F32.4 | Major depressive disorder, single episode, in partial remission | At least 1 IP or 2 OP claims | Any dx on claim |
| F32.5 | Major depressive disorder, single episode, in full remission | At least 1 IP or 2 OP claims | Any dx on claim |
| F32.9 | Major depressive disorder, single episode, unspecified | At least 1 IP or 2 OP claims | Any dx on claim |
| F33.0 | Major depressive disorder, recurrent, mild | At least 1 IP or 2 OP claims | Any dx on claim |
| F33.1 | Major depressive disorder, recurrent, moderate | At least 1 IP or 2 OP claims | Any dx on claim |
| F33.2 | Major depressive disorder, recurrent severe without psychotic features | At least 1 IP or 2 OP claims | Any dx on claim |
| F33.3 | Major depressive disorder, recurrent, severe with psychotic symptoms | At least 1 IP or 2 OP claims | Any dx on claim |
| F33.40 | Major depressive disorder, recurrent, in remission, unspecified | At least 1 IP or 2 OP claims | Any dx on claim |
| F33.41 | Major depressive disorder, recurrent, in partial remission | At least 1 IP or 2 OP claims | Any dx on claim |
| F33.42 | Major depressive disorder, recurrent, in full remission | At least 1 IP or 2 OP claims | Any dx on claim |
| F33.8 | Other recurrent depressive disorders | At least 1 IP or 2 OP claims | Any dx on claim |
| F33.9 | Major depressive disorder, recurrent, unspecified | At least 1 IP or 2 OP claims | Any dx on claim |
| F34.1 | Dysthymic disorder | At least 1 IP or 2 OP claims | Any dx on claim |
| F43.21 | Adjustment disorder with depressed mood | At least 1 IP or 2 OP claims | Any dx on claim |
| F43.23 | Adjustment disorder with mixed anxiety and depressed mood | At least 1 IP or 2 OP claims | Any dx on claim |

#### Table A7. Diabetes Cohort Criteria

| **ICD-10-CM Code** | **ICD-10-CM Code Description** | **Number/Type of Qualifying Claims** | **Diagnoses Used** |
| --- | --- | --- | --- |
| E10.10 | Type 1 diabetes mellitus with ketoacidosis without coma | At least 1 IP or 2 OP claims | Any dx on claim |
| E10.11 | Type 1 diabetes mellitus with ketoacidosis with coma | At least 1 IP or 2 OP claims | Any dx on claim |
| E10.21 | Type 1 diabetes mellitus with diabetic nephropathy | At least 1 IP or 2 OP claims | Any dx on claim |
| E10.22 | Type 1 diabetes mellitus with diabetic chronic kidney disease | At least 1 IP or 2 OP claims | Any dx on claim |
| E10.29 | Type 1 diabetes mellitus with other diabetic kidney complication | At least 1 IP or 2 OP claims | Any dx on claim |
| E10.311 | Type 1 diabetes mellitus with unspecified diabetic retinopathy with macular edema | At least 1 IP or 2 OP claims | Any dx on claim |
| E10.319 | Type 1 diabetes mellitus with unspecified diabetic retinopathy without macular edema | At least 1 IP or 2 OP claims | Any dx on claim |
| E10.321 | Type 1 diabetes mellitus with mild nonproliferative diabetic retinopathy with macular edema | At least 1 IP or 2 OP claims | Any dx on claim |
| E10.3211 | Type 1 diabetes mellitus with mild nonproliferative diabetic retinopathy with macular edema, right eye | At least 1 IP or 2 OP claims | Any dx on claim |
| E10.3212 | Type 1 diabetes mellitus with mild nonproliferative diabetic retinopathy with macular edema, left eye | At least 1 IP or 2 OP claims | Any dx on claim |
| E10.3213 | Type 1 diabetes mellitus with mild nonproliferative diabetic retinopathy with macular edema, bilateral | At least 1 IP or 2 OP claims | Any dx on claim |
| E10.3219 | Type 1 diabetes mellitus with mild nonproliferative diabetic retinopathy with macular edema, unspecified eye | At least 1 IP or 2 OP claims | Any dx on claim |
| E10.329 | Type 1 diabetes mellitus with mild nonproliferative diabetic retinopathy without macular edema | At least 1 IP or 2 OP claims | Any dx on claim |
| E10.3291 | Type 1 diabetes mellitus with mild nonproliferative diabetic retinopathy without macular edema, right eye | At least 1 IP or 2 OP claims | Any dx on claim |
| E10.3292 | Type 1 diabetes mellitus with mild nonproliferative diabetic retinopathy without macular edema, left eye | At least 1 IP or 2 OP claims | Any dx on claim |
| E10.3293 | Type 1 diabetes mellitus with mild nonproliferative diabetic retinopathy without macular edema, bilateral | At least 1 IP or 2 OP claims | Any dx on claim |
| E10.3299 | Type 1 diabetes mellitus with mild nonproliferative diabetic retinopathy without macular edema, unspecified eye | At least 1 IP or 2 OP claims | Any dx on claim |
| E10.331 | Type 1 diabetes mellitus with moderate nonproliferative diabetic retinopathy with macular edema | At least 1 IP or 2 OP claims | Any dx on claim |
| E10.3311 | Type 1 diabetes mellitus with moderate nonproliferative diabetic retinopathy with macular edema, right eye | At least 1 IP or 2 OP claims | Any dx on claim |
| E10.3312 | Type 1 diabetes mellitus with moderate nonproliferative diabetic retinopathy with macular edema, left eye | At least 1 IP or 2 OP claims | Any dx on claim |
| E10.3313 | Type 1 diabetes mellitus with moderate nonproliferative diabetic retinopathy with macular edema, bilateral | At least 1 IP or 2 OP claims | Any dx on claim |
| E10.3319 | Type 1 diabetes mellitus with moderate nonproliferative diabetic retinopathy with macular edema, unspecified eye | At least 1 IP or 2 OP claims | Any dx on claim |
| E10.339 | Type 1 diabetes mellitus with moderate nonproliferative diabetic retinopathy without macular edema | At least 1 IP or 2 OP claims | Any dx on claim |
| E10.3391 | Type 1 diabetes mellitus with moderate nonproliferative diabetic retinopathy without macular edema, right eye | At least 1 IP or 2 OP claims | Any dx on claim |
| E10.3392 | Type 1 diabetes mellitus with moderate nonproliferative diabetic retinopathy without macular edema, left eye | At least 1 IP or 2 OP claims | Any dx on claim |
| E10.3393 | Type 1 diabetes mellitus with moderate nonproliferative diabetic retinopathy without macular edema, bilateral | At least 1 IP or 2 OP claims | Any dx on claim |
| E10.3399 | Type 1 diabetes mellitus with moderate nonproliferative diabetic retinopathy without macular edema, unspecified eye | At least 1 IP or 2 OP claims | Any dx on claim |
| E10.341 | Type 1 diabetes mellitus with severe nonproliferative diabetic retinopathy with macular edema | At least 1 IP or 2 OP claims | Any dx on claim |
| E10.3411 | Type 1 diabetes mellitus with severe nonproliferative diabetic retinopathy with macular edema, right eye | At least 1 IP or 2 OP claims | Any dx on claim |
| E10.3412 | Type 1 diabetes mellitus with severe nonproliferative diabetic retinopathy with macular edema, left eye | At least 1 IP or 2 OP claims | Any dx on claim |
| E10.3413 | Type 1 diabetes mellitus with severe nonproliferative diabetic retinopathy with macular edema, bilateral | At least 1 IP or 2 OP claims | Any dx on claim |
| E10.3419 | Type 1 diabetes mellitus with severe nonproliferative diabetic retinopathy with macular edema, unspecified eye | At least 1 IP or 2 OP claims | Any dx on claim |
| E10.349 | Type 1 diabetes mellitus with severe nonproliferative diabetic retinopathy without macular edema | At least 1 IP or 2 OP claims | Any dx on claim |
| E10.3491 | Type 1 diabetes mellitus with severe nonproliferative diabetic retinopathy without macular edema, right eye | At least 1 IP or 2 OP claims | Any dx on claim |
| E10.3492 | Type 1 diabetes mellitus with severe nonproliferative diabetic retinopathy without macular edema, left eye | At least 1 IP or 2 OP claims | Any dx on claim |
| E10.3493 | Type 1 diabetes mellitus with severe nonproliferative diabetic retinopathy without macular edema, bilateral | At least 1 IP or 2 OP claims | Any dx on claim |
| E10.3499 | Type 1 diabetes mellitus with severe nonproliferative diabetic retinopathy without macular edema, unspecified eye | At least 1 IP or 2 OP claims | Any dx on claim |
| E10.351 | Type 1 diabetes mellitus with proliferative diabetic retinopathy with macular edema | At least 1 IP or 2 OP claims | Any dx on claim |
| E10.3511 | Type 1 diabetes mellitus with proliferative diabetic retinopathy with macular edema, right eye | At least 1 IP or 2 OP claims | Any dx on claim |
| E10.3512 | Type 1 diabetes mellitus with proliferative diabetic retinopathy with macular edema, left eye | At least 1 IP or 2 OP claims | Any dx on claim |
| E10.3513 | Type 1 diabetes mellitus with proliferative diabetic retinopathy with macular edema, bilateral | At least 1 IP or 2 OP claims | Any dx on claim |
| E10.3519 | Type 1 diabetes mellitus with proliferative diabetic retinopathy with macular edema, unspecified eye | At least 1 IP or 2 OP claims | Any dx on claim |
| E10.3521 | Type 1 diabetes mellitus with proliferative diabetic retinopathy with traction retinal detachment involving the macula, right eye | At least 1 IP or 2 OP claims | Any dx on claim |
| E10.3522 | Type 1 diabetes mellitus with proliferative diabetic retinopathy with traction retinal detachment involving the macula, left eye | At least 1 IP or 2 OP claims | Any dx on claim |
| E10.3523 | Type 1 diabetes mellitus with proliferative diabetic retinopathy with traction retinal detachment involving the macula, bilateral | At least 1 IP or 2 OP claims | Any dx on claim |
| E10.3529 | Type 1 diabetes mellitus with proliferative diabetic retinopathy with traction retinal detachment involving the macula, unspecified eye | At least 1 IP or 2 OP claims | Any dx on claim |
| E10.3531 | Type 1 diabetes mellitus with proliferative diabetic retinopathy with traction retinal detachment not involving the macula, right eye | At least 1 IP or 2 OP claims | Any dx on claim |
| E10.3532 | Type 1 diabetes mellitus with proliferative diabetic retinopathy with traction retinal detachment not involving the macula, left eye | At least 1 IP or 2 OP claims | Any dx on claim |
| E10.3533 | Type 1 diabetes mellitus with proliferative diabetic retinopathy with traction retinal detachment not involving the macula, bilateral | At least 1 IP or 2 OP claims | Any dx on claim |
| E10.3539 | Type 1 diabetes mellitus with proliferative diabetic retinopathy with traction retinal detachment not involving the macula, unspecified eye | At least 1 IP or 2 OP claims | Any dx on claim |
| E10.3541 | Type 1 diabetes mellitus with proliferative diabetic retinopathy with combined traction retinal detachment and rhegmatogenous retinal detachment, right eye | At least 1 IP or 2 OP claims | Any dx on claim |
| E10.3542 | Type 1 diabetes mellitus with proliferative diabetic retinopathy with combined traction retinal detachment and rhegmatogenous retinal detachment, left eye | At least 1 IP or 2 OP claims | Any dx on claim |
| E10.3543 | Type 1 diabetes mellitus with proliferative diabetic retinopathy with combined traction retinal detachment and rhegmatogenous retinal detachment, bilateral | At least 1 IP or 2 OP claims | Any dx on claim |
| E10.3549 | Type 1 diabetes mellitus with proliferative diabetic retinopathy with combined traction retinal detachment and rhegmatogenous retinal detachment, unspecified eye | At least 1 IP or 2 OP claims | Any dx on claim |
| E10.3551 | Type 1 diabetes mellitus with stable proliferative diabetic retinopathy, right eye | At least 1 IP or 2 OP claims | Any dx on claim |
| E10.3552 | Type 1 diabetes mellitus with stable proliferative diabetic retinopathy, left eye | At least 1 IP or 2 OP claims | Any dx on claim |
| E10.3553 | Type 1 diabetes mellitus with stable proliferative diabetic retinopathy, bilateral | At least 1 IP or 2 OP claims | Any dx on claim |
| E10.3559 | Type 1 diabetes mellitus with stable proliferative diabetic retinopathy, unspecified eye | At least 1 IP or 2 OP claims | Any dx on claim |
| E10.359 | Type 1 diabetes mellitus with proliferative diabetic retinopathy without macular edema | At least 1 IP or 2 OP claims | Any dx on claim |
| E10.3591 | Type 1 diabetes mellitus with proliferative diabetic retinopathy without macular edema, right eye | At least 1 IP or 2 OP claims | Any dx on claim |
| E10.3592 | Type 1 diabetes mellitus with proliferative diabetic retinopathy without macular edema, left eye | At least 1 IP or 2 OP claims | Any dx on claim |
| E10.3593 | Type 1 diabetes mellitus with proliferative diabetic retinopathy without macular edema, bilateral | At least 1 IP or 2 OP claims | Any dx on claim |
| E10.3599 | Type 1 diabetes mellitus with proliferative diabetic retinopathy without macular edema, unspecified eye | At least 1 IP or 2 OP claims | Any dx on claim |
| E10.36 | Type 1 diabetes mellitus with diabetic cataract | At least 1 IP or 2 OP claims | Any dx on claim |
| E10.37X1 | Type 1 diabetes mellitus with diabetic macular edema, resolved following treatment, right eye | At least 1 IP or 2 OP claims | Any dx on claim |
| E10.37X2 | Type 1 diabetes mellitus with diabetic macular edema, resolved following treatment, left eye | At least 1 IP or 2 OP claims | Any dx on claim |
| E10.37X3 | Type 1 diabetes mellitus with diabetic macular edema, resolved following treatment, bilateral | At least 1 IP or 2 OP claims | Any dx on claim |
| E10.37X9 | Type 1 diabetes mellitus with diabetic macular edema, resolved following treatment, unspecified eye | At least 1 IP or 2 OP claims | Any dx on claim |
| E10.39 | Type 1 diabetes mellitus with other diabetic ophthalmic complication | At least 1 IP or 2 OP claims | Any dx on claim |
| E10.40 | Type 1 diabetes mellitus with diabetic neuropathy, unspecified | At least 1 IP or 2 OP claims | Any dx on claim |
| E10.41 | Type 1 diabetes mellitus with diabetic mononeuropathy | At least 1 IP or 2 OP claims | Any dx on claim |
| E10.42 | Type 1 diabetes mellitus with diabetic polyneuropathy | At least 1 IP or 2 OP claims | Any dx on claim |
| E10.43 | Type 1 diabetes mellitus with diabetic autonomic (poly)neuropathy | At least 1 IP or 2 OP claims | Any dx on claim |
| E10.44 | Type 1 diabetes mellitus with diabetic amyotrophy | At least 1 IP or 2 OP claims | Any dx on claim |
| E10.49 | Type 1 diabetes mellitus with other diabetic neurological complication | At least 1 IP or 2 OP claims | Any dx on claim |
| E10.51 | Type 1 diabetes mellitus with diabetic peripheral angiopathy without gangrene | At least 1 IP or 2 OP claims | Any dx on claim |
| E10.52 | Type 1 diabetes mellitus with diabetic peripheral angiopathy with gangrene | At least 1 IP or 2 OP claims | Any dx on claim |
| E10.59 | Type 1 diabetes mellitus with other circulatory complications | At least 1 IP or 2 OP claims | Any dx on claim |
| E10.610 | Type 1 diabetes mellitus with diabetic neuropathic arthropathy | At least 1 IP or 2 OP claims | Any dx on claim |
| E10.618 | Type 1 diabetes mellitus with other diabetic arthropathy | At least 1 IP or 2 OP claims | Any dx on claim |
| E10.620 | Type 1 diabetes mellitus with diabetic dermatitis | At least 1 IP or 2 OP claims | Any dx on claim |
| E10.621 | Type 1 diabetes mellitus with foot ulcer | At least 1 IP or 2 OP claims | Any dx on claim |
| E10.622 | Type 1 diabetes mellitus with other skin ulcer | At least 1 IP or 2 OP claims | Any dx on claim |
| E10.628 | Type 1 diabetes mellitus with other skin complications | At least 1 IP or 2 OP claims | Any dx on claim |
| E10.630 | Type 1 diabetes mellitus with periodontal disease | At least 1 IP or 2 OP claims | Any dx on claim |
| E10.638 | Type 1 diabetes mellitus with other oral complications | At least 1 IP or 2 OP claims | Any dx on claim |
| E10.641 | Type 1 diabetes mellitus with hypoglycemia with coma | At least 1 IP or 2 OP claims | Any dx on claim |
| E10.649 | Type 1 diabetes mellitus with hypoglycemia without coma | At least 1 IP or 2 OP claims | Any dx on claim |
| E10.65 | Type 1 diabetes mellitus with hyperglycemia | At least 1 IP or 2 OP claims | Any dx on claim |
| E10.69 | Type 1 diabetes mellitus with other specified complication | At least 1 IP or 2 OP claims | Any dx on claim |
| E10.8 | Type 1 diabetes mellitus with unspecified complications | At least 1 IP or 2 OP claims | Any dx on claim |
| E10.9 | Type 1 diabetes mellitus without complications | At least 1 IP or 2 OP claims | Any dx on claim |
| E11.00 | Type 2 diabetes mellitus with hyperosmolarity without nonketotic hyperglycemic-hyperosmolar coma (NKHHC) | At least 1 IP or 2 OP claims | Any dx on claim |
| E11.01 | Type 2 diabetes mellitus with hyperosmolarity with coma | At least 1 IP or 2 OP claims | Any dx on claim |
| E11.21 | Type 2 diabetes mellitus with diabetic nephropathy | At least 1 IP or 2 OP claims | Any dx on claim |
| E11.22 | Type 2 diabetes mellitus with diabetic chronic kidney disease | At least 1 IP or 2 OP claims | Any dx on claim |
| E11.29 | Type 2 diabetes mellitus with other diabetic kidney complication | At least 1 IP or 2 OP claims | Any dx on claim |
| E11.311 | Type 2 diabetes mellitus with unspecified diabetic retinopathy with macular edema | At least 1 IP or 2 OP claims | Any dx on claim |
| E11.319 | Type 2 diabetes mellitus with unspecified diabetic retinopathy without macular edema | At least 1 IP or 2 OP claims | Any dx on claim |
| E11.321 | Type 2 diabetes mellitus with mild nonproliferative diabetic retinopathy with macular edema | At least 1 IP or 2 OP claims | Any dx on claim |
| E11.3211 | Type 2 diabetes mellitus with mild nonproliferative diabetic retinopathy with macular edema, right eye | At least 1 IP or 2 OP claims | Any dx on claim |
| E11.3212 | Type 2 diabetes mellitus with mild nonproliferative diabetic retinopathy with macular edema, left eye | At least 1 IP or 2 OP claims | Any dx on claim |
| E11.3213 | Type 2 diabetes mellitus with mild nonproliferative diabetic retinopathy with macular edema, bilateral | At least 1 IP or 2 OP claims | Any dx on claim |
| E11.3219 | Type 2 diabetes mellitus with mild nonproliferative diabetic retinopathy with macular edema, unspecified eye | At least 1 IP or 2 OP claims | Any dx on claim |
| E11.329 | Type 2 diabetes mellitus with mild nonproliferative diabetic retinopathy without macular edema | At least 1 IP or 2 OP claims | Any dx on claim |
| E11.3291 | Type 2 diabetes mellitus with mild nonproliferative diabetic retinopathy without macular edema, right eye | At least 1 IP or 2 OP claims | Any dx on claim |
| E11.3292 | Type 2 diabetes mellitus with mild nonproliferative diabetic retinopathy without macular edema, left eye | At least 1 IP or 2 OP claims | Any dx on claim |
| E11.3293 | Type 2 diabetes mellitus with mild nonproliferative diabetic retinopathy without macular edema, bilateral | At least 1 IP or 2 OP claims | Any dx on claim |
| E11.3299 | Type 2 diabetes mellitus with mild nonproliferative diabetic retinopathy without macular edema, unspecified eye | At least 1 IP or 2 OP claims | Any dx on claim |
| E11.331 | Type 2 diabetes mellitus with moderate nonproliferative diabetic retinopathy with macular edema | At least 1 IP or 2 OP claims | Any dx on claim |
| E11.3311 | Type 2 diabetes mellitus with moderate nonproliferative diabetic retinopathy with macular edema, right eye | At least 1 IP or 2 OP claims | Any dx on claim |
| E11.3312 | Type 2 diabetes mellitus with moderate nonproliferative diabetic retinopathy with macular edema, left eye | At least 1 IP or 2 OP claims | Any dx on claim |
| E11.3313 | Type 2 diabetes mellitus with moderate nonproliferative diabetic retinopathy with macular edema, bilateral | At least 1 IP or 2 OP claims | Any dx on claim |
| E11.3319 | Type 2 diabetes mellitus with moderate nonproliferative diabetic retinopathy with macular edema, unspecified eye | At least 1 IP or 2 OP claims | Any dx on claim |
| E11.339 | Type 2 diabetes mellitus with moderate nonproliferative diabetic retinopathy without macular edema | At least 1 IP or 2 OP claims | Any dx on claim |
| E11.3391 | Type 2 diabetes mellitus with moderate nonproliferative diabetic retinopathy without macular edema, right eye | At least 1 IP or 2 OP claims | Any dx on claim |
| E11.3392 | Type 2 diabetes mellitus with moderate nonproliferative diabetic retinopathy without macular edema, left eye | At least 1 IP or 2 OP claims | Any dx on claim |
| E11.3393 | Type 2 diabetes mellitus with moderate nonproliferative diabetic retinopathy without macular edema, bilateral | At least 1 IP or 2 OP claims | Any dx on claim |
| E11.3399 | Type 2 diabetes mellitus with moderate nonproliferative diabetic retinopathy without macular edema, unspecified eye | At least 1 IP or 2 OP claims | Any dx on claim |
| E11.341 | Type 2 diabetes mellitus with severe nonproliferative diabetic retinopathy with macular edema | At least 1 IP or 2 OP claims | Any dx on claim |
| E11.3411 | Type 2 diabetes mellitus with severe nonproliferative diabetic retinopathy with macular edema, right eye | At least 1 IP or 2 OP claims | Any dx on claim |
| E11.3412 | Type 2 diabetes mellitus with severe nonproliferative diabetic retinopathy with macular edema, left eye | At least 1 IP or 2 OP claims | Any dx on claim |
| E11.3413 | Type 2 diabetes mellitus with severe nonproliferative diabetic retinopathy with macular edema, bilateral | At least 1 IP or 2 OP claims | Any dx on claim |
| E11.3419 | Type 2 diabetes mellitus with severe nonproliferative diabetic retinopathy with macular edema, unspecified eye | At least 1 IP or 2 OP claims | Any dx on claim |
| E11.349 | Type 2 diabetes mellitus with severe nonproliferative diabetic retinopathy without macular edema | At least 1 IP or 2 OP claims | Any dx on claim |
| E11.3491 | Type 2 diabetes mellitus with severe nonproliferative diabetic retinopathy without macular edema, right eye | At least 1 IP or 2 OP claims | Any dx on claim |
| E11.3492 | Type 2 diabetes mellitus with severe nonproliferative diabetic retinopathy without macular edema, left eye | At least 1 IP or 2 OP claims | Any dx on claim |
| E11.3493 | Type 2 diabetes mellitus with severe nonproliferative diabetic retinopathy without macular edema, bilateral | At least 1 IP or 2 OP claims | Any dx on claim |
| E11.3499 | Type 2 diabetes mellitus with severe nonproliferative diabetic retinopathy without macular edema, unspecified eye | At least 1 IP or 2 OP claims | Any dx on claim |
| E11.351 | Type 2 diabetes mellitus with proliferative diabetic retinopathy with macular edema | At least 1 IP or 2 OP claims | Any dx on claim |
| E11.3511 | Type 2 diabetes mellitus with proliferative diabetic retinopathy with macular edema, right eye | At least 1 IP or 2 OP claims | Any dx on claim |
| E11.3512 | Type 2 diabetes mellitus with proliferative diabetic retinopathy with macular edema, left eye | At least 1 IP or 2 OP claims | Any dx on claim |
| E11.3513 | Type 2 diabetes mellitus with proliferative diabetic retinopathy with macular edema, bilateral | At least 1 IP or 2 OP claims | Any dx on claim |
| E11.3519 | Type 2 diabetes mellitus with proliferative diabetic retinopathy with macular edema, unspecified eye | At least 1 IP or 2 OP claims | Any dx on claim |
| E11.3521 | Type 2 diabetes mellitus with proliferative diabetic retinopathy with traction retinal detachment involving the macula, right eye | At least 1 IP or 2 OP claims | Any dx on claim |
| E11.3522 | Type 2 diabetes mellitus with proliferative diabetic retinopathy with traction retinal detachment involving the macula, left eye | At least 1 IP or 2 OP claims | Any dx on claim |
| E11.3523 | Type 2 diabetes mellitus with proliferative diabetic retinopathy with traction retinal detachment involving the macula, bilateral | At least 1 IP or 2 OP claims | Any dx on claim |
| E11.3529 | Type 2 diabetes mellitus with proliferative diabetic retinopathy with traction retinal detachment involving the macula, unspecified eye | At least 1 IP or 2 OP claims | Any dx on claim |
| E11.3531 | Type 2 diabetes mellitus with proliferative diabetic retinopathy with traction retinal detachment not involving the macula, right eye | At least 1 IP or 2 OP claims | Any dx on claim |
| E11.3532 | Type 2 diabetes mellitus with proliferative diabetic retinopathy with traction retinal detachment not involving the macula, left eye | At least 1 IP or 2 OP claims | Any dx on claim |
| E11.3533 | Type 2 diabetes mellitus with proliferative diabetic retinopathy with traction retinal detachment not involving the macula, bilateral | At least 1 IP or 2 OP claims | Any dx on claim |
| E11.3539 | Type 2 diabetes mellitus with proliferative diabetic retinopathy with traction retinal detachment not involving the macula, unspecified eye | At least 1 IP or 2 OP claims | Any dx on claim |
| E11.3541 | Type 2 diabetes mellitus with proliferative diabetic retinopathy with combined traction retinal detachment and rhegmatogenous retinal detachment, right eye | At least 1 IP or 2 OP claims | Any dx on claim |
| E11.3542 | Type 2 diabetes mellitus with proliferative diabetic retinopathy with combined traction retinal detachment and rhegmatogenous retinal detachment, left eye | At least 1 IP or 2 OP claims | Any dx on claim |
| E11.3543 | Type 2 diabetes mellitus with proliferative diabetic retinopathy with combined traction retinal detachment and rhegmatogenous retinal detachment, bilateral | At least 1 IP or 2 OP claims | Any dx on claim |
| E11.3549 | Type 2 diabetes mellitus with proliferative diabetic retinopathy with combined traction retinal detachment and rhegmatogenous retinal detachment, unspecified eye | At least 1 IP or 2 OP claims | Any dx on claim |
| E11.3551 | Type 2 diabetes mellitus with stable proliferative diabetic retinopathy, right eye | At least 1 IP or 2 OP claims | Any dx on claim |
| E11.3552 | Type 2 diabetes mellitus with stable proliferative diabetic retinopathy, left eye | At least 1 IP or 2 OP claims | Any dx on claim |
| E11.3553 | Type 2 diabetes mellitus with stable proliferative diabetic retinopathy, bilateral | At least 1 IP or 2 OP claims | Any dx on claim |
| E11.3559 | Type 2 diabetes mellitus with stable proliferative diabetic retinopathy, unspecified eye | At least 1 IP or 2 OP claims | Any dx on claim |
| E11.359 | Type 2 diabetes mellitus with proliferative diabetic retinopathy without macular edema | At least 1 IP or 2 OP claims | Any dx on claim |
| E11.3591 | Type 2 diabetes mellitus with proliferative diabetic retinopathy without macular edema, right eye | At least 1 IP or 2 OP claims | Any dx on claim |
| E11.3592 | Type 2 diabetes mellitus with proliferative diabetic retinopathy without macular edema, left eye | At least 1 IP or 2 OP claims | Any dx on claim |
| E11.3593 | Type 2 diabetes mellitus with proliferative diabetic retinopathy without macular edema, bilateral | At least 1 IP or 2 OP claims | Any dx on claim |
| E11.3599 | Type 2 diabetes mellitus with proliferative diabetic retinopathy without macular edema, unspecified eye | At least 1 IP or 2 OP claims | Any dx on claim |
| E11.36 | Type 2 diabetes mellitus with diabetic cataract | At least 1 IP or 2 OP claims | Any dx on claim |
| E11.37X1 | Type 2 diabetes mellitus with diabetic macular edema, resolved following treatment, right eye | At least 1 IP or 2 OP claims | Any dx on claim |
| E11.37X2 | Type 2 diabetes mellitus with diabetic macular edema, resolved following treatment, left eye | At least 1 IP or 2 OP claims | Any dx on claim |
| E11.37X3 | Type 2 diabetes mellitus with diabetic macular edema, resolved following treatment, bilateral | At least 1 IP or 2 OP claims | Any dx on claim |
| E11.37X9 | Type 2 diabetes mellitus with diabetic macular edema, resolved following treatment, unspecified eye | At least 1 IP or 2 OP claims | Any dx on claim |
| E11.39 | Type 2 diabetes mellitus with other diabetic ophthalmic complication | At least 1 IP or 2 OP claims | Any dx on claim |
| E11.40 | Type 2 diabetes mellitus with diabetic neuropathy, unspecified | At least 1 IP or 2 OP claims | Any dx on claim |
| E11.41 | Type 2 diabetes mellitus with diabetic mononeuropathy | At least 1 IP or 2 OP claims | Any dx on claim |
| E11.42 | Type 2 diabetes mellitus with diabetic polyneuropathy | At least 1 IP or 2 OP claims | Any dx on claim |
| E11.43 | Type 2 diabetes mellitus with diabetic autonomic (poly)neuropathy | At least 1 IP or 2 OP claims | Any dx on claim |
| E11.44 | Type 2 diabetes mellitus with diabetic amyotrophy | At least 1 IP or 2 OP claims | Any dx on claim |
| E11.49 | Type 2 diabetes mellitus with other diabetic neurological complication | At least 1 IP or 2 OP claims | Any dx on claim |
| E11.51 | Type 2 diabetes mellitus with diabetic peripheral angiopathy without gangrene | At least 1 IP or 2 OP claims | Any dx on claim |
| E11.52 | Type 2 diabetes mellitus with diabetic peripheral angiopathy with gangrene | At least 1 IP or 2 OP claims | Any dx on claim |
| E11.59 | Type 2 diabetes mellitus with other circulatory complications | At least 1 IP or 2 OP claims | Any dx on claim |
| E11.610 | Type 2 diabetes mellitus with diabetic neuropathic arthropathy | At least 1 IP or 2 OP claims | Any dx on claim |
| E11.618 | Type 2 diabetes mellitus with other diabetic arthropathy | At least 1 IP or 2 OP claims | Any dx on claim |
| E11.620 | Type 2 diabetes mellitus with diabetic dermatitis | At least 1 IP or 2 OP claims | Any dx on claim |
| E11.621 | Type 2 diabetes mellitus with foot ulcer | At least 1 IP or 2 OP claims | Any dx on claim |
| E11.622 | Type 2 diabetes mellitus with other skin ulcer | At least 1 IP or 2 OP claims | Any dx on claim |
| E11.628 | Type 2 diabetes mellitus with other skin complications | At least 1 IP or 2 OP claims | Any dx on claim |
| E11.630 | Type 2 diabetes mellitus with periodontal disease | At least 1 IP or 2 OP claims | Any dx on claim |
| E11.638 | Type 2 diabetes mellitus with other oral complications | At least 1 IP or 2 OP claims | Any dx on claim |
| E11.641 | Type 2 diabetes mellitus with hypoglycemia with coma | At least 1 IP or 2 OP claims | Any dx on claim |
| E11.649 | Type 2 diabetes mellitus with hypoglycemia without coma | At least 1 IP or 2 OP claims | Any dx on claim |
| E11.65 | Type 2 diabetes mellitus with hyperglycemia | At least 1 IP or 2 OP claims | Any dx on claim |
| E11.69 | Type 2 diabetes mellitus with other specified complication | At least 1 IP or 2 OP claims | Any dx on claim |
| E11.8 | Type 2 diabetes mellitus with unspecified complications | At least 1 IP or 2 OP claims | Any dx on claim |
| E11.9 | Type 2 diabetes mellitus without complications | At least 1 IP or 2 OP claims | Any dx on claim |
| E13.00 | Other specified diabetes mellitus with hyperosmolarity without nonketotic hyperglycemic-hyperosmolar coma (NKHHC) | At least 1 IP or 2 OP claims | Any dx on claim |
| E13.01 | Other specified diabetes mellitus with hyperosmolarity with coma | At least 1 IP or 2 OP claims | Any dx on claim |
| E13.10 | Other specified diabetes mellitus with ketoacidosis without coma | At least 1 IP or 2 OP claims | Any dx on claim |
| E13.11 | Other specified diabetes mellitus with ketoacidosis with coma | At least 1 IP or 2 OP claims | Any dx on claim |
| E13.21 | Other specified diabetes mellitus with diabetic nephropathy | At least 1 IP or 2 OP claims | Any dx on claim |
| E13.22 | Other specified diabetes mellitus with diabetic chronic kidney disease | At least 1 IP or 2 OP claims | Any dx on claim |
| E13.29 | Other specified diabetes mellitus with other diabetic kidney complication | At least 1 IP or 2 OP claims | Any dx on claim |
| E13.311 | Other specified diabetes mellitus with unspecified diabetic retinopathy with macular edema | At least 1 IP or 2 OP claims | Any dx on claim |
| E13.319 | Other specified diabetes mellitus with unspecified diabetic retinopathy without macular edema | At least 1 IP or 2 OP claims | Any dx on claim |
| E13.321 | Other specified diabetes mellitus with mild nonproliferative diabetic retinopathy with macular edema | At least 1 IP or 2 OP claims | Any dx on claim |
| E13.3211 | Other specified diabetes mellitus with mild nonproliferative diabetic retinopathy with macular edema, right eye | At least 1 IP or 2 OP claims | Any dx on claim |
| E13.3212 | Other specified diabetes mellitus with mild nonproliferative diabetic retinopathy with macular edema, left eye | At least 1 IP or 2 OP claims | Any dx on claim |
| E13.3213 | Other specified diabetes mellitus with mild nonproliferative diabetic retinopathy with macular edema, bilateral | At least 1 IP or 2 OP claims | Any dx on claim |
| E13.3219 | Other specified diabetes mellitus with mild nonproliferative diabetic retinopathy with macular edema, unspecified eye | At least 1 IP or 2 OP claims | Any dx on claim |
| E13.329 | Other specified diabetes mellitus with mild nonproliferative diabetic retinopathy without macular edema | At least 1 IP or 2 OP claims | Any dx on claim |
| E13.3291 | Other specified diabetes mellitus with mild nonproliferative diabetic retinopathy without macular edema, right eye | At least 1 IP or 2 OP claims | Any dx on claim |
| E13.3292 | Other specified diabetes mellitus with mild nonproliferative diabetic retinopathy without macular edema, left eye | At least 1 IP or 2 OP claims | Any dx on claim |
| E13.3293 | Other specified diabetes mellitus with mild nonproliferative diabetic retinopathy without macular edema, bilateral | At least 1 IP or 2 OP claims | Any dx on claim |
| E13.3299 | Other specified diabetes mellitus with mild nonproliferative diabetic retinopathy without macular edema, unspecified eye | At least 1 IP or 2 OP claims | Any dx on claim |
| E13.331 | Other specified diabetes mellitus with moderate nonproliferative diabetic retinopathy with macular edema | At least 1 IP or 2 OP claims | Any dx on claim |
| E13.3311 | Other specified diabetes mellitus with moderate nonproliferative diabetic retinopathy with macular edema, right eye | At least 1 IP or 2 OP claims | Any dx on claim |
| E13.3312 | Other specified diabetes mellitus with moderate nonproliferative diabetic retinopathy with macular edema, left eye | At least 1 IP or 2 OP claims | Any dx on claim |
| E13.3313 | Other specified diabetes mellitus with moderate nonproliferative diabetic retinopathy with macular edema, bilateral | At least 1 IP or 2 OP claims | Any dx on claim |
| E13.3319 | Other specified diabetes mellitus with moderate nonproliferative diabetic retinopathy with macular edema, unspecified eye | At least 1 IP or 2 OP claims | Any dx on claim |
| E13.339 | Other specified diabetes mellitus with moderate nonproliferative diabetic retinopathy without macular edema | At least 1 IP or 2 OP claims | Any dx on claim |
| E13.3391 | Other specified diabetes mellitus with moderate nonproliferative diabetic retinopathy without macular edema, right eye | At least 1 IP or 2 OP claims | Any dx on claim |
| E13.3392 | Other specified diabetes mellitus with moderate nonproliferative diabetic retinopathy without macular edema, left eye | At least 1 IP or 2 OP claims | Any dx on claim |
| E13.3393 | Other specified diabetes mellitus with moderate nonproliferative diabetic retinopathy without macular edema, bilateral | At least 1 IP or 2 OP claims | Any dx on claim |
| E13.3399 | Other specified diabetes mellitus with moderate nonproliferative diabetic retinopathy without macular edema, unspecified eye | At least 1 IP or 2 OP claims | Any dx on claim |
| E13.341 | Other specified diabetes mellitus with severe nonproliferative diabetic retinopathy with macular edema | At least 1 IP or 2 OP claims | Any dx on claim |
| E13.3411 | Other specified diabetes mellitus with severe nonproliferative diabetic retinopathy with macular edema, right eye | At least 1 IP or 2 OP claims | Any dx on claim |
| E13.3412 | Other specified diabetes mellitus with severe nonproliferative diabetic retinopathy with macular edema, left eye | At least 1 IP or 2 OP claims | Any dx on claim |
| E13.3413 | Other specified diabetes mellitus with severe nonproliferative diabetic retinopathy with macular edema, bilateral | At least 1 IP or 2 OP claims | Any dx on claim |
| E13.3419 | Other specified diabetes mellitus with severe nonproliferative diabetic retinopathy with macular edema, unspecified eye | At least 1 IP or 2 OP claims | Any dx on claim |
| E13.349 | Other specified diabetes mellitus with severe nonproliferative diabetic retinopathy without macular edema | At least 1 IP or 2 OP claims | Any dx on claim |
| E13.3491 | Other specified diabetes mellitus with severe nonproliferative diabetic retinopathy without macular edema, right eye | At least 1 IP or 2 OP claims | Any dx on claim |
| E13.3492 | Other specified diabetes mellitus with severe nonproliferative diabetic retinopathy without macular edema, left eye | At least 1 IP or 2 OP claims | Any dx on claim |
| E13.3493 | Other specified diabetes mellitus with severe nonproliferative diabetic retinopathy without macular edema, bilateral | At least 1 IP or 2 OP claims | Any dx on claim |
| E13.3499 | Other specified diabetes mellitus with severe nonproliferative diabetic retinopathy without macular edema, unspecified eye | At least 1 IP or 2 OP claims | Any dx on claim |
| E13.351 | Other specified diabetes mellitus with proliferative diabetic retinopathy with macular edema | At least 1 IP or 2 OP claims | Any dx on claim |
| E13.3511 | Other specified diabetes mellitus with proliferative diabetic retinopathy with macular edema, right eye | At least 1 IP or 2 OP claims | Any dx on claim |
| E13.3512 | Other specified diabetes mellitus with proliferative diabetic retinopathy with macular edema, left eye | At least 1 IP or 2 OP claims | Any dx on claim |
| E13.3513 | Other specified diabetes mellitus with proliferative diabetic retinopathy with macular edema, bilateral | At least 1 IP or 2 OP claims | Any dx on claim |
| E13.3519 | Other specified diabetes mellitus with proliferative diabetic retinopathy with macular edema, unspecified eye | At least 1 IP or 2 OP claims | Any dx on claim |
| E13.3521 | Other specified diabetes mellitus with proliferative diabetic retinopathy with traction retinal detachment involving the macula, right eye | At least 1 IP or 2 OP claims | Any dx on claim |
| E13.3522 | Other specified diabetes mellitus with proliferative diabetic retinopathy with traction retinal detachment involving the macula, left eye | At least 1 IP or 2 OP claims | Any dx on claim |
| E13.3523 | Other specified diabetes mellitus with proliferative diabetic retinopathy with traction retinal detachment involving the macula, bilateral | At least 1 IP or 2 OP claims | Any dx on claim |
| E13.3529 | Other specified diabetes mellitus with proliferative diabetic retinopathy with traction retinal detachment involving the macula, unspecified eye | At least 1 IP or 2 OP claims | Any dx on claim |
| E13.3531 | Other specified diabetes mellitus with proliferative diabetic retinopathy with traction retinal detachment not involving the macula, right eye | At least 1 IP or 2 OP claims | Any dx on claim |
| E13.3532 | Other specified diabetes mellitus with proliferative diabetic retinopathy with traction retinal detachment not involving the macula, left eye | At least 1 IP or 2 OP claims | Any dx on claim |
| E13.3533 | Other specified diabetes mellitus with proliferative diabetic retinopathy with traction retinal detachment not involving the macula, bilateral | At least 1 IP or 2 OP claims | Any dx on claim |
| E13.3539 | Other specified diabetes mellitus with proliferative diabetic retinopathy with traction retinal detachment not involving the macula, unspecified eye | At least 1 IP or 2 OP claims | Any dx on claim |
| E13.3541 | Other specified diabetes mellitus with proliferative diabetic retinopathy with combined traction retinal detachment and rhegmatogenous retinal detachment, right eye | At least 1 IP or 2 OP claims | Any dx on claim |
| E13.3542 | Other specified diabetes mellitus with proliferative diabetic retinopathy with combined traction retinal detachment and rhegmatogenous retinal detachment, left eye | At least 1 IP or 2 OP claims | Any dx on claim |
| E13.3543 | Other specified diabetes mellitus with proliferative diabetic retinopathy with combined traction retinal detachment and rhegmatogenous retinal detachment, bilateral | At least 1 IP or 2 OP claims | Any dx on claim |
| E13.3549 | Other specified diabetes mellitus with proliferative diabetic retinopathy with combined traction retinal detachment and rhegmatogenous retinal detachment, unspecified eye | At least 1 IP or 2 OP claims | Any dx on claim |
| E13.3551 | Other specified diabetes mellitus with stable proliferative diabetic retinopathy, right eye | At least 1 IP or 2 OP claims | Any dx on claim |
| E13.3552 | Other specified diabetes mellitus with stable proliferative diabetic retinopathy, left eye | At least 1 IP or 2 OP claims | Any dx on claim |
| E13.3553 | Other specified diabetes mellitus with stable proliferative diabetic retinopathy, bilateral | At least 1 IP or 2 OP claims | Any dx on claim |
| E13.3559 | Other specified diabetes mellitus with stable proliferative diabetic retinopathy, unspecified eye | At least 1 IP or 2 OP claims | Any dx on claim |
| E13.359 | Other specified diabetes mellitus with proliferative diabetic retinopathy without macular edema | At least 1 IP or 2 OP claims | Any dx on claim |
| E13.3591 | Other specified diabetes mellitus with proliferative diabetic retinopathy without macular edema, right eye | At least 1 IP or 2 OP claims | Any dx on claim |
| E13.3592 | Other specified diabetes mellitus with proliferative diabetic retinopathy without macular edema, left eye | At least 1 IP or 2 OP claims | Any dx on claim |
| E13.3593 | Other specified diabetes mellitus with proliferative diabetic retinopathy without macular edema, bilateral | At least 1 IP or 2 OP claims | Any dx on claim |
| E13.3599 | Other specified diabetes mellitus with proliferative diabetic retinopathy without macular edema, unspecified eye | At least 1 IP or 2 OP claims | Any dx on claim |
| E13.36 | Other specified diabetes mellitus with diabetic cataract | At least 1 IP or 2 OP claims | Any dx on claim |
| E13.37X1 | Other specified diabetes mellitus with diabetic macular edema, resolved following treatment, right eye | At least 1 IP or 2 OP claims | Any dx on claim |
| E13.37X2 | Other specified diabetes mellitus with diabetic macular edema, resolved following treatment, left eye | At least 1 IP or 2 OP claims | Any dx on claim |
| E13.37X3 | Other specified diabetes mellitus with diabetic macular edema, resolved following treatment, bilateral | At least 1 IP or 2 OP claims | Any dx on claim |
| E13.37X9 | Other specified diabetes mellitus with diabetic macular edema, resolved following treatment, unspecified eye | At least 1 IP or 2 OP claims | Any dx on claim |
| E13.39 | Other specified diabetes mellitus with other diabetic ophthalmic complication | At least 1 IP or 2 OP claims | Any dx on claim |
| E13.40 | Other specified diabetes mellitus with diabetic neuropathy, unspecified | At least 1 IP or 2 OP claims | Any dx on claim |
| E13.41 | Other specified diabetes mellitus with diabetic mononeuropathy | At least 1 IP or 2 OP claims | Any dx on claim |
| E13.42 | Other specified diabetes mellitus with diabetic polyneuropathy | At least 1 IP or 2 OP claims | Any dx on claim |
| E13.43 | Other specified diabetes mellitus with diabetic autonomic (poly)neuropathy | At least 1 IP or 2 OP claims | Any dx on claim |
| E13.44 | Other specified diabetes mellitus with diabetic amyotrophy | At least 1 IP or 2 OP claims | Any dx on claim |
| E13.49 | Other specified diabetes mellitus with other diabetic neurological complication | At least 1 IP or 2 OP claims | Any dx on claim |
| E13.51 | Other specified diabetes mellitus with diabetic peripheral angiopathy without gangrene | At least 1 IP or 2 OP claims | Any dx on claim |
| E13.52 | Other specified diabetes mellitus with diabetic peripheral angiopathy with gangrene | At least 1 IP or 2 OP claims | Any dx on claim |
| E13.59 | Other specified diabetes mellitus with other circulatory complications | At least 1 IP or 2 OP claims | Any dx on claim |
| E13.610 | Other specified diabetes mellitus with diabetic neuropathic arthropathy | At least 1 IP or 2 OP claims | Any dx on claim |
| E13.618 | Other specified diabetes mellitus with other diabetic arthropathy | At least 1 IP or 2 OP claims | Any dx on claim |
| E13.620 | Other specified diabetes mellitus with diabetic dermatitis | At least 1 IP or 2 OP claims | Any dx on claim |
| E13.621 | Other specified diabetes mellitus with foot ulcer | At least 1 IP or 2 OP claims | Any dx on claim |
| E13.622 | Other specified diabetes mellitus with other skin ulcer | At least 1 IP or 2 OP claims | Any dx on claim |
| E13.628 | Other specified diabetes mellitus with other skin complications | At least 1 IP or 2 OP claims | Any dx on claim |
| E13.630 | Other specified diabetes mellitus with periodontal disease | At least 1 IP or 2 OP claims | Any dx on claim |
| E13.638 | Other specified diabetes mellitus with other oral complications | At least 1 IP or 2 OP claims | Any dx on claim |
| E13.641 | Other specified diabetes mellitus with hypoglycemia with coma | At least 1 IP or 2 OP claims | Any dx on claim |
| E13.649 | Other specified diabetes mellitus with hypoglycemia without coma | At least 1 IP or 2 OP claims | Any dx on claim |
| E13.65 | Other specified diabetes mellitus with hyperglycemia | At least 1 IP or 2 OP claims | Any dx on claim |
| E13.69 | Other specified diabetes mellitus with other specified complication | At least 1 IP or 2 OP claims | Any dx on claim |
| E13.8 | Other specified diabetes mellitus with unspecified complications | At least 1 IP or 2 OP claims | Any dx on claim |
| E13.9 | Other specified diabetes mellitus without complications | At least 1 IP or 2 OP claims | Any dx on claim |
| E13.37X1 | Other specified diabetes mellitus with diabetic macular edema, resolved following treatment, right eye | At least 1 IP or 2 OP claims | Any dx on claim |

#### Table A8. Heart Failure Cohort Criteria

| **ICD-10-CM Code** | **ICD-10-CM Code Description** | **Number/Type of Qualifying Claims** | **Diagnoses Used** |
| --- | --- | --- | --- |
| I09.81 | Rheumatic heart failure | At least 1 IP or 2 OP claims | 1^st^ or 2^nd^ dx on claim |
| I11.0 | Hypertensive heart disease with heart failure | At least 1 IP or 2 OP claims | 1^st^ or 2^nd^ dx on claim |
| I13.0 | Hypertensive heart and chronic kidney disease with heart failure and stage 1 through stage 4 chronic kidney disease, or unspecified chronic kidney disease | At least 1 IP or 2 OP claims | 1^st^ or 2^nd^ dx on claim |
| I13.2 | Hypertensive heart and chronic kidney disease with heart failure and with stage 5 chronic kidney disease, or end stage renal disease | At least 1 IP or 2 OP claims | 1^st^ or 2^nd^ dx on claim |
| I50.1 | Left ventricular failure | At least 1 IP or 2 OP claims | 1^st^ or 2^nd^ dx on claim |
| I50.20 | Unspecified systolic (congestive) heart failure | At least 1 IP or 2 OP claims | 1^st^ or 2^nd^ dx on claim |
| I50.21 | Acute systolic (congestive) heart failure | At least 1 IP or 2 OP claims | 1^st^ or 2^nd^ dx on claim |
| I50.22 | Chronic systolic (congestive) heart failure | At least 1 IP or 2 OP claims | 1^st^ or 2^nd^ dx on claim |
| I50.23 | Acute on chronic systolic (congestive) heart failure | At least 1 IP or 2 OP claims | 1^st^ or 2^nd^ dx on claim |
| I50.30 | Unspecified diastolic (congestive) heart failure | At least 1 IP or 2 OP claims | 1^st^ or 2^nd^ dx on claim |
| I50.31 | Acute diastolic (congestive) heart failure | At least 1 IP or 2 OP claims | 1^st^ or 2^nd^ dx on claim |
| I50.32 | Chronic diastolic (congestive) heart failure | At least 1 IP or 2 OP claims | 1^st^ or 2^nd^ dx on claim |
| I50.33 | Acute on chronic diastolic (congestive) heart failure | At least 1 IP or 2 OP claims | 1^st^ or 2^nd^ dx on claim |
| I50.40 | Unspecified combined systolic (congestive) and diastolic(congestive) heart failure | At least 1 IP or 2 OP claims | 1^st^ or 2^nd^ dx on claim |
| I50.41 | Acute combined systolic and diastolic heart failure | At least 1 IP or 2 OP claims | 1^st^ or 2^nd^ dx on claim |
| I50.42 | Chronic combined systolic (congestive) and diastolic | At least 1 IP or 2 OP claims | 1^st^ or 2^nd^ dx on claim |
| I50.43 | Acute on chronic combined systolic (congestive) and diastolic heart failure | At least 1 IP or 2 OP claims | 1^st^ or 2^nd^ dx on claim |
| I50.810 | Right heart failure, unspecified | At least 1 IP or 2 OP claims | 1^st^ or 2^nd^ dx on claim |
| I50.811 | Acute right heart failure | At least 1 IP or 2 OP claims | 1^st^ or 2^nd^ dx on claim |
| I50.812 | Chronic right heart failure | At least 1 IP or 2 OP claims | 1^st^ or 2^nd^ dx on claim |
| I50.813 | Acute on chronic right heart failure | At least 1 IP or 2 OP claims | 1^st^ or 2^nd^ dx on claim |
| I50.814 | Right heart failure due to left heart failure | At least 1 IP or 2 OP claims | 1^st^ or 2^nd^ dx on claim |
| I50.82 | Biventricular heart failure | At least 1 IP or 2 OP claims | 1^st^ or 2^nd^ dx on claim |
| I50.83 | High output heart failure | At least 1 IP or 2 OP claims | 1^st^ or 2^nd^ dx on claim |
| I50.84 | End stage heart failure | At least 1 IP or 2 OP claims | 1^st^ or 2^nd^ dx on claim |
| I50.89 | Other heart failure | At least 1 IP or 2 OP claims | 1^st^ or 2^nd^ dx on claim |
| I50.9 | Heart failure, unspecified | At least 1 IP or 2 OP claims | 1^st^ or 2^nd^ dx on claim |

#### Table A9. Stroke and Transient Ischemic Attack Cohort Criteria

| **ICD-10-CM Code** | **ICD-10-CM Code Description** | **Number/Type of Claims to Qualify** | **Diagnoses Used** |
| --- | --- | --- | --- |
| G45.0 | Vertebro-basilar artery syndrome | At least 1 IP or 2 OP claims | Any dx on claim |
| G45.1 | Carotid artery syndrome (hemispheric) | At least 1 IP or 2 OP claims | Any dx on claim |
| G45.2 | Multiple and bilateral precerebral artery syndromes | At least 1 IP or 2 OP claims | Any dx on claim |
| G45.8 | Other transient cerebral ischemic attacks and related syndromes | At least 1 IP or 2 OP claims | Any dx on claim |
| G45.9 | Transient cerebral ischemic attack, unspecified | At least 1 IP or 2 OP claims | Any dx on claim |
| G46.0 | Middle cerebral artery syndrome | At least 1 IP or 2 OP claims | Any dx on claim |
| G46.1 | Anterior cerebral artery syndrome | At least 1 IP or 2 OP claims | Any dx on claim |
| G46.2 | Posterior cerebral artery syndrome | At least 1 IP or 2 OP claims | Any dx on claim |
| G46.3 | Brain stem stroke syndrome | At least 1 IP or 2 OP claims | Any dx on claim |
| G46.4 | Cerebellar stroke syndrome | At least 1 IP or 2 OP claims | Any dx on claim |
| G46.5 | Pure motor lacunar syndrome | At least 1 IP or 2 OP claims | Any dx on claim |
| G46.6 | Pure sensory lacunar syndrome | At least 1 IP or 2 OP claims | Any dx on claim |
| G46.7 | Other lacunar syndromes | At least 1 IP or 2 OP claims | Any dx on claim |
| G46.8 | Other vascular syndromes of brain in cerebrovascular diseases | At least 1 IP or 2 OP claims | Any dx on claim |
| G97.31 | Intraoperative hemorrhage and hematoma of a nervous system organ or structure complicating a nervous system procedure | At least 1 IP or 2 OP claims | Any dx on claim |
| G97.32 | Intraoperative hemorrhage and hematoma of a nervous system organ or structure complicating other procedure | At least 1 IP or 2 OP claims | Any dx on claim |
| I60.00 | Nontraumatic subarachnoid hemorrhage from unspecified carotid siphon and bifurcation | At least 1 IP or 2 OP claims | Any dx on claim |
| I60.01 | Nontraumatic subarachnoid hemorrhage from right carotid siphon and bifurcation | At least 1 IP or 2 OP claims | Any dx on claim |
| I60.02 | Nontraumatic subarachnoid hemorrhage from left carotid siphon and bifurcation | At least 1 IP or 2 OP claims | Any dx on claim |
| I60.10 | Nontraumatic subarachnoid hemorrhage from unspecified middle cerebral artery | At least 1 IP or 2 OP claims | Any dx on claim |
| I60.11 | Nontraumatic subarachnoid hemorrhage from right middle cerebral artery | At least 1 IP or 2 OP claims | Any dx on claim |
| I60.12 | Nontraumatic subarachnoid hemorrhage from left middle cerebral artery | At least 1 IP or 2 OP claims | Any dx on claim |
| I60.20 | Nontraumatic subarachnoid hemorrhage from anterior communicating artery | At least 1 IP or 2 OP claims | Any dx on claim |
| I60.21 | Nontraumatic subarachnoid hemorrhage from right anterior communicating artery | At least 1 IP or 2 OP claims | Any dx on claim |
| I60.22 | Nontraumatic subarachnoid hemorrhage from left anterior communicating artery | At least 1 IP or 2 OP claims | Any dx on claim |
| I60.30 | Nontraumatic subarachnoid hemorrhage from unspecified posterior communicating artery | At least 1 IP or 2 OP claims | Any dx on claim |
| I60.31 | Nontraumatic subarachnoid hemorrhage from right posterior communicating artery | At least 1 IP or 2 OP claims | Any dx on claim |
| I60.32 | Nontraumatic subarachnoid hemorrhage from left posterior communicating artery | At least 1 IP or 2 OP claims | Any dx on claim |
| I60.4 | Nontraumatic subarachnoid hemorrhage from basilar artery | At least 1 IP or 2 OP claims | Any dx on claim |
| I60.50 | Nontraumatic subarachnoid hemorrhage from unspecified vertebral artery | At least 1 IP or 2 OP claims | Any dx on claim |
| I60.51 | Nontraumatic subarachnoid hemorrhage from right vertebral artery | At least 1 IP or 2 OP claims | Any dx on claim |
| I60.52 | Nontraumatic subarachnoid hemorrhage from left vertebral artery | At least 1 IP or 2 OP claims | Any dx on claim |
| I60.6 | Nontraumatic subarachnoid hemorrhage from other intracranial arteries | At least 1 IP or 2 OP claims | Any dx on claim |
| I60.7 | Nontraumatic subarachnoid hemorrhage from unspecified intracranial artery | At least 1 IP or 2 OP claims | Any dx on claim |
| I60.8 | Other nontraumatic subarachnoid hemorrhage | At least 1 IP or 2 OP claims | Any dx on claim |
| I60.9 | Nontraumatic subarachnoid hemorrhage, unspecified | At least 1 IP or 2 OP claims | Any dx on claim |
| I61.0 | Nontraumatic intracerebral hemorrhage in hemisphere, subcortical | At least 1 IP or 2 OP claims | Any dx on claim |
| I61.1 | Nontraumatic intracerebral hemorrhage in hemisphere, cortical | At least 1 IP or 2 OP claims | Any dx on claim |
| I61.2 | Nontraumatic intracerebral hemorrhage in hemisphere, unspecified | At least 1 IP or 2 OP claims | Any dx on claim |
| I61.3 | Nontraumatic intracerebral hemorrhage in brain stem | At least 1 IP or 2 OP claims | Any dx on claim |
| I61.4 | Nontraumatic intracerebral hemorrhage in cerebellum | At least 1 IP or 2 OP claims | Any dx on claim |
| I61.5 | Nontraumatic intracerebral hemorrhage, intraventricular | At least 1 IP or 2 OP claims | Any dx on claim |
| I61.6 | Nontraumatic intracerebral hemorrhage, multiple localized | At least 1 IP or 2 OP claims | Any dx on claim |
| I61.8 | Other nontraumatic intracerebral hemorrhage | At least 1 IP or 2 OP claims | Any dx on claim |
| I61.9 | Nontraumatic intracerebral hemorrhage, unspecified | At least 1 IP or 2 OP claims | Any dx on claim |
| I63.00 | Cerebral infarction due to thrombosis of unspecified precerebral artery | At least 1 IP or 2 OP claims | Any dx on claim |
| I63.02 | Cerebral infarction due to thrombosis of basilar artery | At least 1 IP or 2 OP claims | Any dx on claim |
| I63.011 | Cerebral infarction due to thrombosis of right vertebral artery | At least 1 IP or 2 OP claims | Any dx on claim |
| I63.012 | Cerebral infarction due to thrombosis of left vertebral artery | At least 1 IP or 2 OP claims | Any dx on claim |
| I63.013 | Cerebral infarction due to thrombosis of bilateral vertebral arteries | At least 1 IP or 2 OP claims | Any dx on claim |
| I63.019 | Cerebral infarction due to thrombosis of unspecified vertebral artery | At least 1 IP or 2 OP claims | Any dx on claim |
| I63.031 | Cerebral infarction due to thrombosis of right carotid artery | At least 1 IP or 2 OP claims | Any dx on claim |
| I63.032 | Cerebral infarction due to thrombosis of left carotid artery | At least 1 IP or 2 OP claims | Any dx on claim |
| I63.033 | Cerebral infarction due to thrombosis of bilateral carotid arteries | At least 1 IP or 2 OP claims | Any dx on claim |
| I63.039 | Cerebral infarction due to thrombosis of unspecified carotid artery | At least 1 IP or 2 OP claims | Any dx on claim |
| I63.09 | Cerebral infarction due to thrombosis of other precerebral artery | At least 1 IP or 2 OP claims | Any dx on claim |
| I63.10 | Cerebral infarction due to embolism of unspecified precerebral artery | At least 1 IP or 2 OP claims | Any dx on claim |
| I63.111 | Cerebral infarction due to embolism of right vertebral artery | At least 1 IP or 2 OP claims | Any dx on claim |
| I63.112 | Cerebral infarction due to embolism of left vertebral artery | At least 1 IP or 2 OP claims | Any dx on claim |
| I63.113 | Cerebral infarction due to embolism of bilateral vertebral arteries | At least 1 IP or 2 OP claims | Any dx on claim |
| I63.119 | Cerebral infarction due to embolism of unspecified vertebral artery | At least 1 IP or 2 OP claims | Any dx on claim |
| I63.12 | Cerebral infarction due to embolism of basilar artery | At least 1 IP or 2 OP claims | Any dx on claim |
| I63.131 | Cerebral infarction due to embolism of right carotid artery | At least 1 IP or 2 OP claims | Any dx on claim |
| I63.132 | Cerebral infarction due to embolism of left carotid artery | At least 1 IP or 2 OP claims | Any dx on claim |
| I63.133 | Cerebral infarction due to embolism of bilateral carotid arteries | At least 1 IP or 2 OP claims | Any dx on claim |
| I63.139 | Cerebral infarction due to embolism of unspecified carotid artery | At least 1 IP or 2 OP claims | Any dx on claim |
| I63.19 | Cerebral infarction due to embolism of other precerebral artery | At least 1 IP or 2 OP claims | Any dx on claim |
| I63.20 | Cerebral infarction due to unspecified occlusion or stenosis of unspecified | At least 1 IP or 2 OP claims | Any dx on claim |
| I63.211 | Cerebral infarction due to unspecified occlusion or stenosis of right vertebral arteries | At least 1 IP or 2 OP claims | Any dx on claim |
| I63.212 | Cerebral infarction due to unspecified occlusion or stenosis of left vertebral arteries | At least 1 IP or 2 OP claims | Any dx on claim |
| I63.213 | Cerebral infarction due to unspecified occlusion or stenosis of bilateral vertebral arteries | At least 1 IP or 2 OP claims | Any dx on claim |
| I63.219 | Cerebral infarction due to unspecified occlusion or stenosis of unspecified vertebral arteries | At least 1 IP or 2 OP claims | Any dx on claim |
| I63.22 | Cerebral infarction due to unspecified occlusion or stenosis of basilar arteries | At least 1 IP or 2 OP claims | Any dx on claim |
| I63.231 | Cerebral infarction due to unspecified occlusion or stenosis of right carotid arteries | At least 1 IP or 2 OP claims | Any dx on claim |
| I63.232 | Cerebral infarction due to unspecified occlusion or stenosis of left carotid arteries | At least 1 IP or 2 OP claims | Any dx on claim |
| I63.233 | Cerebral infarction due to unspecified occlusion or stenosis of bilateral carotid arteries | At least 1 IP or 2 OP claims | Any dx on claim |
| I63.239 | Cerebral infarction due to unspecified occlusion or stenosis of unspecified carotid arteries | At least 1 IP or 2 OP claims | Any dx on claim |
| I63.29 | Cerebral infarction due to unspecified occlusion or stenosis of other precerebral arteries | At least 1 IP or 2 OP claims | Any dx on claim |
| I63.30 | Cerebral infarction due to thrombosis of unspecified cerebral artery | At least 1 IP or 2 OP claims | Any dx on claim |
| I63.311 | Cerebral infarction due to thrombosis of right middle cerebral artery | At least 1 IP or 2 OP claims | Any dx on claim |
| I63.312 | Cerebral infarction due to thrombosis of left middle cerebral artery | At least 1 IP or 2 OP claims | Any dx on claim |
| I63.313 | Cerebral infarction due to thrombosis of bilateral middle cerebral arteries | At least 1 IP or 2 OP claims | Any dx on claim |
| I63.319 | Cerebral infarction due to thrombosis of unspecified middle cerebral artery | At least 1 IP or 2 OP claims | Any dx on claim |
| I63.321 | Cerebral infarction due to thrombosis of right anterior cerebral artery | At least 1 IP or 2 OP claims | Any dx on claim |
| I63.322 | Cerebral infarction due to thrombosis of left anterior cerebral artery | At least 1 IP or 2 OP claims | Any dx on claim |
| I63.323 | Cerebral infarction due to thrombosis of bilateral anterior arteries | At least 1 IP or 2 OP claims | Any dx on claim |
| I63.329 | Cerebral infarction due to thrombosis of unspecified anterior cerebral artery | At least 1 IP or 2 OP claims | Any dx on claim |
| I63.331 | Cerebral infarction due to thrombosis of right posterior cerebral artery | At least 1 IP or 2 OP claims | Any dx on claim |
| I63.332 | Cerebral infarction due to thrombosis of left posterior cerebral artery | At least 1 IP or 2 OP claims | Any dx on claim |
| I63.333 | Cerebral infarction to thrombosis of bilateral posterior arteries | At least 1 IP or 2 OP claims | Any dx on claim |
| I63.339 | Cerebral infarction due to thrombosis of unspecified posterior cerebral artery | At least 1 IP or 2 OP claims | Any dx on claim |
| I63.341 | Cerebral infarction due to thrombosis of right cerebellar artery | At least 1 IP or 2 OP claims | Any dx on claim |
| I63.342 | Cerebral infarction due to thrombosis of left cerebellar artery | At least 1 IP or 2 OP claims | Any dx on claim |
| I63.343 | Cerebral infarction to thrombosis of bilateral cerebellar arteries | At least 1 IP or 2 OP claims | Any dx on claim |
| I63.349 | Cerebral infarction due to thrombosis of unspecified cerebellar artery | At least 1 IP or 2 OP claims | Any dx on claim |
| I63.39 | Cerebral infarction due to thrombosis of other cerebral artery | At least 1 IP or 2 OP claims | Any dx on claim |
| I63.40 | Cerebral infarction due to embolism of unspecified cerebral artery | At least 1 IP or 2 OP claims | Any dx on claim |
| I63.411 | Cerebral infarction due to embolism of right middle cerebral artery | At least 1 IP or 2 OP claims | Any dx on claim |
| I63.412 | Cerebral infarction due to embolism of left middle cerebral artery | At least 1 IP or 2 OP claims | Any dx on claim |
| I63.413 | Cerebral infarction due to embolism of bilateral middle cerebral arteries | At least 1 IP or 2 OP claims | Any dx on claim |
| I63.419 | Cerebral infarction due to embolism of unspecified middle cerebral artery | At least 1 IP or 2 OP claims | Any dx on claim |
| I63.421 | Cerebral infarction due to embolism of right anterior cerebral artery | At least 1 IP or 2 OP claims | Any dx on claim |
| I63.422 | Cerebral infarction due to embolism of left anterior cerebral artery | At least 1 IP or 2 OP claims | Any dx on claim |
| I63.423 | Cerebral infarction due to embolism of bilateral anterior cerebral arteries | At least 1 IP or 2 OP claims | Any dx on claim |
| I63.429 | Cerebral infarction due to embolism of unspecified anterior cerebral artery | At least 1 IP or 2 OP claims | Any dx on claim |
| I63.431 | Cerebral infarction due to embolism of right posterior cerebral artery | At least 1 IP or 2 OP claims | Any dx on claim |
| I63.432 | Cerebral infarction due to embolism of left posterior cerebral artery | At least 1 IP or 2 OP claims | Any dx on claim |
| I63.433 | Cerebral infarction due to embolism of bilateral posterior cerebral arteries | At least 1 IP or 2 OP claims | Any dx on claim |
| I63.439 | Cerebral infarction due to embolism of unspecified posterior cerebral artery | At least 1 IP or 2 OP claims | Any dx on claim |
| I63.441 | Cerebral infarction due to embolism of right cerebellar artery | At least 1 IP or 2 OP claims | Any dx on claim |
| I63.442 | Cerebral infarction due to embolism of left cerebellar artery | At least 1 IP or 2 OP claims | Any dx on claim |
| I63.443 | Cerebral infarction due to embolism of bilateral cerebellar arteries | At least 1 IP or 2 OP claims | Any dx on claim |
| I63.449 | Cerebral infarction due to embolism of unspecified cerebellar artery | At least 1 IP or 2 OP claims | Any dx on claim |
| I63.49 | Cerebral infarction due to embolism of other cerebral artery | At least 1 IP or 2 OP claims | Any dx on claim |
| I63.50 | Cerebral infarction due to unspecified occlusion or stenosis of unspecified cerebral artery | At least 1 IP or 2 OP claims | Any dx on claim |
| I63.511 | Cerebral infarction due to unspecified occlusion or stenosis of right middle cerebral artery | At least 1 IP or 2 OP claims | Any dx on claim |
| I63.512 | Cerebral infarction due to unspecified occlusion or stenosis of left middle cerebral artery | At least 1 IP or 2 OP claims | Any dx on claim |
| I63.513 | Cerebral infarction due to unspecified occlusion or stenosis of bilateral middle arteries | At least 1 IP or 2 OP claims | Any dx on claim |
| I63.519 | Cerebral infarction due to unspecified occlusion or stenosis of unspecified middle cerebral artery | At least 1 IP or 2 OP claims | Any dx on claim |
| I63.521 | Cerebral infarction due to unspecified occlusion or stenosis of right anterior cerebral artery | At least 1 IP or 2 OP claims | Any dx on claim |
| I63.522 | Cerebral infarction due to unspecified occlusion or stenosis of left anterior cerebral artery | At least 1 IP or 2 OP claims | Any dx on claim |
| I63.523 | Cerebral infarction due to unspecified occlusion or stenosis of bilateral anterior arteries | At least 1 IP or 2 OP claims | Any dx on claim |
| I63.529 | Cerebral infarction due to unspecified occlusion or stenosis of unspecified anterior cerebral artery | At least 1 IP or 2 OP claims | Any dx on claim |
| I63.531 | Cerebral infarction due to unspecified occlusion or stenosis of right posterior cerebral artery | At least 1 IP or 2 OP claims | Any dx on claim |
| I63.532 | Cerebral infarction due to unspecified occlusion or stenosis of left posterior cerebral artery | At least 1 IP or 2 OP claims | Any dx on claim |
| I63.533 | Cerebral infarction due to unspecified occlusion or stenosis of bilateral posterior arteries | At least 1 IP or 2 OP claims | Any dx on claim |
| I63.539 | Cerebral infarction due to unspecified occlusion or stenosis of unspecified posterior cerebral artery | At least 1 IP or 2 OP claims | Any dx on claim |
| I63.541 | Cerebral infarction due to unspecified occlusion or stenosis of right cerebellar artery | At least 1 IP or 2 OP claims | Any dx on claim |
| I63.542 | Cerebral infarction due to unspecified occlusion or stenosis of left cerebellar artery | At least 1 IP or 2 OP claims | Any dx on claim |
| I63.543 | Cerebral infarction due to unspecified occlusion or stenosis of bilateral cerebellar arteries | At least 1 IP or 2 OP claims | Any dx on claim |
| I63.549 | Cerebral infarction due to unspecified occlusion or stenosis of unspecified cerebellar artery | At least 1 IP or 2 OP claims | Any dx on claim |
| I63.59 | Cerebral infarction due to unspecified occlusion or stenosis of other cerebral artery | At least 1 IP or 2 OP claims | Any dx on claim |
| I63.6 | Cerebral infarction due to cerebral venous thrombosis, nonpyogenic | At least 1 IP or 2 OP claims | Any dx on claim |
| I63.8 | Other cerebral infarction | At least 1 IP or 2 OP claims | Any dx on claim |
| I63.81 | Other cerebral infarction due to occlusion or stenosis of small artery | At least 1 IP or 2 OP claims | Any dx on claim |
| I63.89 | Other cerebral infarction | At least 1 IP or 2 OP claims | Any dx on claim |
| I63.9 | Cerebral infarction, unspecified | At least 1 IP or 2 OP claims | Any dx on claim |
| I66.01 | Occlusion and stenosis of right middle cerebral artery | At least 1 IP or 2 OP claims | Any dx on claim |
| I66.02 | Occlusion and stenosis of left middle cerebral artery | At least 1 IP or 2 OP claims | Any dx on claim |
| I66.03 | Occlusion and stenosis of bilateral middle cerebral arteries | At least 1 IP or 2 OP claims | Any dx on claim |
| I66.09 | Occlusion and stenosis of unspecified middle cerebral artery | At least 1 IP or 2 OP claims | Any dx on claim |
| I66.11 | Occlusion and stenosis of right anterior cerebral artery | At least 1 IP or 2 OP claims | Any dx on claim |
| I66.12 | Occlusion and stenosis of left anterior cerebral artery | At least 1 IP or 2 OP claims | Any dx on claim |
| I66.13 | Occlusion and stenosis of bilateral anterior cerebral arteries | At least 1 IP or 2 OP claims | Any dx on claim |
| I66.19 | Occlusion and stenosis of unspecified anterior cerebral artery | At least 1 IP or 2 OP claims | Any dx on claim |
| I66.21 | Occlusion and stenosis of right posterior cerebral artery | At least 1 IP or 2 OP claims | Any dx on claim |
| I66.22 | Occlusion and stenosis of left posterior cerebral artery | At least 1 IP or 2 OP claims | Any dx on claim |
| I66.23 | Occlusion and stenosis of bilateral posterior cerebral arteries | At least 1 IP or 2 OP claims | Any dx on claim |
| I66.29 | Occlusion and stenosis of unspecified posterior cerebral artery | At least 1 IP or 2 OP claims | Any dx on claim |
| I66.3 | Occlusion and stenosis of cerebellar arteries | At least 1 IP or 2 OP claims | Any dx on claim |
| I66.8 | Occlusion and stenosis of other cerebral arteries | At least 1 IP or 2 OP claims | Any dx on claim |
| I66.9 | Occlusion and stenosis of unspecified cerebral artery | At least 1 IP or 2 OP claims | Any dx on claim |
| I67.841 | Reversible cerebrovascular vasoconstriction syndrome | At least 1 IP or 2 OP claims | Any dx on claim |
| I67.848 | Other cerebrovascular vasospasm and vasoconstriction | At least 1 IP or 2 OP claims | Any dx on claim |
| I67.85 | Hereditary cerebrovascular diseases | At least 1 IP or 2 OP claims | Any dx on claim |
| I67.850 | Cerebral autosomal dominant arteriopathy with subcortical infarcts and leukoencephalopathy | At least 1 IP or 2 OP claims | Any dx on claim |
| I67.858 | Other hereditary cerebrovascular disease | At least 1 IP or 2 OP claims | Any dx on claim |
| I67.89 | Other cerebrovascular disease | At least 1 IP or 2 OP claims | Any dx on claim |
| I69.00 | Unspecified sequelae of nontraumatic subarachnoid hemorrhage | At least 1 IP or 2 OP claims | Any dx on claim |
| I69.01 | Cognitive deficits following nontraumatic subarachnoid hemorrhage | At least 1 IP or 2 OP claims | Any dx on claim |
| I69.010 | Attention and concentration deficit following nontraumatic subarachnoid hemorrhage | At least 1 IP or 2 OP claims | Any dx on claim |
| I69.011 | Memory deficit following nontraumatic subarachnoid hemorrhage | At least 1 IP or 2 OP claims | Any dx on claim |
| I69.012 | Visuospatial deficit and spatial neglect following nontraumatic subarachnoid hemorrhage | At least 1 IP or 2 OP claims | Any dx on claim |
| I69.013 | Psychomotor deficit following nontraumatic subarachnoid hemorrhage | At least 1 IP or 2 OP claims | Any dx on claim |
| I69.014 | Frontal lobe and executive function deficit following nontraumatic subarachnoid hemorrhage | At least 1 IP or 2 OP claims | Any dx on claim |
| I69.015 | Cognitive social or emotional deficit following nontraumatic subarachnoid hemorrhage | At least 1 IP or 2 OP claims | Any dx on claim |
| I69.018 | Other symptoms and signs involving cognitive functions following nontraumatic subarachnoid hemorrhage | At least 1 IP or 2 OP claims | Any dx on claim |
| I69.019 | Unspecified symptoms and signs involving cognitive functions following nontraumatic subarachnoid hemorrhage | At least 1 IP or 2 OP claims | Any dx on claim |
| I69.020 | Aphasia following nontraumatic subarachnoid hemorrhage | At least 1 IP or 2 OP claims | Any dx on claim |
| I69.021 | Dysphasia following nontraumatic subarachnoid hemorrhage | At least 1 IP or 2 OP claims | Any dx on claim |
| I69.022 | Dysarthria following nontraumatic subarachnoid hemorrhage | At least 1 IP or 2 OP claims | Any dx on claim |
| I69.023 | Fluency disorder following nontraumatic subarachnoid hemorrhage | At least 1 IP or 2 OP claims | Any dx on claim |
| I69.028 | Other speech and language deficits following nontraumatic subarachnoid hemorrhage | At least 1 IP or 2 OP claims | Any dx on claim |
| I69.031 | Monoplegia of upper limb following nontraumatic subarachnoid hemorrhage affecting right dominant side | At least 1 IP or 2 OP claims | Any dx on claim |
| I69.032 | Monoplegia of upper limb following nontraumatic subarachnoid hemorrhage affecting left dominant side | At least 1 IP or 2 OP claims | Any dx on claim |
| I69.033 | Monoplegia of upper limb following nontraumatic subarachnoid hemorrhage affecting right non-dominant side | At least 1 IP or 2 OP claims | Any dx on claim |
| I69.034 | Monoplegia of upper limb following nontraumatic subarachnoid hemorrhage affecting left non-dominant side | At least 1 IP or 2 OP claims | Any dx on claim |
| I69.039 | Monoplegia of upper limb following nontraumatic subarachnoid hemorrhage affecting unspecified side | At least 1 IP or 2 OP claims | Any dx on claim |
| I69.041 | Monoplegia of lower limb following nontraumatic subarachnoid hemorrhage affecting right dominant side | At least 1 IP or 2 OP claims | Any dx on claim |
| I69.042 | Monoplegia of lower limb following nontraumatic subarachnoid hemorrhage affecting left dominant side | At least 1 IP or 2 OP claims | Any dx on claim |
| I69.043 | Monoplegia of lower limb following nontraumatic subarachnoid hemorrhage affecting right non-dominant side | At least 1 IP or 2 OP claims | Any dx on claim |
| I69.044 | Monoplegia of lower limb following nontraumatic subarachnoid hemorrhage affecting left non-dominant side | At least 1 IP or 2 OP claims | Any dx on claim |
| I69.049 | Monoplegia of lower limb following nontraumatic subarachnoid hemorrhage affecting unspecified side | At least 1 IP or 2 OP claims | Any dx on claim |
| I69.051 | Hemiplegia and hemiparesis following nontraumatic subarachnoid hemorrhage affecting right dominant side | At least 1 IP or 2 OP claims | Any dx on claim |
| I69.052 | Hemiplegia and hemiparesis following nontraumatic subarachnoid hemorrhage affecting left dominant side | At least 1 IP or 2 OP claims | Any dx on claim |
| I69.053 | Hemiplegia and hemiparesis following nontraumatic subarachnoid hemorrhage affecting right non-dominant side | At least 1 IP or 2 OP claims | Any dx on claim |
| I69.054 | Hemiplegia and hemiparesis following nontraumatic subarachnoid hemorrhage affecting left non-dominant side | At least 1 IP or 2 OP claims | Any dx on claim |
| I69.059 | Hemiplegia and hemiparesis following nontraumatic subarachnoid hemorrhage affecting unspecified side | At least 1 IP or 2 OP claims | Any dx on claim |
| I69.061 | Other paralytic syndrome following nontraumatic subarachnoid hemorrhage affecting right dominant side | At least 1 IP or 2 OP claims | Any dx on claim |
| I69.062 | Other paralytic syndrome following nontraumatic subarachnoid hemorrhage affecting left dominant side | At least 1 IP or 2 OP claims | Any dx on claim |
| I69.063 | Other paralytic syndrome following nontraumatic subarachnoid hemorrhage affecting right non-dominant side | At least 1 IP or 2 OP claims | Any dx on claim |
| I69.064 | Other paralytic syndrome following nontraumatic subarachnoid hemorrhage affecting left non-dominant side | At least 1 IP or 2 OP claims | Any dx on claim |
| I69.065 | Other paralytic syndrome following nontraumatic subarachnoid hemorrhage, bilateral | At least 1 IP or 2 OP claims | Any dx on claim |
| I69.069 | Other paralytic syndrome following nontraumatic subarachnoid hemorrhage affecting unspecified side | At least 1 IP or 2 OP claims | Any dx on claim |
| I69.090 | Apraxia following nontraumatic subarachnoid hemorrhage | At least 1 IP or 2 OP claims | Any dx on claim |
| I69.091 | Dysphagia following nontraumatic subarachnoid hemorrhage | At least 1 IP or 2 OP claims | Any dx on claim |
| I69.092 | Facial weakness following nontraumatic subarachnoid hemorrhage | At least 1 IP or 2 OP claims | Any dx on claim |
| I69.093 | Ataxia following nontraumatic subarachnoid hemorrhage | At least 1 IP or 2 OP claims | Any dx on claim |
| I69.098 | Other sequelae following nontraumatic subarachnoid hemorrhage | At least 1 IP or 2 OP claims | Any dx on claim |
| I69.10 | Unspecified sequelae of nontraumatic intracerebral hemorrhage | At least 1 IP or 2 OP claims | Any dx on claim |
| I69.11 | Cognitive deficits following nontraumatic intracerebral hemorrhage | At least 1 IP or 2 OP claims | Any dx on claim |
| I69.110 | Attention and concentration deficit following nontraumatic intracerebral hemorrhage | At least 1 IP or 2 OP claims | Any dx on claim |
| I69.111 | Memory deficit following nontraumatic intracerebral hemorrhage | At least 1 IP or 2 OP claims | Any dx on claim |
| I69.112 | Visuospatial deficit and spatial neglect following nontraumatic intracerebral hemorrhage | At least 1 IP or 2 OP claims | Any dx on claim |
| I69.113 | Psychomotor deficit following nontraumatic intracerebral hemorrhage | At least 1 IP or 2 OP claims | Any dx on claim |
| I69.114 | Frontal lobe and executive function deficit following nontraumatic intracerebral hemorrhage | At least 1 IP or 2 OP claims | Any dx on claim |
| I69.115 | Cognitive social or emotional deficit following nontraumatic intracerebral hemorrhage | At least 1 IP or 2 OP claims | Any dx on claim |
| I69.118 | Other symptoms and signs involving cognitive functions following nontraumatic intracerebral hemorrhage | At least 1 IP or 2 OP claims | Any dx on claim |
| I69.119 | Unspecified symptoms and signs involving cognitive functions following nontraumatic intracerebral hemorrhage | At least 1 IP or 2 OP claims | Any dx on claim |
| I69.120 | Aphasia following nontraumatic intracerebral hemorrhage | At least 1 IP or 2 OP claims | Any dx on claim |
| I69.121 | Dysphasia following nontraumatic intracerebral hemorrhage | At least 1 IP or 2 OP claims | Any dx on claim |
| I69.122 | Dysarthria following nontraumatic intracerebral hemorrhage | At least 1 IP or 2 OP claims | Any dx on claim |
| I69.123 | Fluency disorder following nontraumatic intracerebral hemorrhage | At least 1 IP or 2 OP claims | Any dx on claim |
| I69.128 | Other speech and language deficits following nontraumatic intracerebral hemorrhage | At least 1 IP or 2 OP claims | Any dx on claim |
| I69.131 | Monoplegia of upper limb following nontraumatic intracerebral hemorrhage affecting right dominant side | At least 1 IP or 2 OP claims | Any dx on claim |
| I69.132 | Monoplegia of upper limb following nontraumatic intracerebral hemorrhage affecting left dominant side | At least 1 IP or 2 OP claims | Any dx on claim |
| I69.133 | Monoplegia of upper limb following nontraumatic intracerebral hemorrhage affecting right non-dominant side | At least 1 IP or 2 OP claims | Any dx on claim |
| I69.134 | Monoplegia of upper limb following nontraumatic intracerebral hemorrhage affecting left non-dominant side | At least 1 IP or 2 OP claims | Any dx on claim |
| I69.139 | Monoplegia of upper limb following nontraumatic intracerebral hemorrhage affecting unspecified side | At least 1 IP or 2 OP claims | Any dx on claim |
| I69.141 | Monoplegia of lower limb following nontraumatic intracerebral hemorrhage affecting right dominant side | At least 1 IP or 2 OP claims | Any dx on claim |
| I69.142 | Monoplegia of lower limb following nontraumatic intracerebral hemorrhage affecting left dominant side | At least 1 IP or 2 OP claims | Any dx on claim |
| I69.143 | Monoplegia of lower limb following nontraumatic intracerebral hemorrhage affecting right non-dominant side | At least 1 IP or 2 OP claims | Any dx on claim |
| I69.144 | Monoplegia of lower limb following nontraumatic intracerebral hemorrhage affecting left non-dominant side | At least 1 IP or 2 OP claims | Any dx on claim |
| I69.149 | Monoplegia of lower limb following nontraumatic intracerebral hemorrhage affecting unspecified side | At least 1 IP or 2 OP claims | Any dx on claim |
| I69.151 | Hemiplegia and hemiparesis following nontraumatic intracerebral hemorrhage affecting right dominant side | At least 1 IP or 2 OP claims | Any dx on claim |
| I69.152 | Hemiplegia and hemiparesis following nontraumatic intracerebral hemorrhage affecting left dominant side | At least 1 IP or 2 OP claims | Any dx on claim |
| I69.153 | Hemiplegia and hemiparesis following nontraumatic intracerebral hemorrhage affecting right non-dominant side | At least 1 IP or 2 OP claims | Any dx on claim |
| I69.154 | Hemiplegia and hemiparesis following nontraumatic intracerebral hemorrhage affecting left non-dominant side | At least 1 IP or 2 OP claims | Any dx on claim |
| I69.159 | Hemiplegia and hemiparesis following nontraumatic intracerebral hemorrhage affecting unspecified side | At least 1 IP or 2 OP claims | Any dx on claim |
| I69.161 | Other paralytic syndrome following nontraumatic intracerebral hemorrhage affecting right dominant side | At least 1 IP or 2 OP claims | Any dx on claim |
| I69.162 | Other paralytic syndrome following nontraumatic intracerebral hemorrhage affecting left dominant side | At least 1 IP or 2 OP claims | Any dx on claim |
| I69.163 | Other paralytic syndrome following nontraumatic intracerebral hemorrhage affecting right non-dominant side | At least 1 IP or 2 OP claims | Any dx on claim |
| I69.164 | Other paralytic syndrome following nontraumatic intracerebral hemorrhage affecting left non-dominant side | At least 1 IP or 2 OP claims | Any dx on claim |
| I69.165 | Other paralytic syndrome following nontraumatic intracerebral hemorrhage, bilateral | At least 1 IP or 2 OP claims | Any dx on claim |
| I69.169 | Other paralytic syndrome following nontraumatic intracerebral hemorrhage affecting unspecified side | At least 1 IP or 2 OP claims | Any dx on claim |
| I69.190 | Apraxia following nontraumatic intracerebral hemorrhage | At least 1 IP or 2 OP claims | Any dx on claim |
| I69.191 | Dysphagia following nontraumatic intracerebral hemorrhage | At least 1 IP or 2 OP claims | Any dx on claim |
| I69.192 | Facial weakness following nontraumatic intracerebral hemorrhage | At least 1 IP or 2 OP claims | Any dx on claim |
| I69.193 | Ataxia following nontraumatic intracerebral hemorrhage | At least 1 IP or 2 OP claims | Any dx on claim |
| I69.198 | Other sequelae of nontraumatic intracerebral hemorrhage | At least 1 IP or 2 OP claims | Any dx on claim |
| I69.20 | Unspecified sequelae of other nontraumatic intracranial hemorrhage | At least 1 IP or 2 OP claims | Any dx on claim |
| I69.21 | Cognitive deficits following other nontraumatic intracranial hemorrhage | At least 1 IP or 2 OP claims | Any dx on claim |
| I69.210 | Attention and concentration deficit following other nontraumatic intracranial hemorrhage | At least 1 IP or 2 OP claims | Any dx on claim |
| I69.211 | Memory deficit following other nontraumatic intracranial hemorrhage | At least 1 IP or 2 OP claims | Any dx on claim |
| I69.212 | Visuospatial deficit and spatial neglect following other nontraumatic intracranial hemorrhage | At least 1 IP or 2 OP claims | Any dx on claim |
| I69.213 | Psychomotor deficit following other nontraumatic intracranial hemorrhage | At least 1 IP or 2 OP claims | Any dx on claim |
| I69.214 | Frontal lobe and executive function deficit following other nontraumatic intracranial hemorrhage | At least 1 IP or 2 OP claims | Any dx on claim |
| I69.215 | Cognitive social or emotional deficit following other nontraumatic intracranial hemorrhage | At least 1 IP or 2 OP claims | Any dx on claim |
| I69.218 | Other symptoms and signs involving cognitive functions following other nontraumatic intracranial hemorrhage | At least 1 IP or 2 OP claims | Any dx on claim |
| I69.219 | Unspecified symptoms and signs involving cognitive functions following other nontraumatic intracranial hemorrhage | At least 1 IP or 2 OP claims | Any dx on claim |
| I69.220 | Aphasia following other nontraumatic intracranial hemorrhage | At least 1 IP or 2 OP claims | Any dx on claim |
| I69.221 | Dysphasia following other nontraumatic intracranial hemorrhage | At least 1 IP or 2 OP claims | Any dx on claim |
| I69.222 | Dysarthria following other nontraumatic intracranial hemorrhage | At least 1 IP or 2 OP claims | Any dx on claim |
| I69.223 | Fluency disorder following other nontraumatic intracranial hemorrhage | At least 1 IP or 2 OP claims | Any dx on claim |
| I69.228 | Other speech and language deficits following other nontraumatic intracranial hemorrhage | At least 1 IP or 2 OP claims | Any dx on claim |
| I69.231 | Monoplegia of upper limb following other nontraumatic intracranial hemorrhage affecting right dominant side | At least 1 IP or 2 OP claims | Any dx on claim |
| I69.232 | Monoplegia of upper limb following other nontraumatic intracranial hemorrhage affecting left dominant side | At least 1 IP or 2 OP claims | Any dx on claim |
| I69.233 | Monoplegia of upper limb following other nontraumatic intracranial hemorrhage affecting right non-dominant side | At least 1 IP or 2 OP claims | Any dx on claim |
| I69.234 | Monoplegia of upper limb following other nontraumatic intracranial hemorrhage affecting left non-dominant side | At least 1 IP or 2 OP claims | Any dx on claim |
| I69.239 | Monoplegia of upper limb following other nontraumatic intracranial hemorrhage affecting unspecified side | At least 1 IP or 2 OP claims | Any dx on claim |
| I69.241 | Monoplegia of lower limb following other nontraumatic intracranial hemorrhage affecting right dominant side | At least 1 IP or 2 OP claims | Any dx on claim |
| I69.242 | Monoplegia of lower limb following other nontraumatic intracranial hemorrhage affecting left dominant side | At least 1 IP or 2 OP claims | Any dx on claim |
| I69.243 | Monoplegia of lower limb following other nontraumatic intracranial hemorrhage affecting right non-dominant side | At least 1 IP or 2 OP claims | Any dx on claim |
| I69.244 | Monoplegia of lower limb following other nontraumatic intracranial hemorrhage affecting left non-dominant side | At least 1 IP or 2 OP claims | Any dx on claim |
| I69.249 | Monoplegia of lower limb following other nontraumatic intracranial hemorrhage affecting unspecified side | At least 1 IP or 2 OP claims | Any dx on claim |
| I69.251 | Hemiplegia and hemiparesis following other nontraumatic intracranial hemorrhage affecting right dominant side | At least 1 IP or 2 OP claims | Any dx on claim |
| I69.252 | Hemiplegia and hemiparesis following other nontraumatic intracranial hemorrhage affecting left dominant side | At least 1 IP or 2 OP claims | Any dx on claim |
| I69.253 | Hemiplegia and hemiparesis following other nontraumatic intracranial hemorrhage affecting right non-dominant side | At least 1 IP or 2 OP claims | Any dx on claim |
| I69.254 | Hemiplegia and hemiparesis following other nontraumatic intracranial hemorrhage affecting left non-dominant side | At least 1 IP or 2 OP claims | Any dx on claim |
| I69.259 | Hemiplegia and hemiparesis following other nontraumatic intracranial hemorrhage affecting unspecified side | At least 1 IP or 2 OP claims | Any dx on claim |
| I69.261 | Other paralytic syndrome following other nontraumatic intracranial hemorrhage affecting right dominant side | At least 1 IP or 2 OP claims | Any dx on claim |
| I69.262 | Other paralytic syndrome following other nontraumatic intracranial hemorrhage affecting left dominant side | At least 1 IP or 2 OP claims | Any dx on claim |
| I69.263 | Other paralytic syndrome following other nontraumatic intracranial hemorrhage affecting right non-dominant side | At least 1 IP or 2 OP claims | Any dx on claim |
| I69.264 | Other paralytic syndrome following other nontraumatic intracranial hemorrhage affecting left non-dominant side | At least 1 IP or 2 OP claims | Any dx on claim |
| I69.265 | Other paralytic syndrome following other nontraumatic intracranial hemorrhage, bilateral | At least 1 IP or 2 OP claims | Any dx on claim |
| I69.269 | Other paralytic syndrome following other nontraumatic intracranial hemorrhage affecting unspecified side | At least 1 IP or 2 OP claims | Any dx on claim |
| I69.290 | Apraxia following other nontraumatic intracranial hemorrhage | At least 1 IP or 2 OP claims | Any dx on claim |
| I69.291 | Dysphagia following other nontraumatic intracranial hemorrhage | At least 1 IP or 2 OP claims | Any dx on claim |
| I69.292 | Facial weakness following other nontraumatic intracranial hemorrhage | At least 1 IP or 2 OP claims | Any dx on claim |
| I69.293 | Ataxia following other nontraumatic intracranial hemorrhage | At least 1 IP or 2 OP claims | Any dx on claim |
| I69.298 | Other sequelae of other nontraumatic intracranial hemorrhage | At least 1 IP or 2 OP claims | Any dx on claim |
| I69.30 | Unspecified sequelae of cerebral infarction | At least 1 IP or 2 OP claims | Any dx on claim |
| I69.31 | Cognitive deficits following cerebral infarction | At least 1 IP or 2 OP claims | Any dx on claim |
| I69.139 | Monoplegia of upper limb following nontraumatic intracerebral hemorrhage affecting unspecified side | At least 1 IP or 2 OP claims | Any dx on claim |
| I69.141 | Monoplegia of lower limb following nontraumatic intracerebral hemorrhage affecting right dominant side | At least 1 IP or 2 OP claims | Any dx on claim |
| I69.142 | Monoplegia of lower limb following nontraumatic intracerebral hemorrhage affecting left dominant side | At least 1 IP or 2 OP claims | Any dx on claim |
| I69.143 | Monoplegia of lower limb following nontraumatic intracerebral hemorrhage affecting right non-dominant side | At least 1 IP or 2 OP claims | Any dx on claim |
| I69.144 | Monoplegia of lower limb following nontraumatic intracerebral hemorrhage affecting left non-dominant side | At least 1 IP or 2 OP claims | Any dx on claim |
| I69.149 | Monoplegia of lower limb following nontraumatic intracerebral hemorrhage affecting unspecified side | At least 1 IP or 2 OP claims | Any dx on claim |
| I69.151 | Hemiplegia and hemiparesis following nontraumatic intracerebral hemorrhage affecting right dominant side | At least 1 IP or 2 OP claims | Any dx on claim |
| I69.152 | Hemiplegia and hemiparesis following nontraumatic intracerebral hemorrhage affecting left dominant side | At least 1 IP or 2 OP claims | Any dx on claim |
| I69.153 | Hemiplegia and hemiparesis following nontraumatic intracerebral hemorrhage affecting right non-dominant side | At least 1 IP or 2 OP claims | Any dx on claim |
| I69.154 | Hemiplegia and hemiparesis following nontraumatic intracerebral hemorrhage affecting left non-dominant side | At least 1 IP or 2 OP claims | Any dx on claim |
| I69.159 | Hemiplegia and hemiparesis following nontraumatic intracerebral hemorrhage affecting unspecified side | At least 1 IP or 2 OP claims | Any dx on claim |
| I69.161 | Other paralytic syndrome following nontraumatic intracerebral hemorrhage affecting right dominant side | At least 1 IP or 2 OP claims | Any dx on claim |
| I69.162 | Other paralytic syndrome following nontraumatic intracerebral hemorrhage affecting left dominant side | At least 1 IP or 2 OP claims | Any dx on claim |
| I69.163 | Other paralytic syndrome following nontraumatic intracerebral hemorrhage affecting right non-dominant side | At least 1 IP or 2 OP claims | Any dx on claim |
| I69.164 | Other paralytic syndrome following nontraumatic intracerebral hemorrhage affecting left non-dominant side | At least 1 IP or 2 OP claims | Any dx on claim |
| I69.165 | Other paralytic syndrome following nontraumatic intracerebral hemorrhage, bilateral | At least 1 IP or 2 OP claims | Any dx on claim |
| I69.169 | Other paralytic syndrome following nontraumatic intracerebral hemorrhage affecting unspecified side | At least 1 IP or 2 OP claims | Any dx on claim |
| I69.190 | Apraxia following nontraumatic intracerebral hemorrhage | At least 1 IP or 2 OP claims | Any dx on claim |
| I69.191 | Dysphagia following nontraumatic intracerebral hemorrhage | At least 1 IP or 2 OP claims | Any dx on claim |
| I69.192 | Facial weakness following nontraumatic intracerebral hemorrhage | At least 1 IP or 2 OP claims | Any dx on claim |
| I69.193 | Ataxia following nontraumatic intracerebral hemorrhage | At least 1 IP or 2 OP claims | Any dx on claim |
| I69.198 | Other sequelae of nontraumatic intracerebral hemorrhage | At least 1 IP or 2 OP claims | Any dx on claim |
| I69.20 | Unspecified sequelae of other nontraumatic intracranial hemorrhage | At least 1 IP or 2 OP claims | Any dx on claim |
| I69.21 | Cognitive deficits following other nontraumatic intracranial hemorrhage | At least 1 IP or 2 OP claims | Any dx on claim |
| I69.210 | Attention and concentration deficit following other nontraumatic intracranial hemorrhage | At least 1 IP or 2 OP claims | Any dx on claim |
| I69.211 | Memory deficit following other nontraumatic intracranial hemorrhage | At least 1 IP or 2 OP claims | Any dx on claim |
| I69.212 | Visuospatial deficit and spatial neglect following other nontraumatic intracranial hemorrhage | At least 1 IP or 2 OP claims | Any dx on claim |
| I69.213 | Psychomotor deficit following other nontraumatic intracranial hemorrhage | At least 1 IP or 2 OP claims | Any dx on claim |
| I69.214 | Frontal lobe and executive function deficit following other nontraumatic intracranial hemorrhage | At least 1 IP or 2 OP claims | Any dx on claim |
| I69.215 | Cognitive social or emotional deficit following other nontraumatic intracranial hemorrhage | At least 1 IP or 2 OP claims | Any dx on claim |
| I69.218 | Other symptoms and signs involving cognitive functions following other nontraumatic intracranial hemorrhage | At least 1 IP or 2 OP claims | Any dx on claim |
| I69.219 | Unspecified symptoms and signs involving cognitive functions following other nontraumatic intracranial hemorrhage | At least 1 IP or 2 OP claims | Any dx on claim |
| I69.220 | Aphasia following other nontraumatic intracranial hemorrhage | At least 1 IP or 2 OP claims | Any dx on claim |
| I69.221 | Dysphasia following other nontraumatic intracranial hemorrhage | At least 1 IP or 2 OP claims | Any dx on claim |
| I69.222 | Dysarthria following other nontraumatic intracranial hemorrhage | At least 1 IP or 2 OP claims | Any dx on claim |
| I69.223 | Fluency disorder following other nontraumatic intracranial hemorrhage | At least 1 IP or 2 OP claims | Any dx on claim |
| I69.228 | Other speech and language deficits following other nontraumatic intracranial hemorrhage | At least 1 IP or 2 OP claims | Any dx on claim |
| I69.231 | Monoplegia of upper limb following other nontraumatic intracranial hemorrhage affecting right dominant side | At least 1 IP or 2 OP claims | Any dx on claim |
| I69.232 | Monoplegia of upper limb following other nontraumatic intracranial hemorrhage affecting left dominant side | At least 1 IP or 2 OP claims | Any dx on claim |
| I69.233 | Monoplegia of upper limb following other nontraumatic intracranial hemorrhage affecting right non-dominant side | At least 1 IP or 2 OP claims | Any dx on claim |
| I69.234 | Monoplegia of upper limb following other nontraumatic intracranial hemorrhage affecting left non-dominant side | At least 1 IP or 2 OP claims | Any dx on claim |
| I69.239 | Monoplegia of upper limb following other nontraumatic intracranial hemorrhage affecting unspecified side | At least 1 IP or 2 OP claims | Any dx on claim |
| I69.241 | Monoplegia of lower limb following other nontraumatic intracranial hemorrhage affecting right dominant side | At least 1 IP or 2 OP claims | Any dx on claim |
| I69.242 | Monoplegia of lower limb following other nontraumatic intracranial hemorrhage affecting left dominant side | At least 1 IP or 2 OP claims | Any dx on claim |
| I69.243 | Monoplegia of lower limb following other nontraumatic intracranial hemorrhage affecting right non-dominant side | At least 1 IP or 2 OP claims | Any dx on claim |
| I69.244 | Monoplegia of lower limb following other nontraumatic intracranial hemorrhage affecting left non-dominant side | At least 1 IP or 2 OP claims | Any dx on claim |
| I69.249 | Monoplegia of lower limb following other nontraumatic intracranial hemorrhage affecting unspecified side | At least 1 IP or 2 OP claims | Any dx on claim |
| I69.251 | Hemiplegia and hemiparesis following other nontraumatic intracranial hemorrhage affecting right dominant side | At least 1 IP or 2 OP claims | Any dx on claim |
| I69.252 | Hemiplegia and hemiparesis following other nontraumatic intracranial hemorrhage affecting left dominant side | At least 1 IP or 2 OP claims | Any dx on claim |
| I69.253 | Hemiplegia and hemiparesis following other nontraumatic intracranial hemorrhage affecting right non-dominant side | At least 1 IP or 2 OP claims | Any dx on claim |
| I69.254 | Hemiplegia and hemiparesis following other nontraumatic intracranial hemorrhage affecting left non-dominant side | At least 1 IP or 2 OP claims | Any dx on claim |
| I69.259 | Hemiplegia and hemiparesis following other nontraumatic intracranial hemorrhage affecting unspecified side | At least 1 IP or 2 OP claims | Any dx on claim |
| I69.261 | Other paralytic syndrome following other nontraumatic intracranial hemorrhage affecting right dominant side | At least 1 IP or 2 OP claims | Any dx on claim |
| I69.262 | Other paralytic syndrome following other nontraumatic intracranial hemorrhage affecting left dominant side | At least 1 IP or 2 OP claims | Any dx on claim |
| I69.263 | Other paralytic syndrome following other nontraumatic intracranial hemorrhage affecting right non-dominant side | At least 1 IP or 2 OP claims | Any dx on claim |
| I69.264 | Other paralytic syndrome following other nontraumatic intracranial hemorrhage affecting left non-dominant side | At least 1 IP or 2 OP claims | Any dx on claim |
| I69.265 | Other paralytic syndrome following other nontraumatic intracranial hemorrhage, bilateral | At least 1 IP or 2 OP claims | Any dx on claim |
| I69.269 | Other paralytic syndrome following other nontraumatic intracranial hemorrhage affecting unspecified side | At least 1 IP or 2 OP claims | Any dx on claim |
| I69.290 | Apraxia following other nontraumatic intracranial hemorrhage | At least 1 IP or 2 OP claims | Any dx on claim |
| I69.291 | Dysphagia following other nontraumatic intracranial hemorrhage | At least 1 IP or 2 OP claims | Any dx on claim |
| I69.292 | Facial weakness following other nontraumatic intracranial hemorrhage | At least 1 IP or 2 OP claims | Any dx on claim |
| I69.293 | Ataxia following other nontraumatic intracranial hemorrhage | At least 1 IP or 2 OP claims | Any dx on claim |
| I69.298 | Other sequelae of other nontraumatic intracranial hemorrhage | At least 1 IP or 2 OP claims | Any dx on claim |
| I69.30 | Unspecified sequelae of cerebral infarction | At least 1 IP or 2 OP claims | Any dx on claim |
| I69.31 | Cognitive deficits following cerebral infarction | At least 1 IP or 2 OP claims | Any dx on claim |
| I69.310 | Attention and concentration deficit following cerebral infarction | At least 1 IP or 2 OP claims | Any dx on claim |
| I69.311 | Memory deficit following cerebral infarction | At least 1 IP or 2 OP claims | Any dx on claim |
| I69.312 | Visuospatial deficit and spatial neglect following cerebral infarction | At least 1 IP or 2 OP claims | Any dx on claim |
| I69.313 | Psychomotor deficit following cerebral infarction | At least 1 IP or 2 OP claims | Any dx on claim |
| I69.314 | Frontal lobe and executive function deficit following cerebral infarction | At least 1 IP or 2 OP claims | Any dx on claim |
| I69.315 | Cognitive social or emotional deficit following cerebral infarction | At least 1 IP or 2 OP claims | Any dx on claim |
| I69.318 | Other symptoms and signs involving cognitive functions following cerebral infarction | At least 1 IP or 2 OP claims | Any dx on claim |
| I69.319 | Unspecified symptoms and signs involving cognitive functions following cerebral infarction | At least 1 IP or 2 OP claims | Any dx on claim |
| I69.320 | Aphasia following cerebral infarction | At least 1 IP or 2 OP claims | Any dx on claim |
| I69.321 | Dysphasia following cerebral infarction | At least 1 IP or 2 OP claims | Any dx on claim |
| I69.322 | Dysarthria following cerebral infarction | At least 1 IP or 2 OP claims | Any dx on claim |
| I69.323 | Fluency disorder following cerebral infarction | At least 1 IP or 2 OP claims | Any dx on claim |
| I69.328 | Other speech and language deficits following cerebral infarction | At least 1 IP or 2 OP claims | Any dx on claim |
| I69.331 | Monoplegia of upper limb following cerebral infarction affecting right dominant side | At least 1 IP or 2 OP claims | Any dx on claim |
| I69.332 | Monoplegia of upper limb following cerebral infarction affecting left dominant side | At least 1 IP or 2 OP claims | Any dx on claim |
| I69.333 | Monoplegia of upper limb following cerebral infarction affecting right non-dominant side | At least 1 IP or 2 OP claims | Any dx on claim |
| I69.334 | Monoplegia of upper limb following cerebral infarction affecting left non-dominant side | At least 1 IP or 2 OP claims | Any dx on claim |
| I69.339 | Monoplegia of upper limb following cerebral infarction affecting unspecified side | At least 1 IP or 2 OP claims | Any dx on claim |
| I69.341 | Monoplegia of lower limb following cerebral infarction affecting right dominant side | At least 1 IP or 2 OP claims | Any dx on claim |
| I69.342 | Monoplegia of lower limb following cerebral infarction affecting left dominant side | At least 1 IP or 2 OP claims | Any dx on claim |
| I69.343 | Monoplegia of lower limb following cerebral infarction affecting right non-dominant side | At least 1 IP or 2 OP claims | Any dx on claim |
| I69.344 | Monoplegia of lower limb following cerebral infarction affecting left non-dominant side | At least 1 IP or 2 OP claims | Any dx on claim |
| I69.349 | Monoplegia of lower limb following cerebral infarction affecting unspecified side | At least 1 IP or 2 OP claims | Any dx on claim |
| I69.351 | Hemiplegia and hemiparesis following cerebral infarction affecting right dominant side | At least 1 IP or 2 OP claims | Any dx on claim |
| I69.352 | Hemiplegia and hemiparesis following cerebral infarction affecting left dominant side | At least 1 IP or 2 OP claims | Any dx on claim |
| I69.353 | Hemiplegia and hemiparesis following cerebral infarction affecting right non-dominant side | At least 1 IP or 2 OP claims | Any dx on claim |
| I69.354 | Hemiplegia and hemiparesis following cerebral infarction affecting left non-dominant side | At least 1 IP or 2 OP claims | Any dx on claim |
| I69.359 | Hemiplegia and hemiparesis following cerebral infarction affecting unspecified side | At least 1 IP or 2 OP claims | Any dx on claim |
| I69.361 | Other paralytic syndrome following cerebral infarction affecting right dominant side | At least 1 IP or 2 OP claims | Any dx on claim |
| I69.362 | Other paralytic syndrome following cerebral infarction affecting left dominant side | At least 1 IP or 2 OP claims | Any dx on claim |
| I69.363 | Other paralytic syndrome following cerebral infarction affecting right non-dominant side | At least 1 IP or 2 OP claims | Any dx on claim |
| I69.364 | Other paralytic syndrome following cerebral infarction affecting left non-dominant side | At least 1 IP or 2 OP claims | Any dx on claim |
| I69.365 | Other paralytic syndrome following cerebral infarction, bilateral | At least 1 IP or 2 OP claims | Any dx on claim |
| I69.369 | Other paralytic syndrome following cerebral infarction affecting unspecified side | At least 1 IP or 2 OP claims | Any dx on claim |
| I69.390 | Apraxia following cerebral infarction | At least 1 IP or 2 OP claims | Any dx on claim |
| I69.391 | Dysphagia following cerebral infarction | At least 1 IP or 2 OP claims | Any dx on claim |
| I69.392 | Facial weakness following cerebral infarction | At least 1 IP or 2 OP claims | Any dx on claim |
| I69.393 | Ataxia following cerebral infarction | At least 1 IP or 2 OP claims | Any dx on claim |
| I69.398 | Other sequelae of cerebral infarction | At least 1 IP or 2 OP claims | Any dx on claim |
| I69.80 | Unspecified sequelae of other cerebrovascular disease | At least 1 IP or 2 OP claims | Any dx on claim |
| I69.81 | Cognitive deficits following other cerebrovascular disease | At least 1 IP or 2 OP claims | Any dx on claim |
| I69.810 | Attention and concentration deficit following other cerebrovascular disease | At least 1 IP or 2 OP claims | Any dx on claim |
| I69.811 | Memory deficit following other cerebrovascular disease | At least 1 IP or 2 OP claims | Any dx on claim |
| I69.812 | Visuospatial deficit and spatial neglect following other cerebrovascular disease | At least 1 IP or 2 OP claims | Any dx on claim |
| I69.813 | Psychomotor deficit following other cerebrovascular disease | At least 1 IP or 2 OP claims | Any dx on claim |
| I69.814 | Frontal lobe and executive function deficit following other cerebrovascular disease | At least 1 IP or 2 OP claims | Any dx on claim |
| I69.815 | Cognitive social or emotional deficit following other cerebrovascular disease | At least 1 IP or 2 OP claims | Any dx on claim |
| I69.818 | Other symptoms and signs involving cognitive functions following other cerebrovascular disease | At least 1 IP or 2 OP claims | Any dx on claim |
| I69.819 | Unspecified symptoms and signs involving cognitive functions following other cerebrovascular disease | At least 1 IP or 2 OP claims | Any dx on claim |
| I69.820 | Aphasia following other cerebrovascular disease | At least 1 IP or 2 OP claims | Any dx on claim |
| I69.821 | Dysphasia following other cerebrovascular disease | At least 1 IP or 2 OP claims | Any dx on claim |
| I69.822 | Dysarthria following other cerebrovascular disease | At least 1 IP or 2 OP claims | Any dx on claim |
| I69.823 | Fluency disorder following other cerebrovascular disease | At least 1 IP or 2 OP claims | Any dx on claim |
| I69.828 | Other speech and language deficits following other cerebrovascular disease | At least 1 IP or 2 OP claims | Any dx on claim |
| I69.831 | Monoplegia of upper limb following other cerebrovascular disease affecting right dominant side | At least 1 IP or 2 OP claims | Any dx on claim |
| I69.832 | Monoplegia of upper limb following other cerebrovascular disease affecting left dominant side | At least 1 IP or 2 OP claims | Any dx on claim |
| I69.833 | Monoplegia of upper limb following other cerebrovascular disease affecting right non-dominant side | At least 1 IP or 2 OP claims | Any dx on claim |
| I69.834 | Monoplegia of upper limb following other cerebrovascular disease affecting left non-dominant side | At least 1 IP or 2 OP claims | Any dx on claim |
| I69.839 | Monoplegia of upper limb following other cerebrovascular disease affecting unspecified side | At least 1 IP or 2 OP claims | Any dx on claim |
| I69.841 | Monoplegia of lower limb following other cerebrovascular disease affecting right dominant side | At least 1 IP or 2 OP claims | Any dx on claim |
| I69.842 | Monoplegia of lower limb following other cerebrovascular disease affecting left dominant side | At least 1 IP or 2 OP claims | Any dx on claim |
| I69.843 | Monoplegia of lower limb following other cerebrovascular disease affecting right non-dominant side | At least 1 IP or 2 OP claims | Any dx on claim |
| I69.844 | Monoplegia of lower limb following other cerebrovascular disease affecting left non-dominant side | At least 1 IP or 2 OP claims | Any dx on claim |
| I69.849 | Monoplegia of lower limb following other cerebrovascular disease affecting unspecified side | At least 1 IP or 2 OP claims | Any dx on claim |
| I69.851 | Hemiplegia and hemiparesis following other cerebrovascular disease affecting right dominant side | At least 1 IP or 2 OP claims | Any dx on claim |
| I69.852 | Hemiplegia and hemiparesis following other cerebrovascular disease affecting left dominant side | At least 1 IP or 2 OP claims | Any dx on claim |
| I69.853 | Hemiplegia and hemiparesis following other cerebrovascular disease affecting right non-dominant side | At least 1 IP or 2 OP claims | Any dx on claim |
| I69.854 | Hemiplegia and hemiparesis following other cerebrovascular disease affecting left non-dominant side | At least 1 IP or 2 OP claims | Any dx on claim |
| I69.859 | Hemiplegia and hemiparesis following other cerebrovascular disease affecting unspecified side | At least 1 IP or 2 OP claims | Any dx on claim |
| I69.861 | Other paralytic syndrome following other cerebrovascular disease affecting right dominant side | At least 1 IP or 2 OP claims | Any dx on claim |
| I69.862 | Other paralytic syndrome following other cerebrovascular disease affecting left dominant side | At least 1 IP or 2 OP claims | Any dx on claim |
| I69.863 | Other paralytic syndrome following other cerebrovascular disease affecting right non-dominant side | At least 1 IP or 2 OP claims | Any dx on claim |
| I69.864 | Other paralytic syndrome following other cerebrovascular disease affecting left non-dominant side | At least 1 IP or 2 OP claims | Any dx on claim |
| I69.865 | Other paralytic syndrome following other cerebrovascular disease, bilateral | At least 1 IP or 2 OP claims | Any dx on claim |
| I69.869 | Other paralytic syndrome following other cerebrovascular disease affecting unspecified side | At least 1 IP or 2 OP claims | Any dx on claim |
| I69.890 | Apraxia following other cerebrovascular disease | At least 1 IP or 2 OP claims | Any dx on claim |
| I69.891 | Dysphagia following other cerebrovascular disease | At least 1 IP or 2 OP claims | Any dx on claim |
| I69.892 | Facial weakness following other cerebrovascular disease | At least 1 IP or 2 OP claims | Any dx on claim |
| I69.893 | Ataxia following other cerebrovascular disease | At least 1 IP or 2 OP claims | Any dx on claim |
| I69.898 | Other sequelae of other cerebrovascular disease | At least 1 IP or 2 OP claims | Any dx on claim |
| I69.90 | Unspecified sequelae of unspecified cerebrovascular disease | At least 1 IP or 2 OP claims | Any dx on claim |
| I69.91 | Cognitive deficits following unspecified cerebrovascular disease | At least 1 IP or 2 OP claims | Any dx on claim |
| I69.910 | Attention and concentration deficit following unspecified cerebrovascular disease | At least 1 IP or 2 OP claims | Any dx on claim |
| I69.911 | Memory deficit following unspecified cerebrovascular disease | At least 1 IP or 2 OP claims | Any dx on claim |
| I69.912 | Visuospatial deficit and spatial neglect following unspecified cerebrovascular disease | At least 1 IP or 2 OP claims | Any dx on claim |
| I69.913 | Psychomotor deficit following unspecified cerebrovascular disease | At least 1 IP or 2 OP claims | Any dx on claim |
| I69.914 | Frontal lobe and executive function deficit following unspecified cerebrovascular disease | At least 1 IP or 2 OP claims | Any dx on claim |
| I69.915 | Cognitive social or emotional deficit following unspecified cerebrovascular disease | At least 1 IP or 2 OP claims | Any dx on claim |
| I69.918 | Other symptoms and signs involving cognitive functions following unspecified cerebrovascular disease | At least 1 IP or 2 OP claims | Any dx on claim |
| I69.919 | Unspecified symptoms and signs involving cognitive functions following unspecified cerebrovascular disease | At least 1 IP or 2 OP claims | Any dx on claim |
| I69.920 | Aphasia following unspecified cerebrovascular disease | At least 1 IP or 2 OP claims | Any dx on claim |
| I69.921 | Dysphasia following unspecified cerebrovascular disease | At least 1 IP or 2 OP claims | Any dx on claim |
| I69.922 | Dysarthria following unspecified cerebrovascular disease | At least 1 IP or 2 OP claims | Any dx on claim |
| I69.923 | Fluency disorder following unspecified cerebrovascular disease | At least 1 IP or 2 OP claims | Any dx on claim |
| I69.928 | Other speech and language deficits following unspecified cerebrovascular disease | At least 1 IP or 2 OP claims | Any dx on claim |
| I69.931 | Monoplegia of upper limb following unspecified cerebrovascular disease affecting right dominant side | At least 1 IP or 2 OP claims | Any dx on claim |
| I69.932 | Monoplegia of upper limb following unspecified cerebrovascular disease affecting left dominant side | At least 1 IP or 2 OP claims | Any dx on claim |
| I69.933 | Monoplegia of upper limb following unspecified cerebrovascular disease affecting right non-dominant side | At least 1 IP or 2 OP claims | Any dx on claim |
| I69.934 | Monoplegia of upper limb following unspecified cerebrovascular disease affecting left non-dominant side | At least 1 IP or 2 OP claims | Any dx on claim |
| I69.939 | Monoplegia of upper limb following unspecified cerebrovascular disease affecting unspecified side | At least 1 IP or 2 OP claims | Any dx on claim |
| I69.941 | Monoplegia of lower limb following unspecified cerebrovascular disease affecting right dominant side | At least 1 IP or 2 OP claims | Any dx on claim |
| I69.942 | Monoplegia of lower limb following unspecified cerebrovascular disease affecting left dominant side | At least 1 IP or 2 OP claims | Any dx on claim |
| I69.943 | Monoplegia of lower limb following unspecified cerebrovascular disease affecting right non-dominant side | At least 1 IP or 2 OP claims | Any dx on claim |
| I69.944 | Monoplegia of lower limb following unspecified cerebrovascular disease affecting left non-dominant side | At least 1 IP or 2 OP claims | Any dx on claim |
| I69.949 | Monoplegia of lower limb following unspecified cerebrovascular disease affecting unspecified side | At least 1 IP or 2 OP claims | Any dx on claim |
| I69.951 | Hemiplegia and hemiparesis following unspecified cerebrovascular disease affecting right dominant side | At least 1 IP or 2 OP claims | Any dx on claim |
| I69.952 | Hemiplegia and hemiparesis following unspecified cerebrovascular disease affecting left dominant side | At least 1 IP or 2 OP claims | Any dx on claim |
| I69.953 | Hemiplegia and hemiparesis following unspecified cerebrovascular disease affecting right non-dominant side | At least 1 IP or 2 OP claims | Any dx on claim |
| I69.954 | Hemiplegia and hemiparesis following unspecified cerebrovascular disease affecting left non-dominant side | At least 1 IP or 2 OP claims | Any dx on claim |
| I69.959 | Hemiplegia and hemiparesis following unspecified cerebrovascular disease affecting unspecified side | At least 1 IP or 2 OP claims | Any dx on claim |
| I69.961 | Other paralytic syndrome following unspecified cerebrovascular disease affecting right dominant side | At least 1 IP or 2 OP claims | Any dx on claim |
| I69.962 | Other paralytic syndrome following unspecified cerebrovascular disease affecting left dominant side | At least 1 IP or 2 OP claims | Any dx on claim |
| I69.963 | Other paralytic syndrome following unspecified cerebrovascular disease affecting right non-dominant side | At least 1 IP or 2 OP claims | Any dx on claim |
| I69.964 | Other paralytic syndrome following unspecified cerebrovascular disease affecting left non-dominant side | At least 1 IP or 2 OP claims | Any dx on claim |
| I69.965 | Other paralytic syndrome following unspecified cerebrovascular disease, bilateral | At least 1 IP or 2 OP claims | Any dx on claim |
| I69.969 | Other paralytic syndrome following unspecified cerebrovascular disease affecting unspecified side | At least 1 IP or 2 OP claims | Any dx on claim |
| I69.990 | Apraxia following unspecified cerebrovascular disease | At least 1 IP or 2 OP claims | Any dx on claim |
| I69.991 | Dysphagia following unspecified cerebrovascular disease | At least 1 IP or 2 OP claims | Any dx on claim |
| I69.992 | Facial weakness following unspecified cerebrovascular disease | At least 1 IP or 2 OP claims | Any dx on claim |
| I69.993 | Ataxia following unspecified cerebrovascular disease | At least 1 IP or 2 OP claims | Any dx on claim |
| I69.998 | Other sequelae following unspecified cerebrovascular disease | At least 1 IP or 2 OP claims | Any dx on claim |
| I97.810 | Intraoperative cerebrovascular infarction during cardiac surgery | At least 1 IP or 2 OP claims | Any dx on claim |
| I97.811 | Intraoperative cerebrovascular infarction during other surgery | At least 1 IP or 2 OP claims | Any dx on claim |
| I97.820 | Postprocedural cerebrovascular infarction during cardiac surgery | At least 1 IP or 2 OP claims | Any dx on claim |
| I97.821 | Postprocedural cerebrovascular infarction during other surgery | At least 1 IP or 2 OP claims | Any dx on claim |

### Appendix B: Determining Hospital-Physician Integration

We determined physicians' hospital vertical integration status by building on prior work. Specifically, we used tax identifiers, hospital-related keywords, and claims-based approaches. We further refined the keyword approach through manual checks and web searches to reduce Type I and Type II errors.

The data sources we relied on included the MDPPAS database from 2013-2021; the AHA annual survey from 2015 and 2020; and the AHRQ compendium on health systems from 2016, 2018, and 2022. MDPPAS contains each physician's primary tax identifier number (TIN) and the legal name of the organization from which the physician bills the majority of Medicare services. This is what we mean by the physician’s TIN.

Physicians could qualify as hospital integrated by several different means, which we articulate below. We determined physician integration status separately for each year.

#### Qualifying based on a TIN legal name connected to a hospital, using AHRQ Compendium

##### Overview

Hospitals integrate with physicians both directly via employment (e.g., hired directly by Kaiser Permanente) and through acquisitions of independent practices (e.g., Kaiser Permanente acquires Valley Physicians Group, LLC). We searched for both types of physicians in our data. The method described here identifies those who were most probably directly employed.

In this method, we created a list of eligible TIN legal names. We identified TIN legal names that we concluded to be hospital TINs during the entire study period. That is, we looked for the TIN names that were quite obviously linked to a hospital. Then, we took this set of TIN names to all the years of our data. For each year that a physician billed the majority of services under a TIN on that list, they qualified as vertically integrated that year.

##### Details

Many hospital systems have different TIN legal names corresponding to different departments or administrative units within the hospital system. We found that one common administrative unit for a hospital was its physician group, under which the large majority (sometimes all) of a hospital’s employed physicians billed for patient care services. For example, the University of Virginia Health System uses the TIN legal name "University of Virginia Physicians Group" for its employed physicians.

To find these hospital TIN names, we conducted a deep dive analysis on the AHRQ Compendium from 2018, roughly the midpoint of our data window. The Compendium comprises between 600 and 700 health systems nationwide and identifies a series of TIN legal names that are associated with each system (a total of about 7,000 TINs). For example, Cleveland Clinic has 14 TINs associated with it, including Cleveland Clinic Florida, Cleveland Clinic Health System - East Region, Cleveland Clinic Nevada, Fairview Hospital, Heart Rhythm Associates, The Cleveland Clinic Foundation, and others. In our list of TIN legal names that we categorized as hospital TINs, we included all those that included the term "Cleveland Clinic" - this covered nearly 90 percent of the physicians that the Compendium reports as part of Cleveland Clinic. Heart Rhythm Associates, by contrast, we did not include in this list because the purpose of this list was to identify TIN names that reliably identified hospitals at all times. The name Heart Rhythm Associates did not obviously tie the organization to Cleveland Clinic.

We expect a very low rate of Type I error from this process, i.e., unless a TIN name was clearly part of the hospital, we avoided classifying it as a hospital TIN. We were particularly careful to avoid greenlighting TINs that could have been the result of practice acquisitions (and would therefore only be hospital-integrated at certain times). For example, University of Pennsylvania Health System had an obvious favored TIN for its physicians (University of Penn Medical Group, with two-thirds of all its physicians as reported in the Compendium). The Compendium also identifies 18 other TINs that are part of the Penn system. Some of these were quite likely the result of acquisitions. For example, "Princeton Family Care" with two physicians was almost certainly the product of Penn acquiring a small primary care practice. Thus, whether looking at data from 2013 or 2021, we were confident that physicians billing the majority of their services under "University of Penn Medical Group" were integrated for purposes of our study. However, we did not make this same assumption for physicians connected to Princeton Family Care. Although the physicians of this practice appear to have been integrated as of 2018 per the AHRQ compendium, we do not have data on when the acquisition was completed. Accordingly, from 2018 onward, it is likely a safe assumption that physicians billing under the Princeton Family Care LLC TIN were hospital-integrated, but the status of such physicians prior to this date is uncertain. As such, having Princeton Family Care as one’s primary TIN name was not ipso facto qualifying as hospital integrated. We note, though, that this does not preclude the physicians connected with Princeton Family Care from qualifying as hospital-integrated via another approach, e.g., specifically the claims approach described below.

Some systems had ambiguous naming conventions and therefore required manual web searches. For example, when we examined Houston Methodist, there was no TIN that was immediately obvious as their physician group TIN. However, given that one of their TINs ("Tmh Physician Associates, PLLC") comprised nearly 80% of their physicians and Tmh was likely an acronym for Texas Methodist Houston, we were sufficiently convinced that this name was more likely to be a hospital TIN than a former practice TIN.

Altogether, we identified over 2,000 TIN legal names that we believed were hospital TINs. In any year that a majority of a physician’s services occurred under one of these, we considered that physician to be hospital-integrated.

#### Qualifying based on a TIN legal name containing specific keywords

##### Overview

Method 1 identified many hospital-based TINs, but hospital names can vary slightly over time. For this reason, we also applied a basic keyword search to each physician’s primary TIN legal name as a backup. If a physician’s primary TIN met the criteria below in any given year, they qualified as integrated in that year.

##### Details

Our review of the AHA data showed that over 80 percent of U.S. hospital names contain the keywords hospital, health system, healthsystem, health science center, health sciences center, or medical center. If a physician’s TIN legal name in MDPPAS contained one of these terms, we flagged the NPI as possibly hospital integrated. We assumed those with the word "hospital" were hospital integrated. The other terms were slightly more ambiguous, especially "medical center" or "health system." “Health system” flagged important hospital systems like Mayo Clinic Health System, but we reviewed the flagged TINs and found that a few were also urgent care clinics or mental health organizations. Thus, we checked the flagged TINs against the AHRQ compendium. If it had a match in the compendium hospital name list, it qualified as integrated; if not, we flagged it for manual web searches. We then removed TINs that were not hospital systems as determined by manual web searches. For example, the legal name "GMG Health Systems Associates, PA" is a medical group, suggested by the "PA" [professional association] suffix and verified via web search. Similarly, though it matches the basic keyword search, "You First Health Systems" is not a hospital but a mental health services organization operating in Maryland.

#### Qualifying based on a TIN legal name that matched an AHA hospital name

We compiled a list of hospital names from the AHA data (2015, 2020) and the AHRQ Compendium data (2016, 2018, 2022). We included the names of both individual hospitals and health systems. Physicians working for organizations with TIN names that matched one of these qualified as hospital-integrated.

#### Qualifying based on claims submission history

We identified, for each physician in each year, the number of services delivered in either an office or a HOPD setting, based on Medicare claims. If 75% or more of those were delivered in an HOPD, the physician qualified as hospital-integrated. We also took steps to reduce errors from this measure. For instance, solo practitioners meeting this criterion qualified as hospital integrated. However, for a TIN with multiple physicians, if one physician was identified as integrated but others connected to the same TIN were not, we flagged this TIN as a potential false positive. We thus applied corrections per the following.

Requirements for correcting a physician’s HOPD-billing-based classification of integration:

1. multiple doctors in the TIN
2. physician’s claims-based measure of integration was 75% or above, but
3. three-quarters or more of the other physicians connected to the TIN had claims-based measures under 75%

Under this set of rules, we classified all physicians connected to the TIN as independent, surmising that if three-quarters of a practice appeared to be independent, all associated physicians were likely independent.

We intentionally dropped a few ambiguous cases. Specifically, we dropped the physician-year observations where there was “disagreement” in a TIN about integration status but the TIN did not meet criterion (3) above. Over our 2013-2021 data period, this amounted to about 25,000 (out of more than 5 million) physician-year observations across all physician specialties. Given the ambiguity, we dropped these observations and our analysis did not include patients attributed to such physicians. We consider the improvement in measurement accuracy worth the negligible sample size reduction.

#### Random Sample Checks and Exclusions

After all of the above, we drew random samples of the data to check for accuracy. We iterated through our process to add exclusions based on what we learned along the way. For example, we had greenlit TIN names containing the term "Spectrum Health" because Spectrum Health is a large hospital system in southwest Michigan. However, from our sample of data we found that a few physicians were connected to TINs with the term "Spectrum Health" in the name (e.g., Full Spectrum Health PLLC, which is not a hospital). We removed the specific keyword “Spectrum Health” from the list we created in method 1, but allowed “Spectrum Health Hospitals.” These manual corrections were not common, but contributed to incremental improvements to measurement accuracy nevertheless.

#### Advantages and Limitations

Prior work has used the 75% HOPD claims threshold and the basic keyword search (hospital, health system, medical center, etc.). The approach we have outlined here uses those methods and then reduces measurement error associated with them. In particular, our detailed approach to categorizing hospital TINs reduced some important Type II errors (that is, failing to categorize as integrated physicians who in fact were). A basic keyword search would flag systems such as Mount Sinai Health System or Maimonides Medical Center, but it would fail to flag systems whose legal names do not include these terms - e.g., "Cleveland Clinic" and "Kaiser Permanente." We further found that some large systems like the University of California Health System used a wide array of TIN legal names that defied keyword searches: our manual review turned up, for example, "UNIV OF CA" and "UNIVERSITY OF CLAIFORNIA" (typo reproduced). These would have gone unnoticed by keyword searches using correctly-spelled and complete words.

Although our approach is subject to less measurement error than claims-based approaches, it has limitations as well. One limitation may be the result of hospitals or systems changing their names due to rebranding, mergers, or other events. Spectrum Health and Beaumont recently merged to become Corewell Health. This particular event fell outside of our study window but if similar events occurred during our study period, we might have missed physicians who were hired under a legal name of "Corewell Health," as we would not have recognized Corewell Health as a hospital system. Such physicians might still have qualified via the claims-based measure. Similarly, some health systems had very nonspecific names such as Memorial Hospital or Community Hospital. In such cases we had to consider the risks of both type I and type II errors. A TIN name such as "Community Physicians" could be Community Hospital's favored TIN for its employed physicians. Adding a rule allowing that phrase would reduce type II errors. However, “Community Physicians” is very ambiguous terminology. Hundreds of independent practices include the phrase "community physicians" in their legal names, which would introduce many type I errors. We therefore avoided adding ambiguous names to the approved hospital TIN list of method 1 unless we were confident either that there would be few false positives or that we could manually exclude the false positives that such a rule would generate. Last, our random draws for manual verification also helped to increase our confidence in our approach.

### Appendix C. Study Cohort Flowchart and Baseline Characteristics

Figure C1. Sample Size and Cohort Flowchart

Unique patients in APCD
(n=5,803,910)

Patients younger than 45 years old
(n=3,283,101)

Patients aged 45-99

(n=2,520,809)

Patients with <2 MCCs
(n=2,200,720)

Patients with 2+ qualifying MCCs in 2016

(n=320,089)

Patients without a dominant physician
(n=175,714)

Patients with a dominant physician during the study period

(n=144,375)

Patients attributed to physician who was already integrated in 2016 or 2017
(n=45,587)

Patients attributed to independent physician or physician who becomes integrated during study period

(n=98,788)

Patients with missing data for at least one of 6 study years
(n=21,540)

Final MCC cohort
(n=77,248)

Patients excluded because no dominant physician, compared to our study cohort:

- Less likely to have 6 complete years of data
- About 2 years younger on average
- 0.3 more MCCs, mostly driven by a much higher percentage having 5+ MCCs (13.3% versus 7.8%)
- 0.5 higher Charlson score
- 3% higher proportion of female patients
- Slightly different racial/ethnic composition (3% higher proportion of Black patients, 2% lower proportion of White patients, and 1% lower AAPI)

Patients excluded because already integrated in 2016/2017 look very similar to the excluded group above, compared to our study cohort:

- Less likely to have 6 complete years of data
- 1.8 years older on average
- 0.3 more MCCs, mostly driven by much higher percentage of having 4+ MCCs (27% versus 23%
- 0.8 higher Charlson score
- 4% higher proportion attributed to PCP
- Similar by all other demographics

Table C1. Sample Baseline Characteristics of MCC Patients after IPW, Treated by Integrated and Independent Physicians across Study Period

| **Characteristic** | **Overall***^1^* N=77,248 | **Integrated***^1^* N=7,412 | **Independent***^1^* N=69,836 | **Standardized Mean Difference** | **p-value** |
| --- | --- | --- | --- | --- | --- |
| **Number of Physicians** | 4,664 | 743 | 3,930 | -- | -- |
| **Age** | 72.28 (10.32) | 71.79 (10.36) | 72.62 (10.28) | -0.080 | <0.001 |
| **Female Sex** | 44,182 (57.2%) | 4,260 (57.5%) | 39,710 (56.9%) | 0.012 | 0.362 |
| **Race** | -- | -- | -- | 0.057 | 0.008 |
| American Indian/Alaskan Native | 52 (0.1%) | 5 (0.1%) | 46 (0.1%) | -- | -- |
| Asian American/Pacific Islander | 2,099 (2.7%) | 197 (2.7%) | 1,945 (2.8%) | -- | -- |
| Black | 12,993 (16.8%) | 1,288 (17.4%) | 11,280 (16.2%) | -- | -- |
| Other | 12,455 (16.1%) | 1,216 (16.4%) | 11,025 (15.8%) | -- | -- |
| Unknown | 975 (1.3%) | 100 (1.3%) | 813 (1.2%) | -- | -- |
| White | 48,675 (63.0%) | 4,606 (62.1%) | 44,726 (64.0%) | -- | -- |
| **Medicare-Medicaid Dual Eligibility** | 14,521 (18.8%) | 1,413 (19.1%) | 12,905 (18.5%) | 0.015 | 0.286 |
| **Payer Type** | -- | -- | -- | 0.039 | 0.333 |
| Commercial | 5,556 (7.2%) | 552 (7.4%) | 4,812 (6.9%) | -- | -- |
| Medicaid | 2,071 (2.7%) | 181 (2.4%) | 2,076 (3.0%) | -- | -- |
| Medicare | 69,621 (90.1%) | 6,680 (90.1%) | 62,948 (90.1%) | -- | -- |
| **Charlson Comorbidity Score** | 3.02 (2.36) | 2.97 (2.36) | 3.06 (2.36) | -0.039 | <0.001 |
| **MCC Count** | 2.74 (0.99) | 2.66 (0.91) | 2.79 (1.03) | -0.143 | <0.001 |
| **MCC Condition** | -- | -- | -- | -- | -- |
| Acute Myocardial Infarction | 5,717 (7.4%) | 472 (6.4%) | 6,030 (8.6%) | -0.086 | <0.001 |
| Alzheimer’s Disease and Related Disorders or Senile Dementia | 11,833 (15.3%) | 1,025 (13.8%) | 11,944 (17.1%) | -0.091 | <0.001 |
| Atrial Fibrillation | 24,031 (31.1%) | 2,193 (29.6%) | 22,993 (32.9%) | -0.072 | <0.001 |
| Chronic Kidney Disease | 46,409 (60.1%) | 4,191 (56.5%) | 44,914 (64.3%) | -0.159 | <0.001 |
| Chronic Obstructive Pulmonary Disorder and Asthma | 39,751 (51.5%) | 3,687 (49.7%) | 37,368 (53.5%) | -0.075 | <0.001 |
| Depression | 29,249 (37.9%) | 2,618 (35.3%) | 28,569 (40.9%) | -0.115 | <0.001 |
| Diabetes | 55,669 (72.1%) | 5,228 (70.5%) | 51,608 (73.9%) | -0.075 | <0.001 |
| Heart Failure | 26,499 (34.3%) | 2,313 (31.2%) | 26,544 (38.0%) | -0.143 | <0.001 |
| Stroke and Transient Ischemic Attack | 18,159 (23.5%) | 1,595 (21.5%) | 18,077 (25.9%) | -0.103 | <0.001 |
| **Clinician Specialty** | -- | -- | -- | 0.026 | 0.198 |
| Specialist | 17,072 (22.1%) | 1,664 (22.4%) | 1,5147 (21.7%) | -- | -- |
| Primary Care | 60,175 (77.9%) | 5,748 (77.6%) | 54,689 (78.3%) | -- | -- |
| **HHI Score (0 – 10,000)** | 7,142 (3,138) | 7,380 (2,972) | 6,978 (3,237) | 0.129 | <0.001 |
| **Rurality** | -- | -- | -- | 0.016 | 0.370 |
| Rural | 14,161 (18.3%) | 1,344 (18.1%) | 12,967 (18.6%) | -- | -- |
| Urban | 63,010 (81.6%) | 6,061 (81.8%) | 56,795 (81.3%) | -- | -- |
| Unknown | 77 (0.1%) | 7 (0.1%) | 74 (0.1% | -- | -- |
| **Total Annual Spending** | $21,431 ($29,004) | $19,486 ($26,873) | $22,781 ($30,323) | -0.115 | <0.001 |
| **Annual Inpatient Spending** | $5,745 ($16,609) | $5,130 ($15,139) | $6,170 ($17,543) | -0.063 | <0.001 |
| **Annual Outpatient Spending** | $5,048 ($11,962) | $4,747 ($11,455) | $5,256 ($12,298) | -0.043 | <0.001 |
| **Annual Professional Spending** | $5,087 ($6,702) | $4,398 ($6,285) | $5,565 ($6,937) | -0.176 | <0.001 |
| **Annual Pharmaceutical Spending** | $5,552 ($10,508) | $5,211 ($9,919) | $5,789 ($10,892) | -0.056 | <0.001 |

*^1^Mean (Standard Deviation [SD]); n (%)*

*^2^Pearson's Chi-squared test, which tests the association between categorical variables by comparing observed and expected frequencies; one-way ANOVA, which tests whether there is a difference in mean characteristics across the two groups*

*These descriptive statistics are based on the pre-treatment characteristics for the integrated group and corresponding years for the independent group, after inverse probability weighting.*

Table C2. Trends of Newly Integrated Clinicians from 2016-2021, by Integration Cohort

| **Integration Cohorts** | **Number of Newly Integrated NPIs (Treated Groups)** | **Number of Independent NPIs (Never-Treated Group)** |
| --- | --- | --- |
| 2016 | -- | 3,930 |
| 2017 | -- | 3,930 |
| 2018 | 175 | 3,930 |
| 2019 | 134 | 3,930 |
| 2020 | 137 | 3,930 |
| 2021 | 297 | 3,930 |
| TOTAL | 743 | 3,930 |

### Appendix D. Callaway and Sant’Anna DiD (CSDiD) Doubly Robust Spending Results

In the doubly robust CSDiD linear regression models, we found little to no evidence of differential spending after integration (Figure D1). There was no statistically significant overall difference ($9.09, 95% CI: -$789.24 to $807.43) in total annual spending per beneficiary post-integration. When assessing group-specific estimates, we found no clear pattern. The 2018 and 2020 integration cohort ATT estimates were not significant, the 2019 integration cohort’s ATT was large, positive, and marginally significant ($1,670.28, 95% CI: $-30.88 to $3,371.43), and the 2021 integration cohort’s ATT was negative and highly significant (-$1,916.57, 95% CI: -$2,966.69 to -$866.45).

When looking at the overall and group-specific ATT estimates for the secondary spending outcomes, our findings were generally aligned with the unadjusted results. Although there was no evidence of differential inpatient or professional spending after integration, we observed lower annual outpatient spending and higher annual pharmaceutical spending, primarily in the first year of integration. Of note, we observed visual and statistical evidence of differential pre-trends across many of these models, which suggests that factors other than vertical integration may explain observed differences. As a result, we are not confident that the estimated CSDiD effects are due solely to vertical integration.

Figure D1. CSDiD Forest Plot of Overall ATT Estimates and 95% CIs for All Spending Outcomes

Figure D2. CSDiD Doubly Robust Event Study Plots

Figure D3. CSDiD Doubly Robust Event Study Results (Without 2021 Integration Cohort)

Table D1. CSDiD Overall and Cohort-Specific Total Annual Spending ATT Estimates (unadjusted)

| **Group** | **Coefficient** | **Std. Error** | **z** | **p-value** | **Lower 95% CI** | **Upper 95% CI** |
| --- | --- | --- | --- | --- | --- | --- |
| Overall ATT | 9.09 | 448.99 | 0.02 | 0.984 | -870.92 | 889.11 |
| Group Average | -596.22 | 420.62 | -1.42 | 0.156 | -1420.61 | 228.17 |
| G2018 | -14.19 | 1066.32 | -0.01 | 0.989 | -2104.13 | 2075.75 |
| G2019 | 1670.28 | 1014.81 | 1.65 | 0.100 | -318.71 | 3659.27 |
| G2020 | 819.79 | 671.38 | 1.22 | 0.222 | -496.09 | 2135.67 |
| G2021 | -1916.57 | 656.27 | -2.92 | 0.003*** | -3202.84 | -630.30 |

Table D2. CSDiD Total Annual Spending Event-Time ATT Estimates (unadjusted)

| **Group** | **Coefficient** | **Std. Error** | **z** | **p-value** | **Lower 95% CI** | **Upper 95% CI** |
| --- | --- | --- | --- | --- | --- | --- |
| Pre-treatment average | -966.52 | 165.92 | -5.83 | <0.001*** | -1291.70 | -641.33 |
| Post-treatment average | -5.94 | 656.55 | -0.01 | 0.993 | -1292.74 | 1280.87 |
| 4 years prior to integration | -1391.92 | 425.00 | -3.28 | <0.001*** | -2224.91 | -558.93 |
| 3 years prior to integration | -1337.94 | 385.56 | -3.47 | <0.001*** | -2093.63 | -582.25 |
| 2 years prior to integration | -666.33 | 298.76 | -2.23 | 0.026** | -1251.90 | -80.76 |
| 1 year prior to integration | -469.88 | 377.62 | -1.24 | 0.213 | -1210.01 | 270.25 |
| First integration year | -383.25 | 430.73 | -0.89 | 0.374 | -1227.47 | 460.97 |
| One year after integration | 563.98 | 620.83 | 0.91 | 0.364 | -652.82 | 1780.78 |
| Two years after integration | 1175.59 | 1003.49 | 1.17 | 0.241 | -791.22 | 3142.40 |
| Three years after integration | -1380.07 | 1472.84 | -0.94 | 0.349 | -4266.77 | 1506.64 |

Table D3. CSDiD Overall and Cohort-Specific Total Annual Spending ATT Estimates

| **Group** | **Coefficient** | **Std. Error** | **z** | **p-value** | **Lower 95% CI** | **Upper 95% CI** |
| --- | --- | --- | --- | --- | --- | --- |
| Overall ATT | 9.09 | 407.32 | 0.02 | 0.982 | -789.24 | 807.43 |
| Group Average | -596.22 | 365.94 | -1.63 | 0.103 | -1313.45 | 121.01 |
| G2018 | -14.19 | 998.41 | -0.01 | 0.989 | -1971.04 | 1942.67 |
| G2019 | 1670.28 | 867.95 | 1.92 | 0.054* | -30.88 | 3371.43 |
| G2020 | 819.79 | 715.44 | 1.15 | 0.252 | -582.45 | 2222.03 |
| G2021 | -1916.57 | 535.78 | -3.58 | <0.001*** | -2966.69 | -866.45 |

Table D4. CSDiD Total Annual Spending Event-Time ATT Estimates

| **Group** | **Coefficient** | **Std. Error** | **z** | **p-value** | **Lower 95% CI** | **Upper 95% CI** |
| --- | --- | --- | --- | --- | --- | --- |
| Pre-treatment average | -966.52 | 128.94 | -7.5 | <0.001*** | -1219.23 | -713.80 |
| Post-treatment average | -5.94 | 595.83 | -0.01 | 0.992 | -1173.75 | 1161.87 |
| 4 years prior to integration | -1391.92 | 404.20 | -3.44 | 0.001*** | -2184.14 | -599.70 |
| 3 years prior to integration | -1337.94 | 341.59 | -3.92 | <0.001*** | -2007.45 | -668.43 |
| 2 years prior to integration | -666.33 | 320.78 | -2.08 | 0.038** | -1295.04 | -37.61 |
| 1 year prior to integration | -469.88 | 328.03 | -1.43 | 0.152 | -1112.80 | 173.05 |
| First integration year | -383.25 | 374.06 | -1.02 | 0.306 | -1116.40 | 349.90 |
| One year after integration | 563.98 | 615.60 | 0.92 | 0.360 | -642.58 | 1770.54 |
| Two years after integration | 1175.59 | 949.66 | 1.24 | 0.216 | -685.71 | 3036.89 |
| Three years after integration | -1380.07 | 1355.69 | -1.02 | 0.309 | -4037.17 | 1277.03 |

Table D5. CSDiD Overall and Cohort-Specific Total Annual Spending ATT Estimates (excluding 2021 Integration Cohort)

| **Group** | **Coefficient** | **Std. Error** | **z** | **p-value** | **Lower 95% CI** | **Upper 95% CI** |
| --- | --- | --- | --- | --- | --- | --- |
| Overall ATT | 737.80 | 521.33 | 1.42 | 0.157 | -283.99 | 1759.58 |
| Group Average | 820.13 | 488.17 | 1.68 | 0.093* | -136.67 | 1776.93 |
| G2018 | -14.19 | 998.41 | -0.01 | 0.989 | -1971.04 | 1942.67 |
| G2019 | 1670.28 | 867.95 | 1.92 | 0.054* | -30.88 | 3371.43 |
| G2020 | 819.79 | 715.44 | 1.15 | 0.252 | -582.45 | 2222.03 |

Table D6. CSDiD Total Annual Spending Event-Time ATT Estimates (excluding 2021 Integration Cohort)

| **Group** | **Coefficient** | **Std. Error** | **z** | **p-value** | **Lower 95% CI** | **Upper 95% CI** |
| --- | --- | --- | --- | --- | --- | --- |
| Pre-treatment average | 138.04 | 268.35 | 0.51 | 0.607 | -387.92 | 664.00 |
| Post-treatment average | 405.26 | 627.69 | 0.65 | 0.519 | -824.99 | 1635.52 |
| 3 years prior to integration | 57.67 | 691.08 | 0.08 | 0.933 | -1296.83 | 1412.16 |
| 2 years prior to integration | -447.96 | 527.99 | -0.85 | 0.396 | -1482.80 | 586.88 |
| 1 year prior to integration | 804.42 | 470.72 | 1.71 | 0.087* | -118.17 | 1727.01 |
| First integration year | 1261.56 | 524.23 | 2.41 | 0.016** | 234.07 | 2289.04 |
| One year after integration | 563.98 | 615.60 | 0.92 | 0.36 | -642.58 | 1770.54 |
| Two years after integration | 1175.59 | 949.66 | 1.24 | 0.216 | -685.71 | 3036.89 |
| Three years after integration | -1380.07 | 1355.69 | -1.02 | 0.309 | -4037.17 | 1277.03 |

Table D7. CSDiD Overall and Cohort-Specific Annual Inpatient Spending ATT Estimates (unadjusted)

| **Group** | **Coefficient** | **Std. Error** | **z** | **p-value** | **Lower 95% CI** | **Upper 95% CI** |
| --- | --- | --- | --- | --- | --- | --- |
| Overall ATT | 13.08 | 313.08 | 0.04 | 0.967 | -600.55 | 626.71 |
| Group Average | -456.70 | 316.04 | -1.45 | 0.148 | -1076.13 | 162.73 |
| G2018 | -98.91 | 664.00 | -0.15 | 0.882 | -1400.33 | 1202.52 |
| G2019 | 1541.39 | 748.77 | 2.06 | 0.040** | 73.83 | 3008.95 |
| G2020 | 524.15 | 475.01 | 1.1 | 0.27 | -406.86 | 1455.15 |
| G2021 | -1469.15 | 511.96 | -2.87 | 0.004*** | -2472.58 | -465.73 |

Table D8. CSDiD Annual Inpatient Spending Event-Time ATT Estimates (unadjusted)

| **Group** | **Coefficient** | **Std. Error** | **z** | **p-value** | **Lower 95% CI** | **Upper 95% CI** |
| --- | --- | --- | --- | --- | --- | --- |
| Pre-treatment average | -97.55 | 84.94 | -1.15 | 0.251 | -264.03 | 68.94 |
| Post-treatment average | -54.45 | 437.76 | -0.12 | 0.901 | -912.45 | 803.56 |
| 4 years prior to integration | 54.89 | 279.85 | 0.2 | 0.844 | -493.61 | 603.39 |
| 3 years prior to integration | -224.16 | 246.14 | -0.91 | 0.362 | -706.59 | 258.27 |
| 2 years prior to integration | -222.59 | 208.01 | -1.07 | 0.285 | -630.27 | 185.10 |
| 1 year prior to integration | 1.67 | 265.84 | 0.01 | 0.995 | -519.37 | 522.72 |
| First integration year | -326.28 | 323.86 | -1.01 | 0.314 | -961.03 | 308.48 |
| One year after integration | 541.52 | 436.88 | 1.24 | 0.215 | -314.75 | 1397.80 |
| Two years after integration | 1117.08 | 761.35 | 1.47 | 0.142 | -375.13 | 2609.29 |
| Three years after integration | -1550.11 | 972.01 | -1.59 | 0.111 | -3455.21 | 354.99 |

Table D9. CSDiD Overall and Cohort-Specific Annual Inpatient Spending ATT Estimates

| **Group** | **Coefficient** | **Std. Error** | **z** | **p-value** | **Lower 95% CI** | **Upper 95% CI** |
| --- | --- | --- | --- | --- | --- | --- |
| Overall ATT | 13.0782 | 287.2748 | 0.05 | 0.964 | -549.9701 | 576.1265 |
| Group Average | -456.6991 | 269.9265 | -1.69 | 0.091* | -985.7453 | 72.34714 |
| G2018 | -98.9064 | 685.1572 | -0.14 | 0.885 | -1441.79 | 1243.977 |
| G2019 | 1541.389 | 619.5851 | 2.49 | 0.013** | 327.0249 | 2755.754 |
| G2020 | 524.1454 | 499.4571 | 1.05 | 0.294 | -454.7724 | 1503.063 |
| G2021 | -1469.154 | 409.192 | -3.59 | <0.001*** | -2271.155 | -667.152 |

Table D10. CSDiD Overall Annual Inpatient Spending Event-Time ATT Estimates

| **Group** | **Coefficient** | **Std. Error** | **z** | **p-value** | **Lower 95% CI** | **Upper 95% CI** |
| --- | --- | --- | --- | --- | --- | --- |
| Pre-treatment average | -97.54538 | 86.60243 | -1.13 | 0.26 | -267.283 | 72.19226 |
| Post-treatment average | -54.44652 | 414.9119 | -0.13 | 0.896 | -867.6588 | 758.7658 |
| 4 years prior to integration | 54.89259 | 287.0276 | 0.19 | 0.848 | -507.6712 | 617.4564 |
| 3 years prior to integration | -224.1606 | 232.7284 | -0.96 | 0.335 | -680.2999 | 231.9788 |
| 2 years prior to integration | -222.5856 | 219.1059 | -1.02 | 0.31 | -652.0252 | 206.854 |
| 1 year prior to integration | 1.672041 | 231.3767 | 0.01 | 0.994 | -451.8179 | 455.1619 |
| First integration year | -326.2771 | 279.1605 | -1.17 | 0.242 | -873.4217 | 220.8674 |
| One year after integration | 541.5222 | 448.0674 | 1.21 | 0.227 | -336.6737 | 1419.718 |
| Two years after integration | 1117.079 | 703.2673 | 1.59 | 0.112 | -261.2998 | 2495.458 |
| Three years after integration | -1550.11 | 982.4251 | -1.58 | 0.115 | -3475.628 | 375.4078 |

Table D11. CSDiD Overall and Cohort-Specific Annual Inpatient Spending ATT Estimates (excluding 2021 Integration Cohort)

| **Group** | **Coefficient** | **Std. Error** | **z** | **p-value** | **Lower 95% CI** | **Upper 95% CI** |
| --- | --- | --- | --- | --- | --- | --- |
| Overall ATT | 573.98 | 362.35 | 1.58 | 0.113 | -136.21 | 1284.16 |
| Group Average | 629.37 | 340.66 | 1.85 | 0.065* | -38.31 | 1297.04 |
| G2018 | -98.91 | 685.16 | -0.14 | 0.885 | -1441.79 | 1243.98 |
| G2019 | 1541.39 | 619.59 | 2.49 | 0.013** | 327.02 | 2755.75 |
| G2020 | 524.15 | 499.46 | 1.05 | 0.294 | -454.77 | 1503.06 |

Table D12 CSDiD Annual Inpatient Spending Event-Time ATT Estimates (excluding 2021 Integration Cohort)

| **Group** | **Coefficient** | **Std. Error** | **z** | **p-value** | **Lower 95% CI** | **Upper 95% CI** |
| --- | --- | --- | --- | --- | --- | --- |
| Pre-treatment average | 17.64 | 178.43 | 0.1 | 0.921 | -332.08 | 367.37 |
| Post-treatment average | 252.05 | 436.40 | 0.58 | 0.564 | -603.29 | 1107.38 |
| 3 years prior to integration | -391.18 | 446.26 | -0.88 | 0.381 | -1265.84 | 483.47 |
| 2 years prior to integration | 202.74 | 342.13 | 0.59 | 0.553 | -467.82 | 873.31 |
| 1 year prior to integration | 241.38 | 341.05 | 0.71 | 0.479 | -427.07 | 909.83 |
| First integration year | 899.69 | 379.83 | 2.37 | 0.018** | 155.23 | 1644.16 |
| One year after integration | 541.52 | 448.07 | 1.21 | 0.227 | -336.67 | 1419.72 |
| Two years after integration | 1117.08 | 703.27 | 1.59 | 0.112 | -261.30 | 2495.46 |
| Three years after integration | -1550.11 | 982.43 | -1.58 | 0.115 | -3475.63 | 375.41 |

Table D13. CSDiD Overall and Cohort-Specific Annual Outpatient Spending ATT Estimates (unadjusted)

| **Group** | **Coefficient** | **Std. Error** | **z** | **p-value** | **Lower 95% CI** | **Upper 95% CI** |
| --- | --- | --- | --- | --- | --- | --- |
| Overall ATT | -297.41 | 142.86 | -2.08 | 0.037** | -577.40 | -17.41 |
| Group Average | -369.31 | 132.12 | -2.8 | 0.005*** | -628.26 | -110.36 |
| G2018 | -292.53 | 321.40 | -0.91 | 0.363 | -922.47 | 337.41 |
| G2019 | -144.78 | 323.89 | -0.45 | 0.655 | -779.59 | 490.03 |
| G2020 | -160.30 | 278.85 | -0.57 | 0.565 | -706.83 | 386.22 |
| G2021 | -533.63 | 192.71 | -2.77 | 0.006*** | -911.33 | -155.93 |

Table D14. CSDiD Annual Outpatient Spending Event-Time ATT Estimates (unadjusted)

| **Group** | **Coefficient** | **Std. Error** | **z** | **p-value** | **Lower 95% CI** | **Upper 95% CI** |
| --- | --- | --- | --- | --- | --- | --- |
| Pre-treatment average | 113.78 | 57.50 | 1.98 | 0.048** | 1.07 | 226.48 |
| Post-treatment average | -293.45 | 211.57 | -1.39 | 0.165 | -708.13 | 121.23 |
| 4 years prior to integration | 312.26 | 171.53 | 1.82 | 0.069* | -23.93 | 648.45 |
| 3 years prior to integration | 112.04 | 144.82 | 0.77 | 0.439 | -171.79 | 395.88 |
| 2 years prior to integration | -156.43 | 127.88 | -1.22 | 0.221 | -407.07 | 94.21 |
| 1 year prior to integration | 187.23 | 132.64 | 1.41 | 0.158 | -72.74 | 447.19 |
| First integration year | -277.34 | 139.15 | -1.99 | 0.046** | -550.06 | -4.61 |
| One year after integration | -343.68 | 219.13 | -1.57 | 0.117 | -773.17 | 85.81 |
| Two years after integration | -323.19 | 291.30 | -1.11 | 0.267 | -894.12 | 247.74 |
| Three years after integration | -229.60 | 524.09 | -0.44 | 0.661 | -1256.80 | 797.60 |

Table D15. CSDiD Overall and Cohort-Specific Annual Outpatient Spending ATT Estimates

| **Group** | **Coefficient** | **Std. Error** | **z** | **p-value** | **Lower 95% CI** | **Upper 95% CI** |
| --- | --- | --- | --- | --- | --- | --- |
| Overall ATT | -297.41 | 148.62 | -2.00 | 0.045** | -588.70 | -6.11 |
| Group Average | -369.31 | 134.06 | -2.75 | 0.006*** | -632.07 | -106.56 |
| G2018 | -292.53 | 348.10 | -0.84 | 0.401 | -974.79 | 389.74 |
| G2019 | -144.78 | 337.42 | -0.43 | 0.668 | -806.11 | 516.55 |
| G2020 | -160.30 | 278.44 | -0.58 | 0.565 | -706.04 | 385.43 |
| G2021 | -533.63 | 193.18 | -2.76 | 0.006*** | -912.26 | -155.01 |

Table D16. CSDiD Annual Outpatient Spending Event-Time ATT Estimates

| **Group** | **Coefficient** | **Std. Error** | **z** | **p-value** | **Lower 95% CI** | **Upper 95% CI** |
| --- | --- | --- | --- | --- | --- | --- |
| Pre-treatment average | 113.78 | 52.14 | 2.18 | 0.029** | 11.58 | 215.97 |
| Post-treatment average | -293.45 | 214.39 | -1.37 | 0.171 | -713.66 | 126.75 |
| 4 years prior to integration | 312.26 | 156.82 | 1.99 | 0.046** | 4.90 | 619.62 |
| 3 years prior to integration | 112.04 | 144.45 | 0.78 | 0.438 | -171.07 | 395.15 |
| 2 years prior to integration | -156.43 | 131.99 | -1.19 | 0.236 | -415.12 | 102.27 |
| 1 year prior to integration | 187.23 | 123.78 | 1.51 | 0.130 | -55.38 | 429.83 |
| First integration year | -277.34 | 138.10 | -2.01 | 0.045** | -548.01 | -6.67 |
| One year after integration | -343.68 | 215.43 | -1.60 | 0.111 | -765.91 | 78.55 |
| Two years after integration | -323.19 | 323.40 | -1.00 | 0.318 | -957.05 | 310.67 |
| Three years after integration | -229.60 | 486.04 | -0.47 | 0.637 | -1182.23 | 723.03 |

Table D17. CSDiD Overall and Cohort-Specific Annual Outpatient Spending ATT Estimates (excluding 2021 Integration Cohort)

| **Group** | **Coefficient** | **Std. Error** | **z** | **p-value** | **Lower 95% CI** | **Upper 95% CI** |
| --- | --- | --- | --- | --- | --- | --- |
| Overall ATT | 573.98 | 362.35 | 1.58 | 0.113 | -136.21 | 1284.16 |
| Group Average | 629.37 | 340.66 | 1.85 | 0.065* | -38.31 | 1297.04 |
| G2018 | -98.91 | 685.16 | -0.14 | 0.885 | -1441.79 | 1243.98 |
| G2019 | 1541.39 | 619.59 | 2.49 | 0.013** | 327.02 | 2755.75 |
| G2020 | 524.15 | 499.46 | 1.05 | 0.294 | -454.77 | 1503.06 |

Table D18. CSDiD Annual Outpatient Spending Event-Time ATT Estimates (excluding 2021 Integration Cohort)

| **Group** | **Coefficient** | **Std. Error** | **z** | **p-value** | **Lower 95% CI** | **Upper 95% CI** |
| --- | --- | --- | --- | --- | --- | --- |
| Pre-treatment average | 17.64 | 178.43 | 0.10 | 0.921 | -332.08 | 367.37 |
| Post-treatment average | 252.05 | 436.40 | 0.58 | 0.564 | -603.29 | 1107.38 |
| 3 years prior to integration | -391.18 | 446.26 | -0.88 | 0.381 | -1265.84 | 483.47 |
| 2 years prior to integration | 202.74 | 342.13 | 0.59 | 0.553 | -467.82 | 873.31 |
| 1 year prior to integration | 241.38 | 341.05 | 0.71 | 0.479 | -427.07 | 909.83 |
| First integration year | 899.69 | 379.83 | 2.37 | 0.018** | 155.23 | 1644.16 |
| One year after integration | 541.52 | 448.07 | 1.21 | 0.227 | -336.67 | 1419.72 |
| Two years after integration | 1117.08 | 703.27 | 1.59 | 0.112 | -261.30 | 2495.46 |
| Three years after integration | -1550.11 | 982.43 | -1.58 | 0.115 | -3475.63 | 375.41 |

Table D19. CSDiD Overall and Cohort-Specific Annual Professional Spending ATT Estimates (unadjusted)

| **Group** | **Coefficient** | **Std. Error** | **z** | **p-value** | **Lower 95% CI** | **Upper 95% CI** |
| --- | --- | --- | --- | --- | --- | --- |
| Overall ATT | -24.70 | 82.99 | -0.3 | 0.766 | -187.36 | 137.96 |
| Group Average | -111.94 | 65.74 | -1.7 | 0.089* | -240.79 | 16.90 |
| G2018 | 67.40 | 216.17 | 0.31 | 0.755 | -356.29 | 491.09 |
| G2019 | 169.52 | 197.09 | 0.86 | 0.390 | -216.78 | 555.82 |
| G2020 | -32.05 | 117.99 | -0.27 | 0.786 | -263.30 | 199.19 |
| G2021 | -264.03 | 88.06 | -3 | 0.003*** | -436.62 | -91.43 |

Table D20. CSDiD Annual Professional Spending Event-Time ATT Estimates (unadjusted)

| **Group** | **Coefficient** | **Std. Error** | **z** | **p-value** | **Lower 95% CI** | **Upper 95% CI** |
| --- | --- | --- | --- | --- | --- | --- |
| Pre-treatment average | -476.24 | 50.37 | -9.45 | <0.001*** | -574.96 | -377.52 |
| Post-treatment average | 27.19 | 132.29 | 0.21 | 0.837 | -232.10 | 286.47 |
| 4 years prior to integration | -1799.99 | 182.11 | -9.88 | <0.001*** | -2156.92 | -1443.05 |
| 3 years prior to integration | -156.41 | 58.96 | -2.65 | 0.008*** | -271.97 | -40.86 |
| 2 years prior to integration | 56.71 | 57.35 | 0.99 | 0.323 | -55.71 | 169.12 |
| 1 year prior to integration | -5.27 | 60.53 | -0.09 | 0.931 | -123.91 | 113.37 |
| First integration year | -105.01 | 63.19 | -1.66 | 0.097* | -228.86 | 18.84 |
| One year after integration | 38.17 | 119.97 | 0.32 | 0.75 | -196.98 | 273.32 |
| Two years after integration | 123.07 | 191.60 | 0.64 | 0.521 | -252.46 | 498.59 |
| Three years after integration | 52.52 | 295.57 | 0.18 | 0.859 | -526.79 | 631.82 |

Table D21. CSDiD Overall and Cohort-Specific Annual Professional Services Spending ATT Estimates

| **Group** | **Coefficient** | **Std. Error** | **z** | **p-value** | **Lower 95% CI** | **Upper 95% CI** |
| --- | --- | --- | --- | --- | --- | --- |
| Overall ATT | -24.70 | 72.63 | -0.34 | 0.734 | -167.05 | 117.65 |
| Group Average | -111.94 | 61.87 | -1.81 | 0.070* | -233.21 | 9.32 |
| G2018 | 67.40 | 176.34 | 0.38 | 0.702 | -278.23 | 413.03 |
| G2019 | 169.52 | 154.72 | 1.10 | 0.273 | -133.73 | 472.77 |
| G2020 | -32.05 | 143.23 | -0.22 | 0.823 | -312.77 | 248.66 |
| G2021 | -264.03 | 82.81 | -3.19 | 0.001*** | -426.33 | -101.72 |

Table D22. CSDiD Annual Professional Services Spending Event-Time ATT Estimates

| **Group** | **Coefficient** | **Std. Error** | **z** | **p-value** | **Lower 95% CI** | **Upper 95% CI** |
| --- | --- | --- | --- | --- | --- | --- |
| Pre-treatment average | -476.24 | 28.48 | -16.72 | <0.001*** | -532.06 | -420.42 |
| Post-treatment average | 27.19 | 110.26 | 0.25 | 0.805 | -188.92 | 243.29 |
| 4 years prior to integration | -1799.99 | 92.55 | -19.45 | <0.001*** | -1981.39 | -1618.59 |
| 3 years prior to integration | -156.41 | 56.87 | -2.75 | 0.006*** | -267.88 | -44.95 |
| 2 years prior to integration | 56.71 | 58.37 | 0.97 | 0.331 | -57.70 | 171.12 |
| 1 year prior to integration | -5.27 | 57.55 | -0.09 | 0.927 | -118.07 | 107.52 |
| First integration year | -105.01 | 61.87 | -1.70 | 0.090* | -226.28 | 16.26 |
| One year after integration | 38.17 | 113.18 | 0.34 | 0.736 | -183.65 | 259.99 |
| Two years after integration | 123.07 | 163.78 | 0.75 | 0.452 | -197.93 | 444.07 |
| Three years after integration | 52.52 | 250.38 | 0.21 | 0.834 | -438.23 | 543.26 |

Table D23. CSDiD Overall and Cohort-Specific Annual Professional Services Spending ATT Estimates (excluding 2021 Integration Cohort)

| **Group** | **Coefficient** | **Std. Error** | **z** | **p-value** | **Lower 95% CI** | **Upper 95% CI** |
| --- | --- | --- | --- | --- | --- | --- |
| Overall ATT | 65.87 | 94.81 | 0.69 | 0.487 | -119.95 | 251.69 |
| Group Average | 51.20 | 91.63 | 0.56 | 0.576 | -128.39 | 230.78 |
| G2018 | 67.40 | 176.34 | 0.38 | 0.702 | -278.23 | 413.03 |
| G2019 | 169.52 | 154.72 | 1.10 | 0.273 | -133.73 | 472.77 |
| G2020 | -32.05 | 143.23 | -0.22 | 0.823 | -312.77 | 248.66 |

Table D24. CSDiD Annual Professional Services Spending Event-Time ATT Estimates (excluding 2021 Integration Cohort)

| **Group** | **Coefficient** | **Std. Error** | **z** | **p-value** | **Lower 95% CI** | **Upper 95% CI** |
| --- | --- | --- | --- | --- | --- | --- |
| Pre-treatment average | 100.85 | 49.19 | 2.05 | 0.040** | 4.44 | 197.26 |
| Post-treatment average | 69.83 | 115.39 | 0.61 | 0.545 | -156.33 | 295.99 |
| 3 years prior to integration | 92.90 | 115.33 | 0.81 | 0.421 | -133.14 | 318.94 |
| 2 years prior to integration | 128.04 | 106.89 | 1.20 | 0.231 | -81.46 | 337.55 |
| 1 year prior to integration | 81.60 | 87.19 | 0.94 | 0.349 | -89.29 | 252.48 |
| First integration year | 65.57 | 93.42 | 0.70 | 0.483 | -117.53 | 248.68 |
| One year after integration | 38.17 | 113.18 | 0.34 | 0.736 | -183.65 | 259.99 |
| Two years after integration | 123.07 | 163.78 | 0.75 | 0.452 | -197.93 | 444.07 |
| Three years after integration | 52.52 | 250.38 | 0.21 | 0.834 | -438.23 | 543.26 |

Table D25. CSDiD Overall and Cohort-Specific Annual Pharmaceutical Spending ATT Estimates (unadjusted)

| **Group** | **Coefficient** | **Std. Error** | **z** | **p-value** | **Lower 95% CI** | **Upper 95% CI** |
| --- | --- | --- | --- | --- | --- | --- |
| Overall ATT | 318.12 | 126.85 | 2.51 | 0.012** | 69.49 | 566.74 |
| Group Average | 341.74 | 93.22 | 3.67 | <0.001*** | 159.03 | 524.44 |
| G2018 | 309.85 | 346.59 | 0.89 | 0.371 | -369.47 | 989.16 |
| G2019 | 104.15 | 238.23 | 0.44 | 0.662 | -362.78 | 571.07 |
| G2020 | 488.00 | 212.75 | 2.29 | 0.022** | 71.02 | 904.97 |
| G2021 | 350.24 | 110.22 | 3.18 | 0.001*** | 134.22 | 566.26 |

Table D26. CSDiD Annual Pharmaceutical Spending Event-Time ATT Estimates (unadjusted)

| **Group** | **Coefficient** | **Std. Error** | **z** | **p-value** | **Lower 95% CI** | **Upper 95% CI** |
| --- | --- | --- | --- | --- | --- | --- |
| Pre-treatment average | -506.50 | 65.49 | -7.73 | <0.001*** | -634.86 | -378.15 |
| Post-treatment average | 314.77 | 194.58 | 1.62 | 0.106 | -66.59 | 696.14 |
| 4 years prior to integration | 40.92 | 103.09 | 0.4 | 0.691 | -161.13 | 242.97 |
| 3 years prior to integration | -1069.41 | 155.13 | -6.89 | <0.001*** | -1373.45 | -765.37 |
| 2 years prior to integration | -344.02 | 107.21 | -3.21 | 0.001 | -554.15 | -133.89 |
| 1 year prior to integration | -653.50 | 134.24 | -4.87 | <0.001*** | -916.61 | -390.40 |
| First integration year | 325.37 | 94.41 | 3.45 | 0.001*** | 140.33 | 510.41 |
| One year after integration | 327.97 | 178.08 | 1.84 | 0.066* | -21.06 | 677.00 |
| Two years after integration | 258.63 | 241.79 | 1.07 | 0.285 | -215.28 | 732.54 |
| Three years after integration | 347.12 | 435.70 | 0.8 | 0.426 | -506.82 | 1201.07 |

Table D27. CSDiD Overall and Cohort-Specific Annual Pharmaceutical Spending ATT Estimates

| **Group** | **Coefficient** | **Std. Error** | **z** | **p-value** | **Lower 95% CI** | **Upper 95% CI** |
| --- | --- | --- | --- | --- | --- | --- |
| Overall ATT | 318.12 | 119.70 | 2.66 | 0.008*** | 83.51 | 552.72 |
| Group Average | 341.74 | 90.93 | 3.76 | <0.001*** | 163.52 | 519.96 |
| G2018 | 309.85 | 315.04 | 0.98 | 0.325 | -307.62 | 927.32 |
| G2019 | 104.15 | 255.19 | 0.41 | 0.683 | -396.02 | 604.31 |
| G2020 | 488.00 | 208.84 | 2.34 | 0.019** | 78.68 | 897.32 |
| G2021 | 350.24 | 109.35 | 3.20 | 0.001*** | 135.93 | 564.55 |

Table D28. CSDiD Annual Pharmaceutical Spending Event-Time ATT Estimates

| **Group** | **Coefficient** | **Std. Error** | **z** | **p-value** | **Lower 95% CI** | **Upper 95% CI** |
| --- | --- | --- | --- | --- | --- | --- |
| Pre-treatment average | -506.50 | 38.25 | -13.24 | <0.001*** | -581.47 | -431.54 |
| Post-treatment average | 314.77 | 182.40 | 1.73 | 0.084* | -42.72 | 672.27 |
| 4 years prior to integration | 40.92 | 97.95 | 0.42 | 0.676 | -151.05 | 232.89 |
| 3 years prior to integration | -1069.41 | 103.55 | -10.33 | <0.001*** | -1272.36 | -866.46 |
| 2 years prior to integration | -344.02 | 95.59 | -3.60 | <0.001*** | -531.38 | -156.66 |
| 1 year prior to integration | -653.50 | 104.27 | -6.27 | <0.001*** | -857.87 | -449.14 |
| First integration year | 325.37 | 89.53 | 3.63 | <0.001*** | 149.89 | 500.86 |
| One year after integration | 327.97 | 174.73 | 1.88 | 0.061* | -14.50 | 670.44 |
| Two years after integration | 258.63 | 253.48 | 1.02 | 0.308 | -238.18 | 755.44 |
| Three years after integration | 347.12 | 399.38 | 0.87 | 0.385 | -435.66 | 1129.90 |

Table D29. CSDiD Overall and Cohort-Specific Annual Pharmaceutical Spending ATT Estimates (excluding 2021 Integration Cohort)

| **Group** | **Coefficient** | **Std. Error** | **z** | **p-value** | **Lower 95% CI** | **Upper 95% CI** |
| --- | --- | --- | --- | --- | --- | --- |
| Overall ATT | 305.96 | 159.50 | 1.92 | 0.055* | -6.65 | 618.58 |
| Group Average | 332.61 | 146.60 | 2.27 | 0.023** | 45.29 | 619.94 |
| G2018 | 309.85 | 315.04 | 0.98 | 0.325 | -307.62 | 927.32 |
| G2019 | 104.15 | 255.19 | 0.41 | 0.683 | -396.02 | 604.31 |
| G2020 | 488.00 | 208.84 | 2.34 | 0.019** | 78.68 | 897.32 |

Table D30. CSDiD Annual Pharmaceutical Spending Event-Time ATT Estimates (excluding 2021 Integration Cohort)

| **Group** | **Coefficient** | **Std. Error** | **z** | **p-value** | **Lower 95% CI** | **Upper 95% CI** |
| --- | --- | --- | --- | --- | --- | --- |
| Pre-treatment average | -17.28 | 69.31 | -0.25 | 0.803 | -153.11 | 118.56 |
| Post-treatment average | 308.10 | 192.67 | 1.6 | 0.110 | -69.52 | 685.73 |
| 3 years prior to integration | 145.95 | 129.37 | 1.13 | 0.259 | -107.62 | 399.52 |
| 2 years prior to integration | -355.32 | 155.92 | -2.28 | 0.023** | -660.91 | -49.72 |
| 1 year prior to integration | 157.54 | 127.92 | 1.23 | 0.218 | -93.18 | 408.26 |
| First integration year | 298.70 | 144.78 | 2.06 | 0.039** | 14.94 | 582.45 |
| One year after integration | 327.97 | 174.73 | 1.88 | 0.061* | -14.50 | 670.44 |
| Two years after integration | 258.63 | 253.48 | 1.02 | 0.308 | -238.18 | 755.44 |
| Three years after integration | 347.12 | 399.38 | 0.87 | 0.385 | -435.66 | 1129.90 |

### Appendix E. Synthetic Difference-in-Differences (SDiD) Spending Results

Table E1. SDiD Overall and Event Time Total Annual Spending ATT Estimates (unadjusted)

| **Group** | **Coefficient** | **Std. Error** | **Lower CI** | **Upper CI** |
| --- | --- | --- | --- | --- |
| Overall ATT | 1045.93 | 825.99 | -500.14 | 2850.53 |
| 5 years prior to integration | 56.03* | 30.74 | 2.27 | 123.60 |
| 4 years prior to integration | 60.70* | 30.21 | 0.73 | 124.10 |
| 3 years prior to integration | -12.89 | 33.88 | -91.75 | 39.36 |
| 2 years prior to integration | -3.94 | 28.31 | -67.16 | 42.49 |
| 1 year prior to integration | -6.51 | 18.01 | -42.64 | 28.36 |
| First integration year | 718.37 | 707.52 | -494.64 | 2277.33 |
| One year after integration | 1009.54 | 1043.80 | -843.86 | 3054.66 |
| Two years after integration | 2057.31 | 1753.00 | -1317.94 | 5290.04 |
| Three years after integration | 739.55 | 2034.29 | -3672.18 | 4611.53 |

Table E2. SDiD Overall and Event Time Total Annual Spending ATT Estimates

| **Group** | **Coefficient** | **Std. Error** | **Lower CI** | **Upper CI** |
| --- | --- | --- | --- | --- |
| Overall ATT | 1064.28 | 697.25 | -265.79 | 2459.07 |
| 5 years prior to integration | 40.32 | 24.81 | -6.49 | 88.67 |
| 4 years prior to integration | 34.52 | 29.33 | -24.62 | 104.56 |
| 3 years prior to integration | -0.22 | 33.23 | -68.49 | 69.29 |
| 2 years prior to integration | -12.09 | 26.62 | -63.18 | 36.10 |
| 1 year prior to integration | -3.30 | 15.80 | -40.49 | 23.78 |
| First integration year | 442.42 | 690.34 | -916.91 | 1825.78 |
| One year after integration | 1402.28 | 926.44 | -403.33 | 3192.40 |
| Two years after integration | 2005.56 | 1401.24 | -753.59 | 4495.21 |
| Three years after integration | 1172.52 | 1740.70 | -2371.53 | 4372.44 |

Table E3. SDiD Overall and Event Time Total Annual Spending ATT Estimates (without 2021 Integration Cohort)

| **Group** | **Coefficient** | **Std. Error** | **Lower CI** | **Upper CI** |
| --- | --- | --- | --- | --- |
| Overall ATT | 1750.01* | 867.71 | 207.07 | 3739.01 |
| 4 years prior to integration | 1.50 | 34.66 | -89.71 | 74.37 |
| 3 years prior to integration | 2.68 | 32.11 | -73.64 | 57.01 |
| 2 years prior to integration | -9.24 | 31.97 | -83.02 | 33.60 |
| 1 year prior to integration | 5.14 | 16.60 | -25.35 | 41.40 |
| First integration year | 2158.50* | 847.70 | 600.78 | 3858.12 |
| One year after integration | 1426.58 | 986.14 | -368.19 | 3583.36 |
| Two years after integration | 2014.39 | 1641.67 | -931.36 | 5918.12 |
| Three years after integration | 1066.47 | 1721.73 | -2274.60 | 4298.70 |

Table E4. SDiD Overall and Event Time Annual Inpatient Spending ATT Estimates (unadjusted)

| **Group** | **Coefficient** | **Std. Error** | **Lower CI** | **Upper CI** |
| --- | --- | --- | --- | --- |
| Overall ATT | 618.50 | 547.53 | -275.65 | 1781.08 |
| 5 years prior to integration | -35.86 | 30.74 | -93.36 | 20.50 |
| 4 years prior to integration | 11.58 | 21.35 | -35.29 | 51.72 |
| 3 years prior to integration | 4.40 | 25.00 | -42.20 | 51.24 |
| 2 years prior to integration | -5.69 | 18.30 | -56.12 | 22.69 |
| 1 year prior to integration | 2.74 | 14.84 | -31.48 | 29.97 |
| First integration year | 979.50 | 558.28 | -125.29 | 2161.01 |
| One year after integration | 788.05 | 654.09 | -262.16 | 2246.23 |
| Two years after integration | 211.13 | 1119.38 | -1760.62 | 2854.59 |
| Three years after integration | -617.10 | 1384.76 | -2888.54 | 2507.51 |

Table E5. SDiD Overall and Event Time Annual Inpatient Spending ATT Estimates

| **Group** | **Coefficient** | **Std. Error** | **Lower CI** | **Upper CI** |
| --- | --- | --- | --- | --- |
| Overall ATT | 696.24 | 495.23 | -221.22 | 1907.55 |
| 5 years prior to integration | -48.94* | 30.48 | -112.34 | -0.96 |
| 4 years prior to integration | -3.37 | 23.82 | -60.95 | 35.16 |
| 3 years prior to integration | 12.84 | 20.19 | -31.06 | 54.57 |
| 2 years prior to integration | -6.41 | 16.26 | -46.47 | 20.48 |
| 1 year prior to integration | 4.88 | 11.40 | -18.88 | 25.56 |
| First integration year | 813.50 | 457.64 | -8.86 | 1854.53 |
| One year after integration | 1061.30 | 672.81 | -63.11 | 2530.00 |
| Two years after integration | 362.12 | 1068.42 | -1354.64 | 2617.18 |
| Three years after integration | -136.44 | 1241.90 | -2729.46 | 2555.04 |

Table E6. SDiD Overall and Event Time Annual Inpatient Spending ATT Estimates (without 2021 Integration Cohort)

| **Group** | **Coefficient** | **Std. Error** | **Lower CI** | **Upper CI** |
| --- | --- | --- | --- | --- |
| Overall ATT | 897.58* | 549.82 | 64.72 | 2293.09 |
| 4 years prior to integration | 10.71 | 20.38 | -21.68 | 57.69 |
| 3 years prior to integration | 0.55 | 16.63 | -38.67 | 30.87 |
| 2 years prior to integration | -4.72 | 19.20 | -50.91 | 28.47 |
| 1 year prior to integration | 2.93 | 13.86 | -22.69 | 31.99 |
| First integration year | 1527.54* | 568.68 | 465.65 | 2694.56 |
| One year after integration | 1071.02* | 651.29 | 70.23 | 2575.98 |
| Two years after integration | 360.33 | 1011.83 | -1571.50 | 2548.62 |
| Three years after integration | -204.54 | 1261.68 | -2253.90 | 2604.60 |

Table E7. SDiD Overall and Event Time Annual Outpatient Spending ATT Estimates (unadjusted)

| **Group** | **Coefficient** | **Std. Error** | **Lower CI** | **Upper CI** |
| --- | --- | --- | --- | --- |
| Overall ATT | -21.62 | 258.71 | -591.08 | 469.48 |
| 5 years prior to integration | -13.33 | 15.56 | -54.45 | 2.86 |
| 4 years prior to integration | 14.87 | 13.72 | -13.55 | 41.50 |
| 3 years prior to integration | -1.25 | 12.02 | -28.07 | 19.66 |
| 2 years prior to integration | 7.39 | 11.64 | -15.76 | 30.58 |
| 1 year prior to integration | -2.38 | 5.60 | -14.03 | 7.95 |
| First integration year | -116.78 | 226.49 | -556.73 | 333.47 |
| One year after integration | -322.86 | 320.23 | -1038.22 | 291.09 |
| Two years after integration | 562.84 | 612.90 | -656.92 | 1927.82 |
| Three years after integration | 115.37 | 676.85 | -1191.72 | 1383.95 |

Table E8. SDiD Overall and Event Time Annual Outpatient Spending ATT Estimates

| **Group** | **Coefficient** | **Std. Error** | **Lower CI** | **Upper CI** |
| --- | --- | --- | --- | --- |
| Overall ATT | -53.50 | 262.98 | -542.61 | 474.58 |
| 5 years prior to integration | -8.97 | 12.96 | -45.77 | 3.57 |
| 4 years prior to integration | 11.90 | 12.27 | -13.55 | 38.00 |
| 3 years prior to integration | 2.80 | 10.32 | -22.20 | 19.15 |
| 2 years prior to integration | 6.76 | 10.31 | -12.07 | 28.72 |
| 1 year prior to integration | -3.12 | 5.03 | -12.59 | 7.08 |
| First integration year | -175.99 | 222.33 | -551.44 | 309.83 |
| One year after integration | -266.86 | 306.64 | -921.10 | 353.23 |
| Two years after integration | 480.92 | 614.66 | -693.74 | 1737.94 |
| Three years after integration | 63.81 | 585.84 | -1055.60 | 1358.98 |

Table E9. SDiD Overall and Event Time Annual Outpatient Spending ATT Estimates (without 2021 integration cohort)

| **Group** | **Coefficient** | **Std. Error** | **Lower CI** | **Upper CI** |
| --- | --- | --- | --- | --- |
| Overall ATT | 67.90 | 288.23 | -526.93 | 658.37 |
| 4 years prior to integration | 19.89 | 20.38 | -25.38 | 56.66 |
| 3 years prior to integration | 10.97 | 14.28 | -21.56 | 32.14 |
| 2 years prior to integration | 5.91 | 15.87 | -33.01 | 35.51 |
| 1 year prior to integration | -4.35 | 8.68 | -21.89 | 12.53 |
| First integration year | 105.30 | 297.51 | -420.61 | 739.83 |
| One year after integration | -254.57 | 281.48 | -897.35 | 302.55 |
| Two years after integration | 495.62 | 595.47 | -555.15 | 1771.96 |
| Three years after integration | 40.62 | 650.31 | -1380.35 | 1245.50 |

Table E10. SDiD Overall and Event Time Annual Professional Spending ATT Estimates (unadjusted)

| **Group** | **Coefficient** | **Std. Error** | **Lower CI** | **Upper CI** |
| --- | --- | --- | --- | --- |
| Overall ATT | 159.34 | 134.92 | -145.33 | 392.64 |
| 5 years prior to integration | 51.25* | 11.51 | 31.37 | 76.37 |
| 4 years prior to integration | -20.67* | 8.03 | -37.94 | -7.30 |
| 3 years prior to integration | -8.25 | 6.88 | -23.33 | 4.23 |
| 2 years prior to integration | -9.37 | 6.51 | -21.99 | 3.34 |
| 1 year prior to integration | 1.38 | 3.02 | -4.09 | 7.03 |
| First integration year | -32.69 | 123.63 | -270.21 | 213.84 |
| One year after integration | 213.25 | 183.08 | -182.40 | 600.25 |
| Two years after integration | 310.42 | 280.76 | -271.04 | 809.06 |
| Three years after integration | 566.44 | 415.60 | -163.60 | 1450.38 |

Table E11. SDiD Overall and Event Time Annual Professional Spending ATT Estimates

| **Group** | **Coefficient** | **Std. Error** | **Lower CI** | **Upper CI** |
| --- | --- | --- | --- | --- |
| Overall ATT | 167.54 | 128.28 | -107.22 | 395.91 |
| 5 years prior to integration | 50.43* | 12.51 | 29.59 | 79.22 |
| 4 years prior to integration | -27.21* | 8.40 | -46.68 | -12.95 |
| 3 years prior to integration | -5.97 | 6.62 | -20.33 | 5.13 |
| 2 years prior to integration | -10.40* | 5.50 | -22.01 | -0.18 |
| 1 year prior to integration | 1.51 | 2.83 | -4.08 | 6.49 |
| First integration year | -72.80 | 113.72 | -286.21 | 136.11 |
| One year after integration | 273.94 | 174.71 | -96.68 | 629.25 |
| Two years after integration | 313.38 | 249.27 | -189.52 | 754.29 |
| Three years after integration | 654.45 | 408.79 | -73.58 | 1455.58 |

Table E12. SDiD Overall and Event Time Annual Professional Spending ATT Estimates (without 2021 Integration Cohort)

| **Group** | **Coefficient** | **Std. Error** | **Lower CI** | **Upper CI** |
| --- | --- | --- | --- | --- |
| Overall ATT | 279.75 | 156.94 | -20.09 | 586.88 |
| 4 years prior to integration | -9.31 | 10.19 | -35.38 | 1.25 |
| 3 years prior to integration | 2.28 | 6.48 | -12.04 | 14.06 |
| 2 years prior to integration | -1.21 | 6.43 | -14.77 | 9.13 |
| 1 year prior to integration | -0.09 | 3.36 | -5.87 | 7.89 |
| First integration year | 109.47 | 142.70 | -159.72 | 376.05 |
| One year after integration | 272.45 | 195.82 | -124.48 | 656.40 |
| Two years after integration | 314.85 | 251.05 | -161.62 | 829.68 |
| Three years after integration | 670.92 | 425.18 | -182.58 | 1559.07 |

Table E13. SDiD Overall and Event Time Annual Pharmaceutical Spending ATT Estimates (unadjusted)

| **Group** | **Coefficient** | **Std. Error** | **Lower CI** | **Upper CI** |
| --- | --- | --- | --- | --- |
| Overall ATT | 408.10 | 332.47 | -206.30 | 1093.28 |
| 5 years prior to integration | 49.70 | 36.11 | -29.10 | 115.99 |
| 4 years prior to integration | 67.79* | 27.27 | 23.96 | 126.15 |
| 3 years prior to integration | 13.21 | 15.48 | -20.06 | 38.73 |
| 2 years prior to integration | 19.86 | 15.30 | -12.73 | 49.23 |
| 1 year prior to integration | -6.66 | 5.13 | -20.46 | 0.75 |
| First integration year | 154.56 | 275.42 | -458.07 | 653.79 |
| One year after integration | 399.97 | 404.31 | -330.11 | 1178.61 |
| Two years after integration | 949.21 | 516.30 | -7.66 | 2031.17 |
| Three years after integration | 545.63 | 692.88 | -745.88 | 2026.76 |

Table E14. SDiD Overall and Event Time Annual Pharmaceutical Spending ATT Estimates

| **Group** | **Coefficient** | **Std. Error** | **Lower CI** | **Upper CI** |
| --- | --- | --- | --- | --- |
| Overall ATT | 377.74 | 302.10 | -203.01 | 977.01 |
| 5 years prior to integration | 49.43 | 36.39 | -37.86 | 113.01 |
| 4 years prior to integration | 65.02* | 27.40 | 20.59 | 131.20 |
| 3 years prior to integration | 15.33 | 15.37 | -21.97 | 39.73 |
| 2 years prior to integration | 15.63 | 13.82 | -12.14 | 41.02 |
| 1 year prior to integration | 65.02* | 27.40 | 20.59 | 131.20 |
| First integration year | 128.25 | 242.90 | -351.12 | 637.86 |
| One year after integration | 386.88 | 377.57 | -341.83 | 1132.50 |
| Two years after integration | 904.20 | 498.27 | -83.11 | 1872.40 |
| Three years after integration | 480.07 | 672.24 | -901.75 | 1679.66 |

Table E15. SDiD Overall and Event Time Annual Pharmaceutical Spending ATT Estimates (without 2021 Integration Cohort)

| **Group** | **Coefficient** | **Std. Error** | **Lower CI** | **Upper CI** |
| --- | --- | --- | --- | --- |
| Overall ATT | 513.29 | 351.68 | -125.90 | 1207.87 |
| 4 years prior to integration | -13.73 | 18.67 | -61.69 | 19.55 |
| 3 years prior to integration | 2.98 | 12.06 | -24.12 | 22.56 |
| 2 years prior to integration | -6.18 | 9.63 | -26.53 | 11.70 |
| 1 year prior to integration | 2.98 | 3.25 | -3.50 | 9.47 |
| First integration year | 408.99 | 348.90 | -283.53 | 1152.52 |
| One year after integration | 379.99 | 386.75 | -361.76 | 1141.90 |
| Two years after integration | 893.70* | 473.18 | 4.73 | 1776.18 |
| Three years after integration | 448.36 | 656.57 | -774.77 | 1715.53 |

HOSPITAL-SPECIALIST INTEGRATION SENSITIVITY ANALYSIS RESULTS

Table E16. SDiD Overall and Event Time Total Annual Spending ATT Estimates (Specialist Only)

| **Group** | **Coefficient** | **Std. Error** | **Lower CI** | **Upper CI** |
| --- | --- | --- | --- | --- |
| Overall ATT | -96.14 | 1412.15 | -2863.94 | 2671.67 |
| 5 years prior to integration | 35.02 | 55.88 | -74.51 | 144.54 |
| 4 years prior to integration | -100.64 | 126.13 | -347.85 | 146.58 |
| 3 years prior to integration | -0.33 | 155.34 | -304.79 | 304.13 |
| 2 years prior to integration | 86.43 | 113.80 | -136.62 | 309.47 |
| 1 year prior to integration | -10.15 | 147.83 | -299.89 | 279.60 |
| First integration year | 769.58 | 1401.23 | -1976.83 | 3515.99 |
| One year after integration | -157.07 | 1974.41 | -4026.92 | 3712.79 |
| Two years after integration | -3234.77 | 2787.28 | -8697.83 | 2229.30 |
| Three years after integration | 1375.16 | 3901.89 | -6272.55 | 9022.87 |

Table E17. SDiD Overall and Event Time Total Annual Spending ATT Estimates (without 2021 Integration Cohort, Specialist Only)

| **Group** | **Coefficient** | **Std. Error** | **Lower CI** | **Upper CI** |
| --- | --- | --- | --- | --- |
| Overall ATT | -33.22 | 1750.81 | -3464.80 | 3398.36 |
| 4 years prior to integration | 56.55 | 143.45 | -224.62 | 337.71 |
| 3 years prior to integration | 26.56 | 101.15 | -171.70 | 224.81 |
| 2 years prior to integration | -180.00 | 138.45 | -451.36 | 91.37 |
| 1 year prior to integration | 70.24 | 59.67 | -46.70 | 187.19 |
| First integration year | 1582.61 | 1520.73 | -1398.01 | 4563.24 |
| One year after integration | -219.93 | 1720.54 | -3592.18 | 3152.32 |
| Two years after integration | -3164.19 | 3024.97 | -9093.14 | 2764.76 |
| Three years after integration | 1380.26 | 4675.09 | -7782.92 | 10543.44 |

Table E18. SDiD Overall and Event Time Annual Inpatient Spending ATT Estimates (Specialist Only)

| **Group** | **Coefficient** | **Std. Error** | **Lower CI** | **Upper CI** |
| --- | --- | --- | --- | --- |
| Overall ATT | 614.11 | 1092.06 | -1779.47 | 2996.97 |
| 5 years prior to integration | -138.04 | 131.49 | -588.73 | 51.93 |
| 4 years prior to integration | 63.16 | 76.24 | -105.17 | 208.32 |
| 3 years prior to integration | 11.47 | 119.68 | -360.18 | 181.63 |
| 2 years prior to integration | -0.44 | 76.56 | -174.14 | 133.41 |
| 1 year prior to integration | 3.19 | 57.42 | -128.25 | 120.28 |
| First integration year | 1382.80 | 974.70 | -557.36 | 3511.52 |
| One year after integration | 949.42 | 1367.31 | -1733.93 | 4154.62 |
| Two years after integration | -1572.48 | 1974.87 | -5511.82 | 2540.95 |
| Three years after integration | -286.95 | 3190.43 | -6350.98 | 6729.31 |

Table E19. SDiD Overall and Event Time Annual Inpatient Spending ATT Estimates (without 2021 Integration Cohort, Specialist Only)

| **Group** | **Coefficient** | **Std. Error** | **Lower CI** | **Upper CI** |
| --- | --- | --- | --- | --- |
| Overall ATT | 309.26 | 1399.10 | -2411.75 | 3957.31 |
| 4 years prior to integration | 47.61 | 99.10 | -190.04 | 202.06 |
| 3 years prior to integration | 17.07 | 65.63 | -124.89 | 115.80 |
| 2 years prior to integration | -50.44 | 89.63 | -360.87 | 72.22 |
| 1 year prior to integration | 9.23 | 43.71 | -74.65 | 110.27 |
| First integration year | 998.13 | 1118.11 | -855.11 | 3648.89 |
| One year after integration | 960.11 | 1652.35 | -2133.57 | 4996.94 |
| Two years after integration | -1556.92 | 1936.71 | -5738.91 | 2985.64 |
| Three years after integration | -272.38 | 3508.46 | -4872.43 | 8146.92 |

Table E20. SDiD Overall and Event Time Annual Outpatient Spending ATT Estimates (Specialist Only)

| **Group** | **Coefficient** | **Std. Error** | **Lower CI** | **Upper CI** |
| --- | --- | --- | --- | --- |
| Overall ATT | -340.54 | 512.43 | -1344.90 | 661.47 |
| 5 years prior to integration | -11.96 | 44.15 | -138.60 | 53.77 |
| 4 years prior to integration | 24.02 | 48.60 | -73.75 | 131.43 |
| 3 years prior to integration | 17.29 | 52.02 | -88.76 | 142.04 |
| 2 years prior to integration | -32.85 | 40.39 | -145.60 | 35.45 |
| 1 year prior to integration | 1.16 | 16.12 | -37.45 | 31.64 |
| First integration year | -226.53 | 510.03 | -1400.10 | 682.13 |
| One year after integration | -697.73 | 640.95 | -2090.87 | 515.96 |
| Two years after integration | -343.72 | 1105.60 | -2863.78 | 1700.97 |
| Three years after integration | 140.74 | 1718.77 | -3350.11 | 3851.79 |

Table E21. SDiD Overall and Event Time Annual Outpatient Spending ATT Estimates (without 2021 Integration Cohort, Specialist Only)

| **Group** | **Coefficient** | **Std. Error** | **Lower CI** | **Upper CI** |
| --- | --- | --- | --- | --- |
| Overall ATT | -103.28 | 520.80 | -1744.06 | 772.27 |
| 4 years prior to integration | 23.93 | 63.22 | -148.45 | 173.41 |
| 3 years prior to integration | 69.44 | 47.17 | -30.82 | 181.92 |
| 2 years prior to integration | -60.89 | 47.06 | -171.50 | 12.56 |
| 1 year prior to integration | 5.99 | 18.31 | -44.60 | 40.72 |
| First integration year | 555.11 | 660.78 | -1213.80 | 1700.99 |
| One year after integration | -698.56 | 540.01 | -1748.05 | 414.07 |
| Two years after integration | -343.79 | 972.50 | -2600.28 | 1125.41 |
| Three years after integration | 141.55 | 1850.23 | -3369.92 | 3808.80 |

Table E22. SDiD Overall and Event Time Annual Professional Spending ATT Estimates (Specialist Only)

| **Group** | **Coefficient** | **Std. Error** | **Lower CI** | **Upper CI** |
| --- | --- | --- | --- | --- |
| Overall ATT | -367.23 | 284.57 | -973.62 | 209.59 |
| 5 years prior to integration | 75.94 | 54.57 | -55.33 | 193.93 |
| 4 years prior to integration | -66.34 | 40.03 | -152.42 | 0.56 |
| 3 years prior to integration | -18.90 | 30.67 | -102.78 | 30.65 |
| 2 years prior to integration | -45.46 | 27.20 | -109.87 | -5.75 |
| 1 year prior to integration | 6.26 | 11.33 | -19.72 | 26.43 |
| First integration year | -251.67 | 303.59 | -938.05 | 345.96 |
| One year after integration | -686.04 | 371.76 | -1547.56 | -24.83 |
| Two years after integration | -616.62 | 528.76 | -1847.86 | 298.58 |
| Three years after integration | 433.56 | 946.69 | -1215.22 | 3016.55 |

Table E23. SDiD Overall and Event Time Annual Professional Spending ATT Estimates (without 2021 Integration Cohort, Specialist Only)

| **Group** | **Coefficient** | **Std. Error** | **Lower CI** | **Upper CI** |
| --- | --- | --- | --- | --- |
| Overall ATT | -339.39 | 359.23 | -1152.27 | 388.23 |
| 4 years prior to integration | 13.87 | 20.38 | -32.24 | 59.26 |
| 3 years prior to integration | 1.48 | 17.96 | -41.96 | 34.43 |
| 2 years prior to integration | -31.19 | 30.21 | -105.25 | 10.62 |
| 1 year prior to integration | 4.99 | 12.37 | -22.55 | 21.27 |
| First integration year | -85.00 | 340.60 | -1185.67 | 501.96 |
| One year after integration | -687.20 | 390.64 | -1703.29 | 6.18 |
| Two years after integration | -618.76 | 651.36 | -1836.74 | 553.11 |
| Three years after integration | 431.62 | 843.85 | -1851.73 | 1818.51 |

Table E24. SDiD Overall and Event Time Annual Pharmaceutical Spending ATT Estimates (Specialist Only)

| **Group** | **Coefficient** | **Std. Error** | **Lower CI** | **Upper CI** |
| --- | --- | --- | --- | --- |
| Overall ATT | -121.32 | 745.69 | -1379.48 | 1522.29 |
| 5 years prior to integration | 72.23 | 150.02 | -225.04 | 493.18 |
| 4 years prior to integration | 61.61 | 152.52 | -310.88 | 384.59 |
| 3 years prior to integration | 13.71 | 84.47 | -210.97 | 160.56 |
| 2 years prior to integration | 15.53 | 87.59 | -182.64 | 162.21 |
| 1 year prior to integration | 1.23 | 13.75 | -38.54 | 28.95 |
| First integration year | -251.96 | 505.10 | -1251.20 | 766.98 |
| One year after integration | 196.87 | 846.31 | -1138.22 | 2216.41 |
| Two years after integration | -626.07 | 1179.07 | -3343.40 | 1370.46 |
| Three years after integration | 499.99 | 2433.71 | -4031.40 | 6048.36 |

Table E25. SDiD Overall and Event Time Annual Pharmaceutical Spending ATT Estimates (without 2021 Integration Cohort, Specialist Only)

| **Group** | **Coefficient** | **Std. Error** | **Lower CI** | **Upper CI** |
| --- | --- | --- | --- | --- |
| Overall ATT | -17.33 | 760.67 | -1765.85 | 1094.69 |
| 4 years prior to integration | -26.50 | 64.99 | -232.26 | 71.34 |
| 3 years prior to integration | -19.71 | 44.25 | -94.40 | 77.54 |
| 2 years prior to integration | -23.14 | 40.78 | -135.49 | 50.04 |
| 1 year prior to integration | 11.14 | 10.06 | -15.83 | 25.74 |
| First integration year | -35.38 | 753.27 | -1852.88 | 1176.02 |
| One year after integration | 196.02 | 730.15 | -1730.09 | 1325.13 |
| Two years after integration | -626.29 | 966.99 | -2710.59 | 1010.08 |
| Three years after integration | 498.83 | 2052.52 | -5402.59 | 4052.66 |

HOSPITAL-PCP INTEGRATION SENSITIVITY ANALYSIS RESULTS

Table E26. SDiD Overall and Event Time Total Annual Spending ATT Estimates (PCP Only)

| **Group** | **Coefficient** | **Std. Error** | **Lower CI** | **Upper CI** |
| --- | --- | --- | --- | --- |
| Overall ATT | 1455.76 | 814.28 | -140.24 | 3051.75 |
| 5 years prior to integration | 43.93 | 40.48 | -35.40 | 123.27 |
| 4 years prior to integration | 12.49 | 37.51 | -61.04 | 86.01 |
| 3 years prior to integration | -1.03 | 25.46 | -50.93 | 48.87 |
| 2 years prior to integration | -5.15 | 24.42 | -53.01 | 42.70 |
| 1 year prior to integration | -6.78 | 13.78 | -33.79 | 20.22 |
| First integration year | 297.72 | 838.60 | -1345.94 | 1941.38 |
| One year after integration | 1918.13 | 986.84 | -16.08 | 3852.34 |
| Two years after integration | 3617.36* | 1808.98 | 71.75 | 7162.96 |
| Three years after integration | 1188.28 | 1738.41 | -2219.00 | 4595.56 |

Table E27. SDiD Overall and Event Time Total Annual Spending ATT Estimates (without 2021 Integration Cohort, PCP Only)

| **Group** | **Coefficient** | **Std. Error** | **Lower CI** | **Upper CI** |
| --- | --- | --- | --- | --- |
| Overall ATT | 2335.74 | 852.55 | 664.74 | 4006.73 |
| 4 years prior to integration | -9.01 | 49.19 | -105.41 | 87.40 |
| 3 years prior to integration | -3.06 | 44.02 | -89.33 | 83.22 |
| 2 years prior to integration | 16.22 | 36.10 | -54.52 | 86.96 |
| 1 year prior to integration | -5.17 | 23.07 | -50.38 | 40.05 |
| First integration year | 2289.49 | 700.42 | 916.66 | 3662.32 |
| One year after integration | 1923.53 | 1000.02 | -36.51 | 3883.58 |
| Two years after integration | 3622.56 | 1836.53 | 22.95 | 7222.16 |
| Three years after integration | 1195.36 | 1679.33 | -2096.13 | 4486.84 |

Table E28. SDiD Overall and Event Time Annual Inpatient Spending ATT Estimates (PCP Only)

| **Group** | **Coefficient** | **Std. Error** | **Lower CI** | **Upper CI** |
| --- | --- | --- | --- | --- |
| Overall ATT | 853.46 | 497.60 | -321.18 | 1768.79 |
| 5 years prior to integration | -12.76 | 22.68 | -94.67 | 15.77 |
| 4 years prior to integration | -16.11 | 23.59 | -87.81 | 24.19 |
| 3 years prior to integration | 5.87 | 17.23 | -38.23 | 40.61 |
| 2 years prior to integration | -7.57 | 18.34 | -56.46 | 22.29 |
| 1 year prior to integration | 3.92 | 14.08 | -29.28 | 45.79 |
| First integration year | 751.75 | 577.83 | -298.93 | 1917.18 |
| One year after integration | 1124.88 | 671.35 | -311.06 | 2627.37 |
| Two years after integration | 1090.65 | 1269.61 | -2100.64 | 3716.85 |
| Three years after integration | 184.95 | 1330.28 | -2718.55 | 2881.01 |

Table E29. SDiD Overall and Event Time Annual Inpatient Spending ATT Estimates (without 2021 Integration Cohort, PCP Only)

| **Group** | **Coefficient** | **Std. Error** | **Lower CI** | **Upper CI** |
| --- | --- | --- | --- | --- |
| Overall ATT | 1185.75 | 546.76 | 57.81 | 1813.50 |
| 4 years prior to integration | 1.90 | 22.33 | -23.00 | 47.69 |
| 3 years prior to integration | -3.58 | 40.13 | -70.57 | 59.77 |
| 2 years prior to integration | 1.17 | 20.22 | -40.67 | 32.95 |
| 1 year prior to integration | 1.07 | 22.48 | -43.12 | 34.52 |
| First integration year | 1722.21 | 680.45 | 823.20 | 2663.62 |
| One year after integration | 1127.50 | 481.08 | 332.19 | 2016.92 |
| Two years after integration | 1092.35 | 1501.10 | -1456.29 | 3227.20 |
| Three years after integration | 185.21 | 1097.66 | -1909.66 | 1439.15 |

Table E30. SDiD Overall and Event Time Annual Outpatient Spending ATT Estimates (PCP Only)

| **Group** | **Coefficient** | **Std. Error** | **Lower CI** | **Upper CI** |
| --- | --- | --- | --- | --- |
| Overall ATT | -31.79 | 250.30 | -494.96 | 384.43 |
| 5 years prior to integration | -2.79 | 8.68 | -30.41 | 7.17 |
| 4 years prior to integration | 5.06 | 9.65 | -10.12 | 27.11 |
| 3 years prior to integration | -4.36 | 10.61 | -28.55 | 15.80 |
| 2 years prior to integration | 7.84 | 12.69 | -20.48 | 35.12 |
| 1 year prior to integration | -2.20 | 6.38 | -18.61 | 7.47 |
| First integration year | -308.72 | 236.04 | -841.21 | 137.99 |
| One year after integration | -150.01 | 386.91 | -949.57 | 498.57 |
| Two years after integration | 728.89 | 539.88 | -597.78 | 1557.21 |
| Three years after integration | 33.85 | 551.07 | -1092.59 | 876.44 |

Table E31. SDiD Overall and Event Time Annual Outpatient Spending ATT Estimates (without 2021 Integration Cohort, PCP Only)

| **Group** | **Coefficient** | **Std. Error** | **Lower CI** | **Upper CI** |
| --- | --- | --- | --- | --- |
| Overall ATT | 102.36 | 363.40 | -440.22 | 899.34 |
| 4 years prior to integration | 6.63 | 25.26 | -49.89 | 44.24 |
| 3 years prior to integration | -8.19 | 13.22 | -35.77 | 8.08 |
| 2 years prior to integration | 11.93 | 9.80 | -3.47 | 28.99 |
| 1 year prior to integration | -3.47 | 4.45 | -8.62 | 6.43 |
| First integration year | -72.20 | 363.93 | -614.37 | 445.23 |
| One year after integration | -147.53 | 404.33 | -882.34 | 391.09 |
| Two years after integration | 730.63 | 720.58 | -434.35 | 2267.27 |
| Three years after integration | 34.08 | 665.06 | -1282.95 | 1042.66 |

Table E32. SDiD Overall and Event Time Annual Professional Spending ATT Estimates (PCP Only)

| **Group** | **Coefficient** | **Std. Error** | **Lower CI** | **Upper CI** |
| --- | --- | --- | --- | --- |
| Overall ATT | 298.90 | 172.24 | -40.31 | 743.52 |
| 5 years prior to integration | 64.97 | 16.31 | 29.48 | 97.19 |
| 4 years prior to integration | -29.29 | 10.83 | -59.55 | -12.30 |
| 3 years prior to integration | -4.60 | 6.12 | -16.99 | 9.09 |
| 2 years prior to integration | -7.66 | 6.85 | -25.23 | 6.53 |
| 1 year prior to integration | 1.52 | 3.30 | -5.07 | 8.42 |
| First integration year | -59.41 | 116.33 | -247.91 | 148.30 |
| One year after integration | 560.00 | 247.46 | 73.61 | 1183.82 |
| Two years after integration | 575.16 | 316.56 | 2.29 | 1219.34 |
| Three years after integration | 617.16 | 500.47 | -313.50 | 1729.74 |

Table E33. SDiD Overall and Event Time Annual Professional Spending ATT Estimates (without 2021 Integration Cohort, PCP Only)

| **Group** | **Coefficient** | **Std. Error** | **Lower CI** | **Upper CI** |
| --- | --- | --- | --- | --- |
| Overall ATT | 439.24 | 162.88 | 150.86 | 693.72 |
| 4 years prior to integration | -29.72 | 19.22 | -65.78 | 2.86 |
| 3 years prior to integration | 3.51 | 10.15 | -15.99 | 21.51 |
| 2 years prior to integration | 3.67 | 4.97 | -2.82 | 13.53 |
| 1 year prior to integration | -0.57 | 2.92 | -4.45 | 5.35 |
| First integration year | 146.64 | 145.16 | -135.63 | 337.68 |
| One year after integration | 560.75 | 161.36 | 316.30 | 809.33 |
| Two years after integration | 575.36 | 312.61 | -64.77 | 1131.31 |
| Three years after integration | 617.07 | 472.92 | -304.20 | 1248.43 |

Table E34. SDiD Overall and Event Time Annual Pharmaceutical Spending ATT Estimates (PCP Only)

| **Group** | **Coefficient** | **Std. Error** | **Lower CI** | **Upper CI** |
| --- | --- | --- | --- | --- |
| Overall ATT | 536.37 | 315.57 | 244.75 | 1186.51 |
| 5 years prior to integration | 43.28 | 34.58 | -36.27 | 79.57 |
| 4 years prior to integration | 80.71 | 32.35 | 57.15 | 157.96 |
| 3 years prior to integration | 11.57 | 10.52 | -7.84 | 28.35 |
| 2 years prior to integration | 20.79 | 11.04 | 15.72 | 52.99 |
| 1 year prior to integration | -10.54 | 5.80 | -21.21 | -0.16 |
| First integration year | 288.06 | 292.13 | -196.50 | 763.94 |
| One year after integration | 468.16 | 384.12 | -37.57 | 1261.61 |
| Two years after integration | 1278.17 | 510.43 | 331.40 | 2149.94 |
| Three years after integration | 398.42 | 613.69 | -427.95 | 1605.29 |

Table E35. SDiD Overall and Event Time Annual Pharmaceutical Spending ATT Estimates (without 2021 Integration Cohort, PCP Only)

| **Group** | **Coefficient** | **Std. Error** | **Lower CI** | **Upper CI** |
| --- | --- | --- | --- | --- |
| Overall ATT | 699.20 | 395.54 | 19.54 | 1501.04 |
| 4 years prior to integration | -1.60 | 19.38 | -47.39 | 25.73 |
| 3 years prior to integration | -0.94 | 9.63 | -15.19 | 13.51 |
| 2 years prior to integration | -0.66 | 5.66 | -13.65 | 4.49 |
| 1 year prior to integration | 0.44 | 2.67 | -6.01 | 3.53 |
| First integration year | 634.11 | 182.95 | 378.16 | 962.15 |
| One year after integration | 468.76 | 536.71 | -668.27 | 1400.32 |
| Two years after integration | 1279.24 | 586.36 | 798.78 | 2776.70 |
| Three years after integration | 401.59 | 872.92 | -1661.24 | 1298.48 |
